# Positive feedback induces switch between distributive and processive phosphorylation of Hog1

**DOI:** 10.1038/s41467-023-37430-y

**Published:** 2023-04-29

**Authors:** Maximilian Mosbacher, Sung Sik Lee, Gilad Yaakov, Mariona Nadal-Ribelles, Eulàlia de Nadal, Frank van Drogen, Francesc Posas, Matthias Peter, Manfred Claassen

**Affiliations:** 1 Department of Biology, Institute of Biochemistry, ETH Zurich, Zurich, Switzerland; 2 Department of Biology, Institute of Molecular Systems Biology, ETH Zurich, Zurich, Switzerland; 3 Scientific Center for Optical and Electron Microscopy, ETH Zurich, Zurich, Switzerland; 4 Department of Medicine and Life Sciences (MELIS), Universitat Pompeu Fabra (UPF), 08003 Barcelona, Spain; 5 Institute for Research in Biomedicine (IRB Barcelona), The Barcelona Institute of Science and Technology, Baldiri Reixac, 10, 08028 Barcelona, Spain; 6 Department of Computer Science, University of Tübingen, Tübingen, Germany; 7 Institute for Bioinformatics and Medical Informatics, University of Tübingen, Tübingen, Germany; 8 Present Address: Department of Molecular Genetics, Weizmann Institute of Science, Rehovot, Israel; 9 Present Address: Department of Internal Medicine I, Faculty of Medicine, University Hospital Tübingen, University of Tübingen, Tübingen, Germany

**Keywords:** Dynamic networks, Robustness, Computer modelling, Differential equations

## Abstract

Cellular decision making often builds on ultrasensitive MAPK pathways. The phosphorylation mechanism of MAP kinase has so far been described as either distributive or processive, with distributive mechanisms generating ultrasensitivity in theoretical analyses. However, the in vivo mechanism of MAP kinase phosphorylation and its activation dynamics remain unclear. Here, we characterize the regulation of the MAP kinase Hog1 in *Saccharomyces cerevisiae* via topologically different ODE models, parameterized on multimodal activation data. Interestingly, our best fitting model switches between distributive and processive phosphorylation behavior regulated via a positive feedback loop composed of an affinity and a catalytic component targeting the MAP kinase-kinase Pbs2. Indeed, we show that Hog1 directly phosphorylates Pbs2 on serine 248 (S248), that cells expressing a non-phosphorylatable (S248A) or phosphomimetic (S248E) mutant show behavior that is consistent with simulations of disrupted or constitutively active affinity feedback and that Pbs2-S248E shows significantly increased affinity to Hog1 in vitro. Simulations further suggest that this mixed Hog1 activation mechanism is required for full sensitivity to stimuli and to ensure robustness to different perturbations.

## Abstract (web summary)

How cells shape signalling dynamics in MAPK cascades remains unclear. Here the authors combine mathematical modelling with in vivo validation to uncover a novel feedback mechanism that increases processivity and robustness of the yeast Hog1 module.

## Introduction

**Fig. 1:**
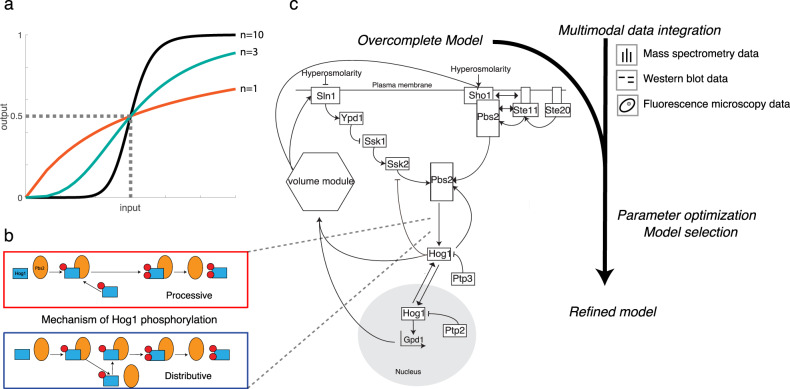
Generation of an overcomplete model of the Hog1 pathway and parameter optimization. **a** A Hill function quantifies input-output behavior as highly ultrasensitive (black line, Hill coefficient = 10), mildly ultrasensitive (green line, Hill coefficient = 3), or strictly Michaelian (red line, Hill coefficient = 1). EC50 value (dashed line) indicates the input strength at which 50% of the maximal output is reached. **b** The choice of Hog1 phosphorylation mechanism has a significant impact on the resulting input-output behavior and can be mainly processive or distributive. For the processive mechanism, the second phosphorylation step immediately follows the first without Hog1 disassociating from Pbs2. For the distributive mechanism Hog1 disassociates from Pbs2 after every phosphorylation step and needs to re-bind for further phosphorylation to take place. **c** Simplified scheme of the workflow to distinguish between different Hog1 phosphorylation mechanisms. The overcomplete ODE model allows enumeration of varying model topologies that differ in negative and positive feedback mechanisms, and Hog1 phosphorylation mechanism (such as described in Fig. 1b). Multimodal data were collected from literature or was newly self-generated, including fluorescence microscopy, mass spectrometry and western blot experiments. The data reflects various experimental conditions such as different salt concentrations and pulse frequencies, deletion mutants and kinase inhibition. Data sets were used to optimize the different submodels parameter values of the overcomplete model, thus giving rise to refined models from which the best fitting was selected.

Pathways integrating extracellular inputs often display an ultrasensitive response, in which beyond an input threshold, small changes in the input lead to large changes in the output. This behavior results in an essentially binary response, acting as a switch in the overall signaling cascade (Fig. 1a)^1^. Ultrasensitivity has been experimentally observed in various signaling systems and plays an important role in cellular decision-making^2^. Theoretical studies suggest that multi-tiered multisite phosphorylation cascades are inherently able to create ultrasensitivity^3^, with even single multisite phosphorylation resulting in ultrasensitivity and bistability^4^. In particular, the specific type of phosphorylation mechanism can alter signal response dynamics^5^. For dual phosphorylation, two distinct mechanisms are recognized (Fig. 1b). A distributive mechanism involves two consecutive reaction events, with kinase and substrate dissociating after each phosphorylation step, while for a processive mechanism, two phosphorylation reactions are induced in a single concerted reaction event^5^. Dual phosphorylation is a particularly widespread mechanism involved in activating mitogen-activated protein (MAP) kinases, which regulate the cellular responses to many intra- and extracellular signals. However, investigating the impact of different phosphorylation mechanisms is challenging and normally relies on mathematical models integrating typically difficult-to-measure temporal dynamics of specific protein species. The majority of theoretical studies have reduced to a dichotomy of assumptions - either distributive or processive - with distributive mechanisms associated with ultrasensitivity and processive mechanisms with a graded, non-ultrasensitive response^6^. Thus, to date, theoretical studies generally suggest that ultrasensitive kinase phosphorylation events in vivo should be governed by a distributive phosphorylation mechanism^3,7^, and some experimental data from mammalian cells support this hypothesis^8,9^. However, in more complex cases of multisite phosphorylation with more than two phospho-sites, behaviors have been observed that are not well explained by either a processive or distributive mechanism^10^. Some of these multisite phosphorylation events involve different kinases with distinct kinetic properties, which may complicate the analysis^11^. Altogether, understanding phosphorylation mechanisms governing ultrasensitive responses has proven particularly challenging in cases of multisite phosphorylation and the presence of positive feedback^12,13^. Additional studies are required to understand the degree to which different mechanisms interact, as well as the theoretical potential and experimental validation of a mixed phosphorylation mechanism.

To address these questions, we choose to investigate the mechanisms conferring ultrasensitivity in the High Osmolarity Glycerol (HOG) pathway in *Saccharomyces cerevisiae*, a well-studied MAP-kinase pathway which requires dual phosphorylation of the MAP kinase Hog1. Hog1 activation is needed to re-establish the balance between internal and external pressures upon osmotic shock. Upon exposure of cells to high osmolarity conditions, the two membrane-localized osmo-sensors Sho1 and Sln1 activate either the MAPKKKs Ste11 or Ssk2 and Ssk22, which converge on the MAPKK Pbs2 (Fig. 1c). Activated Sho1 recruits Pbs2, which acts both as a MAPKK to phosphorylate the MAP kinase Hog1 and as a scaffold recruiting other upstream kinases including its own activator Ste11. Additionally, Ste50 triggers full Ste11 activation by recruiting various co-stimulators to the cell membrane^14,15^. The partially redundant Sln1 branch uses a histidine phospho-relay system, which inhibits the kinase Ssk2 in the absence of osmotic stress through the intermediate histidine phosphate transfer protein Ypd1^16^. Upon Sln1 inactivation in response to osmotic stress, Ssk1 induces the auto-phosphorylation of the MAPKKKs Ssk2 and Ssk22. Like Ste11, these interact and phosphorylate Pbs2, which in turn doubly phosphorylates Hog1, leading to its rapid translocation into the nucleus to launch a transcriptional program. In addition to altered gene expression, particularly induction of Gpd1^17^, Hog1-mediated cytoplasmic changes such as the closure of water channels are of great importance to rapidly reestablish osmotic balance^18^. Dephosphorylation and inactivation of Hog1 is carried out by an array of phosphatases that includes the tyrosine phosphatases Ptp2 and Ptp3 in the nucleus and cytoplasm respectively, and the Ser/Thr phosphatases Ptc1 and Ptc2/3^19–24^.

The HOG pathway has previously been used to study MAPK cascades as the topology and molecular functions of its components have largely been established^25^. Hog1 activation can be measured by a variety of methodologies including western blot^26^, fluorescence microscopy^27,28^ and mass spectrometry^29,30^. Taking advantage of such data sets, previous studies established mechanistic models of the whole HOG pathway^31^ or the role of different sub-branches in homeostasis^32^, and analyzed the impact of upstream phosphorylation^33^ or glycerol accumulation^34^ on pathway adaptation. However, experimental data and modeling approaches mechanistically describing Hog1 dual phosphorylation and the relevance of feedback loops for Hog1 activation are still scarce.

In this study, we examined the molecular mechanisms responsible for ultrasensitivity in the HOG pathway. We used an integrative modeling approach taking advantage of various data sources and experimental parameters to compare Hog1 activity and its phosphorylation status under multiple environmental conditions such as varying salt concentrations and salt pulses, and different genetic mutations that specifically perturb Hog1 activation kinetics. Interestingly, our findings support a mixed distributive and processive phosphorylation model as the best fit for the observed experimental behavior, with a critical positive feedback loop targeting the MAPKK Pbs2.

## Results

### Construction of a HOG pathway ODE model comprising putative negative and positive feedback, with different mechanisms of Hog1 activation by double phosphorylation

To understand the molecular mechanism responsible for ultrasensitivity in the HOG pathway (Fig. 1a), we examined whether the MAP kinase Hog1 is activated by a distributive or processive phosphorylation mechanism (Fig. 1b). When a basic, three-tiered MAPK module without additional feedback mechanisms is fitted on MAPK activity input-output data ranging from graded to ultrasensitive with unconstrained parameter ranges, we found that parameter sets could be identified that recapitulate the bi-stable behavior irrespective of the phosphorylation mechanism used (Supp Fig. S1). We thus constructed an overcomplete, deterministic model based on ordinary differential equations (ODEs) for the ultrasensitive, osmostress-induced Hog1 phosphorylation that allowed evaluating various submodel topologies differing in Hog1 phosphorylation and feedback mechanisms (Fig. 1c). Taking advantage of previous studies^28^, we started from a set of reactions termed the cell volume module. The module links intracellular glycerol concentration, which includes retention and production of glycerol, to pressure parameters, describing external osmotic pressure and internal turgor pressure, which in turn determine cellular volume changes (Fig. 1c, description in Supp Table S7). Downstream effector mechanisms that lead to increased glycerol production and volume adaptation were simplified compared to previous modeling approaches^31^ to reduce complexity in areas of the model that are not relevant to explain ultrasensitivity. The model considers both Sho1 and Sln1 branches of the HOG pathway, and the shuttling of Hog1 between a cytosolic and a nuclear compartment. Moreover, it takes into account known and putative feedback regulation, including Hog1-mediated phosphorylation of the upstream components Sln1, Ssk1, Ssk2 and the scaffolding kinase Pbs2^35^. Importantly, the model includes both mono- and bi-phosphorylated species of Hog1, and thus allows distinguishing processive and distributive mechanisms for Hog1 activation. In the processive model, the second Hog1 phosphorylation step immediately follows the first without Hog1 dissociating from its scaffold Pbs2, while in the distributive activation model Hog1 detaches from Pbs2 after the first phosphorylation step and thus needs to rebind to allow the formation of the doubly phosphorylated, active species (Fig. 1b, Supp Box 1). Phosphatases responsible for Hog1 dephosphorylation (Ptp2, Ptp3, Ptc1, and Ptc2/3) and their mechanisms were implemented^19–24^ to account for their possible impact on Hog1 activation kinetics. The resulting model thus not only provides a detailed representation of the topology and assembly intermediates upstream of Hog1, but also accounts for different Hog1 activation mechanisms and for positive and negative feedback regulation.

### Multimodal data integration and model selection favor distributive over processive mechanism of Hog1 phosphorylation modulated by both positive and negative feedback loops

To infer topology and parameters of the reaction model, we considered experimental data from multiple literature sources, as well as own measurements in wild-type and mutant strains exposed to stepwise increase of NaCl of varying concentrations (Fig. 1c). The data include population- and single-cell measurements directly or indirectly reporting on Hog1 activity at different time points after stimulation (see Supp Tables S1 and S2 for a list and description of considered datasets). For example, mass spectrometry measurements inform about relative changes in double phosphorylated Hog1 within the first 60 s of the signaling response as well as at later time points^29,30^. These data were complemented by western blot measurements with antibodies recognizing doubly phosphorylated Hog1 with conditions including strains lacking different upstream components or the Ptp2 and Ptp3 phosphatases, as well as inhibition of Hog1 activity by small molecule inhibitors^20,26,36^. Moreover, Hog1 activity correlates with its nuclear translocation, which can be quantified by fluorescence microscopy in single cells^27^. Using a microfluidic platform, we performed extensive Hog1 activity measurements in wild-type and *pbs2Δ* cells exposed to various NaCl concentrations and NaCl ramping perturbations. Finally, the volume module was independently parameterized with cell area measurements upon various salt treatments of wild-type and *pbs2Δ* cells. The latter provides information on Hog1 independent mechanisms that lead to volume adaptation that are also considered in the model (Supp Fig. S2).

**Fig. 2:**
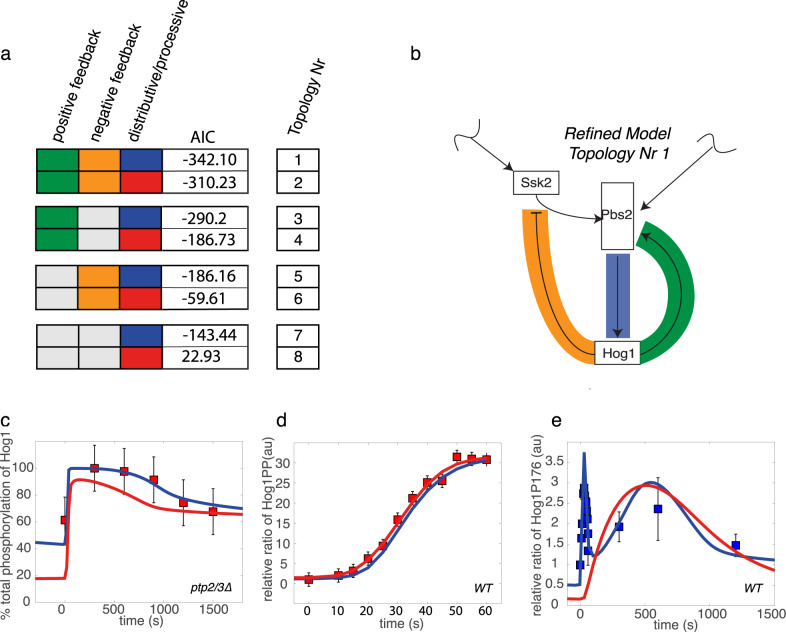
Refined model employs negative and positive feedback and favors a distributive over a processive phosphorylation mechanism. **a** Eight different topologies were enumerated with different combinations of negative feedback (orange), positive feedback (green) and distributive (blue) or processive (red) Hog1 phosphorylation mechanism. Akaike Information Criterion (AIC) of the best model fits are displayed. **b** Scheme of refined model (according to pipeline described in Fig. 1c) with topology one. Negative feedback on Ssk2 and positive feedback on Pbs2 are indicated in orange and green respectively. The distributive phosphorylation mechanism of Hog1 by Pbs2 is visualized in blue. Simulations of selected species of the two best-fitting models employing distributive or processive Hog1 phosphorylation (Topology Nr 1 and 2) are shown in response to salt stimulation (0.4 M NaCl). Graphs show experimental data points used for parameter optimization (red square), experimental data points used for testing (blue square), simulation results from the best-fitting distributive mechanism (blue) and simulation data from the best-fitting processive mechanisms (red). **c** Time course of the total phosphorylation of Hog1 in percent (%) in a Ptp2/3 deleted condition. Data by Jacoby et al. are presented as quantified values ± computational estimate of SEM of *n* = 1 independent experiment^20^. **d** Time course of the relative ratio between stimulated and basal levels of dual phosphorylated Hog1 in wild type (WT) cells exposed to 0.4 M NaCl. Data by Kanshin et al. are presented as values of peptide fold change ± computational estimate of SEM of *n* = 1 independent experiment^30^. **e** Time course of relative ratio between stimulated and basal levels of mono-phosphorylated Hog1-P176 in a WT cells exposed to 0.4 M NaCl. Data for the first 60 s by Kanshin et al. are presented as values of peptide fold change and data by Vaga et al are presented as mean values of peptide fold change ± standard deviation of *n* = 3 independent experiments^29,30^. Data were not used for fitting. Note that the processive mechanism does not show the double peak in the data, characteristic of the distributive mechanism. Source data are provided as a Source Data file.

We used 533 data points across the different conditions and perturbations to estimate the relevant model parameters. We enumerated eight models with distinct topologies varying in Hog1 phosphorylation mechanism and absence or presence of positive and negative feedback loops (Fig. 2a). Between 63 and 86 parameters were undetermined in the considered models and thus fitted via likelihood optimization (see Method section for details).

We first aimed to determine the mechanism of Hog1 activation and evaluate the importance of positive and negative feedback loops. Thus, we performed parameter optimization followed by selection of model variants comprising all combinations of distributive or processive mechanisms of Hog1 phosphorylation, and the presence or absence of positive and negative feedback regulation. The Akaike Information Criterion (AIC) was used to compare and rank the results (Fig. 2a, Supp Box 2). In general, an AIC difference between two competing models of greater than ten is considered highly significant^37^. The overall best fit was achieved with distributive Hog1 phosphorylation and positive and negative feedback mechanisms, and the AIC difference was significant compared to a processive mechanism. Accordingly, we defined a refined model (Fig. 2b). The AIC difference between models utilizing distributive or processive mechanism was most readily apparent in cells deleted for Ptp2 and Ptp3 (Fig. 2c). In this case the processive model was unable to recapitulate the full increase in basal signaling as well as the complete activation upon salt stress.

On the other hand, certain temporal patterns such as the dynamics of double phosphorylated Hog1 in wild-type cells in the first 60 s of salt stress, were better approximated by a model employing a processive phosphorylation mechanism (Fig. 2d). This observation suggests that properties such as the regulation of basal activation levels and maintenance of the full range of activation need a more distributive Hog1 phosphorylation mechanism, whereas certain dynamic properties such as the aforementioned rapid double phosphorylation of Hog1 could more easily be achieved by a processive mechanism.

Simulations of mono-phosphorylated Hog1 dynamics revealed significant qualitative differences upon simulation with different phosphorylation mechanisms. Simulations with a distributive phosphorylation mechanism resulted in a temporal profile marked by a double peak, with a first activity peak during the initial minutes and a second, lower increase towards the end of the response (Fig. 2e). In contrast the processive model simulation displayed monophosphorylated Hog1 dynamics with a single peak reminiscent of the behavior of doubly phosphorylated Hog1. Importantly, comparison with an experimental test data set of monophosphorylated Hog1 corroborated the distributive nature of the reaction (Fig. 2e).

### A mixed phosphorylation mechanism best explains Hog1 activation kinetics by integrating favorable dynamic properties of both distributive and processive mechanisms

**Fig. 3:**
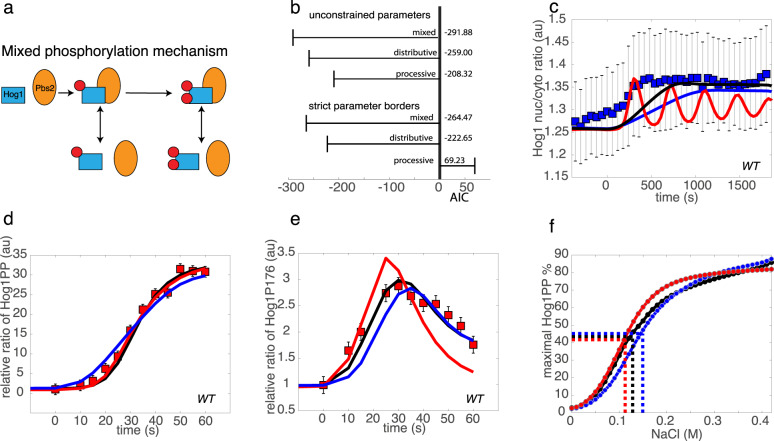
Temporal dynamics of monophosphorylated Hog1 support models with partially distributive phosphorylation. **a** A mixed phosphorylation mechanism generates a phosphorylation reaction with both distributive and processive character. **b** Akaike information criterion (AIC) of the best fits after the optimization and fitting with unconstrained parameter values or stricter boundaries that approximate physiological conditions (see Supp Fig. S4). Note, that in contrast to the refined model of Fig. 2b, data from mono-phosphorylated Hog1 species was used in the fitting and the resulting fit thus the best fitting model on all data. Simulations are shown of selected Hog1 species from the three best fitting models in which all models incorporate positive and negative feedback in wild-type cells (WT). Graphs show experimental data points used for parameter optimization (red square) and testing (blue square), as well as simulation results from the best fitting mixed (black line), processive (red line) or distributive (blue line) Hog1 phosphorylation mechanism in response to salt stimulation. **c** Time course of Hog1 nuclear to cytosolic ratio in cells exposed to linear ramping up to 0.2 M NaCl. Data are presented as mean values ± standard deviation of n = 95 cells over 2 independent experiments. Note that the experimental data (blue squares) was not used in the fitting procedure. Time course following salt stimulation (0.4 M NaCl) of mono-phosphorylated Hog1-P176 (**d**) and dual phosphorylated Hog1-PP (**e**) expressed as a relative ratio between stimulated and basal levels. Data by Kanshin et al are presented as values of peptide fold change ± computational estimate of SEM of *n* = 1 independent experiment^30^. **f** Fit of a Hill function to the simulated input–output curves of the best fitting mixed (black line), processive (red line) or distributive (blue line) Hog1 phosphorylation mechanism with a Hill coefficient of 2.39, 3.03 and 2.77, respectively. EC50 values of 0.11 M, 0.13 M, and 0.15 M for processive, mixed, and distributive mechanism respectively, are indicated by dashed lines. Source data are provided as a Source Data file.

While distributive Hog1 activation leads to a significantly better goodness-of-fit, the processive mechanism better recapitulates some data such as speed and degree of double phosphorylated Hog1 accumulation. Thus, we next evaluated a mixed phosphorylation mechanism that incorporates characteristics from both the processive and distributive mechanisms. This mixed mechanism allows for both processive double phosphorylation and distributive dissociation of mono-phosphorylated species (Fig. 3a; Box 1). Thus, mono-phosphorylated Hog1 has a certain propensity, defined by the kinetic rate constant, to remain Pbs2 bound and immediately undergo a second phosphorylation step, in which case we observe processive characteristics. Alternatively, mono-phosphorylated Hog1 can dissociate from Pbs2 resembling a distributive mechanism. To assess whether such a mixed model would indeed recapitulate the above-mentioned temporal patterns of Hog1 activation, we repeated the parameter optimization procedure by including mass spectrometry data sets of monophosphorylated Hog1, the results of which we defined as best-fitting models^29,30^ (Supp Fig. S3). Interestingly, AIC measurements revealed that the optimized model employing a mixed phosphorylation mechanism showed significantly better goodness-of-fit compared to either the distributive or processive variants, with all models incorporating positive and negative feedback (Fig. 3b).

We simulated the best fitting distributive, processive or mixed models to predict Hog1 activation upon a linear ramping increase of NaCl concentration, comparing simulation output to an independent data set not used for parameter optimization (Fig. 3c). Monitoring Hog1 nuclear translocation dynamics to approximate Hog1 activation, the processive mechanism leads to premature Hog1 translocation and displays an inappropriate oscillating behavior, while the distributive mechanism results in a delay of Hog1 translocation. Only the mixed model was able to fine-tune the Hog1 nuclear translocation kinetics with an appropriately rapid yet stable response.

Indeed, the processive mechanism produces simulations with premature accumulation of activated Hog1 manifested by the appearance of doubly phosphorylated Hog1 in the first 60 s after salt addition (Fig. 3d). We assume that this behavior addresses the need to compensate direct mono-phosphorylation by employing faster accumulation of double phosphorylated Hog1 that can then undergo dephosphorylation to generate the pool of mono-phosphorylated species. Case in point, the simulated accumulation of mono-phosphorylated Hog1 by the processive model was slower and not as high as expected from experimental data (Fig. 3e). On the other hand, the distributive model resulted in faster accumulation of mono-phosphorylated species than measured experimentally and its decay began earlier than expected (Fig. 3e). However, the mixed phosphorylation mechanism shows a significantly better fit and is situated between the two extreme cases, and thus as predicted compensates for both premature accumulation and decay observed with distributive Hog1 activation and the delay of the processive mechanism (Fig. 3d, e). Upon fitting Hill functions to our simulated input-output curves, we observed that increase in processivity led to lower EC50 values, with the mixed mechanism achieving a lower value than a distributive and a processive mechanism. We also quantified ultrasensitivity using the Hill coefficient (Fig. 3f). Interestingly, the processive mechanism resulted in the highest Hill coefficient, while as expected, the mixed mechanism showed lower ultrasensitivity than the distributive mechanism.

We further considered physiological parameter boundaries more closely reflecting known parameter values of general yeast kinases and phosphatases. Within these boundaries the mixed phosphorylation mechanism resulted in an even more significant difference in AIC compared with the extreme mechanisms (Fig. 3b). For example, with physiological parameter boundaries, the processive mechanism was once again unable to recapitulate the double peak behavior of mono-phosphorylated Hog1 and experimental data specific to the Sho1 sub-branch of the pathway (Supp Fig. S4).

### Hog1-dependent positive feedback increases processivity of the Hog1 phosphorylation reaction

**Fig. 4:**
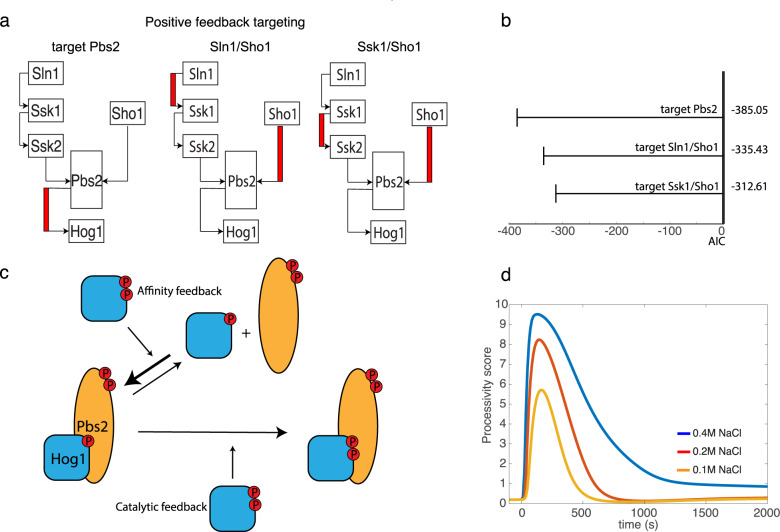
Two-component positive feedback to Pbs2 gives best goodness of fit and results in increase of processivity. **a** Schematic of three models with potential targets for positive feedback upstream of Hog1, showing the positive feedback highlighted in red for models where activated Hog1 phosphorylates either Pbs2 (left), Sln1 and Sho1 (middle), or Ssk1 or Sho1 (right). **b** Akaike information criterion (AIC) was used to order optimized models with the indicated positive feedback targets, in which all models include negative feedback and a mixed Hog1 phosphorylation mechanism. Positive feedback targeting Pbs2 achieves best AIC among other putative targets. **c** Positive feedback of activated Hog1 to Pbs2 results in an increase of the kinetic rate constant for the activation of Hog1 (catalytic feedback) and an increase in the association of Hog1 to Pbs2 (affinity feedback). **d** Time course upon salt stimulation showing a processivity score that quantifies the ratio between the rate of the second Hog1 phosphorylation step and its dissociation from Pbs2. A low processivity score indicates distributive-like phosphorylation, while a high processivity score indicates a processive-like mechanism. Note that before starting the reaction the processivity score is low, but the onset of Hog1 signaling introduces positive feedback (*t* = 0), which increases the score until the reaction behaves like a processive mechanism.

Next, we investigated in more depth the mechanism, effects and potential targets of the positive feedback, and their relationship to the mixed mechanism of Hog1 phosphorylation. Indeed, phosphoproteomic measurements revealed that eight out of eleven components involved in the Hog1 pathway undergo phosphorylation upon salt exposure^29^. We focused on putative feedback loops that phosphorylate targets upstream of Hog1. In the Sln1 sub-branch, the data suggests that Ssk1 and Ssk2 are potential Hog1 substrates, while in the Sho1 sub-branch Hog1 phosphorylates Ste50 and Ste20. However, phosphorylation of Ssk2^35^ and Ste50^38^ interfere with Hog1 activation, making them unlikely physiological candidates. Moreover, positive feedback must target both sub-branches simultaneously to explain the experimental data. Only Hog1-dependent phosphorylation of Pbs2 would fulfill this requirement, as Pbs2 integrates the information of both sub-branches (Fig. 4a). Alternatively, we considered Ste20 and either Sln1 or Ssk1 as potential targets of positive feedback. However, a mixed model that incorporates positive feedback of Hog1 on Pbs2 showed significantly better results than topologies in which Hog1 targets Sln1 and Ste20 or Ssk1 and Ste20 (Fig. 4b).

Positive feedback mediated via Pbs2 has two distinct components. First, it affects the kinetic rates of Hog1 phosphorylation, which we refer to as catalytic feedback, and second, it controls the affinity of the Hog1-Pbs2 association, an effect we refer to as affinity feedback (Fig. 4c). Importantly, this two-component feedback mechanism leads to significantly better fitting results compared to a single component catalytic feedback model, and suggest that small changes in Hog1-Pbs2 association of the affinity feedback are important to explain the data (Supp Fig. S5). In part due to this dichotomy the positive feedback has two interesting effects. Firstly, it increases the number of Hog1 phosphorylation events per unit of time, as expected for positive feedback. Secondly, it also leads to increased processivity of the Hog1 phosphorylation reaction by enhancing the speed of the second Hog1 phosphorylation reaction via the changes in the Hog1-Pbs2 affinity, with comparatively minor effects on the first phosphorylation reaction. Processivity in the mixed Hog1 activation model is formally defined by the ratio between the rate of the second phosphorylation step and the rate of dissociation of mono-phosphorylated Hog1 bound to activated Pbs2. To assess processivity over the course of the response we introduced a processivity score (see Method section), which quantifies the rate of an immediate second phosphorylation step compared to dissociation of the Hog1-Pbs2 complex. Interestingly, the simulations showed that in the best fitting model with positive feedback on Pbs2, this score is low before and after the response to salt stimulation and the Hog1 phosphorylation mechanism displays a behavior mimicking a distributive mechanism (Fig. 4d). The onset of positive feedback activity during the response leads to a significant increase in the score, meaning the reaction becomes highly processive. In comparison, the best-fitting result of the model targeting Sln1 does not result in significant changes of processivity. The Ssk1 model displayed increased processivity along the course of the reaction but the basal level of processivity was orders of magnitude higher than the better-fitting model targeting Pbs2.

Taken together, these results indicate that a mixed Hog1 phosphorylation mechanism enables a switch from a more distributive to a more processive activation mechanism. Importantly, this switch is most likely regulated by affinity feedback at the level of Pbs2, increasing Hog1-Pbs2 association.

### Pbs2-S248 is phosphorylated by Hog1 in vitro and S248 mutants affect the affinity feedback of Hog1 activation

**Fig. 5:**
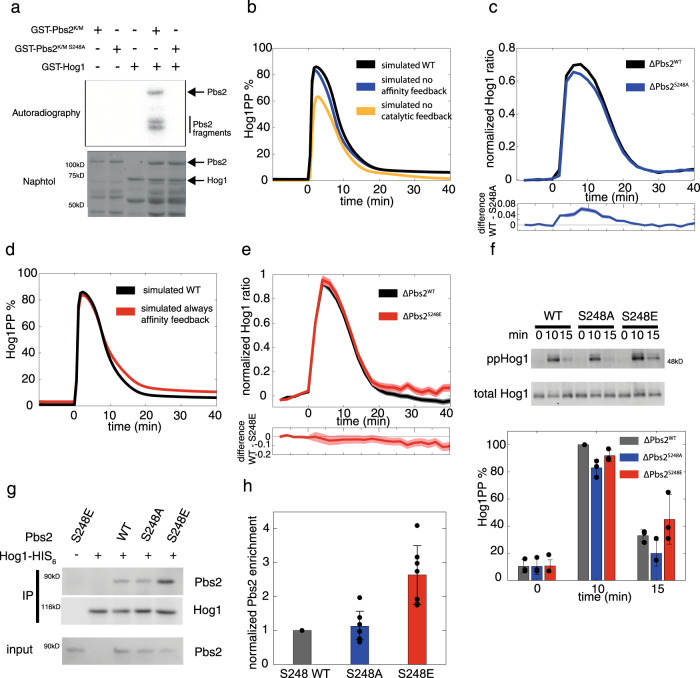
Pbs2-S248 is phosphorylated by Hog1 in vitro and affects affinity feedback of Hog1 activation in vivo. **a** Pbs2 is phosphorylated in vitro by Hog1 at Ser248. Recombinant catalytically inactive GST-Pbs2 K/M proteins were purified from *E. coli* and incubated with GST-Hog1 purified from NaCl treated wild type yeast (where indicated) in kinase buffer containing ATP. Pbs2 K/M or Pbs2 K/M^S248A^ was then added in the presence of radioactive ATP. Phosphorylated proteins were resolved by SDS–PAGE, stained (lower panel) and dried before detection by autoradiography (upper panel). **b** Simulated time lapse of the double phosphorylation of Hog1 upon addition of 0.4 M NaCl after 500 s. The dynamics simulated by the best fitting model with mixed phosphorylation mechanism (black line) were declared as wild type and compared to the simulated time course by the same model with either the affinity component (blue line) or the catalytic component (yellow line) of the positive feedback on Pbs2 abolished. **c** Data are presented as mean Hog1 nuclear to cytosolic ratio ± SEM of a ΔPbs2^WT^ strain (black line) with *n* = 4620 cells over three independent experiments and a ΔPbs2^S248A^ strain (blue line) with *n* = 5103 cells over three independent experiments upon addition of 0.4 M NaCl at time point 0. The lower panel shows the difference of the mean of ΔPbs2^WT^ and ΔPbs2^S248A^ and the standard error of the mean. **d** Simulated time lapse of the double phosphorylation of Hog1 upon addition of 0.4 M NaCl at timepoint 0. The dynamics simulated by the best fitting model with mixed phosphorylation mechanism (black line) were declared as wild type and compared to the simulated time course by the same model with the affinity component constitutively activated (red line). **e** Data are presented as mean Hog1 nuclear to cytosolic ratio ± SEM of a ΔPbs2^WT^ strain (black line) with *n* = 3279 cells over three independent experiments and a ΔPbs2^S248E^ strain (red line) with *n* = 1158 over three independent experiments upon addition of 0.4 M NaCl at timepoint 0. Lower panel shows the difference of the mean of ΔPbs2^WT^ and ΔPbs2^S248E^ and the standard error of the mean. **f** Hog1 phosphorylation was assessed by western blot using extracts prepared from the indicated strains exposed to 0.4 M NaCl after 0, 10, and 15 min. Data are presented as mean values ± standard deviation with *n* = 3 independent experiments. **g** Hog1-HIS was purified from *E. coli* and retained in HIS beads followed by incubation with NaCl-stressed yeast extracts carrying Pbs2 (WT), Pbs2 ^S248A^ (A), Pbs2 ^S248E^ (E) or *pbs2Δ* (Δ) in a *hog1Δ* background (10 min 0.4 M NaCl). Pbs2-Hog1 interaction was monitored via Western Blot. **h** Data are presented as mean of fold change mutants respect to WT (S248) which was set to 1 as a reference ± standard deviation of seven independent experiments. Source data are provided as a Source Data file.

Next, we attempted to determine the target of the positive feedback and to find out what effect its disruption might have on the two putative effects, the change in Hog1 processivity and the significant increase in reaction speed. Pbs2 has a number of phosphorylation sites that could act as potential sites of regulation, including S248 which is phosphorylated in response to high osmolarity^29,30^. Importantly, this serine is followed by a proline residue and thus conforms to the minimal MAPK consensus motif (S/TP). Using autoradiography, we could indeed establish that Pbs2 is directly phosphorylated on Ser248 by activated Hog1 in vitro (Fig. 5a, Supp Fig. S6).

To exclude that phosphorylation of Pbs2 on S248 by Hog1 is essential for Pbs2 activity, we stably integrated wild-type Pbs2 or a non-phosphorylatable Pbs2-S248A mutant in a *pbs2Δ* strain (yHS206). In contrast to *pbs2Δ* controls, both strains were able to grow on plates containing 0.4 M NaCl, indicating that Hog1-dependent feedback on S248 is not essential for viability under these conditions (Supp Fig. S7). We then used simulations and kinetic experiments to validate that Pbs2-S248 phosphorylation is at least in part responsible for the positive feedback of Hog1 on Pbs2 suggested in the mixed model. Interestingly, maximal levels of doubly phosphorylated Hog1 were slightly or strongly reduced, respectively, when simulating Hog1 activation time courses upon salt treatment using the best-fitting mixed model with either the affinity or catalytic component of the positive feedback disrupted (Fig. 5b). Quantifying Hog1-RFP localization in salt-treated *pbs2-S248A* cells monitored in microfluidic devices revealed that its nuclear to cytoplasmic ratio was only slightly lower compared to WT controls (Fig. 5c), consistent with simulations in which Hog1-Pbs2 affinity feedback is disabled. While the effect on maximal Hog1 activity seems small, the difference is significant as highlighted by plotting the difference of the mean and standard error between the WT and the Pbs2-S248A mutant. The lower panel shows the difference of the mean of ΔPbs2^WT^ and ΔPbs2^S248A^ and the standard error of the mean. Conversely, we also investigated Hog1 activation dynamics in the case of constitutive activation of the affinity feedback, mimicked by a phosphomimetic Pbs2-S248E mutant. Corresponding simulations predict a minimal reduction of the peak level of doubly phosphorylated Hog1 but higher basal levels after the response compared to WT controls (Fig. 5d). Indeed, the nuclear to cytosolic ratio of Hog1-RFP in the Pbs2-S248E mutant strain confirmed higher basal levels of Hog1 activity after salt response (Fig. 5e), consistent with simulations in which Hog1-Pbs2 affinity feedback is constitutive. To further corroborate this microscopy analysis, we also assessed Hog1 double phosphorylation after addition of 0.4 M NaCl by immunoblotting in wild-type and Pbs2-S248A and Pbs2-S248E mutant strains, respectively (Fig. 5f, Supp Fig. S8). As expected, Hog1 double phosphorylation was slightly diminished after 10 min in Pbs2-S248A mutant cells, while basal activation levels remained slightly higher in Pbs2-S248E cells during salt adaptation (Fig. 5g).

Finally, to directly assess the impact of Hog1-dependent feedback phosphorylation of Pbs2-S248 on the processivity of the reaction, we performed pull-down assays to monitor the affinity between Hog1 and Pbs2-S248 mutants (Fig. 5h, Supp Fig. S9). Consistent with our predicted increase in processivity upon phosphorylation, phosphomimetic Pbs2-S248E showed significantly increased binding to Hog1 compared to WT Pbs2.

Taken together, although as predicted by the in silico simulations the effect of disrupting this affinity feedback for Hog1 activation dynamics is modest, both population and kinetic single-cell measurements, as well as affinity measurements via pull-down assays confirm that phosphorylation of Pbs2 on serine 248 is involved in switching Hog1 activation from a distributive to a processive mechanism.

### Mixed mechanism conveys robustness to protein-level fluctuations

We next more thoroughly evaluated robustness of the newly described Hog1 activation mechanism. Robustness is of particular importance in the case of Hog1 phosphorylation since its hyperactivation leads to severe growth defects, observed for example upon overexpression of Pbs2^39^. Indeed, 10x in silico overexpression of Pbs2 revealed that Hog1 was almost fully doubly phosphorylated and thus hyperactivated in the best fitting distributive and mixed models (Supp Fig. S10A). In contrast, overexpression of Pbs2 in the processive model prevented double phosphorylation of Hog1 (Supp Fig. S10B).

**Fig. 6:**
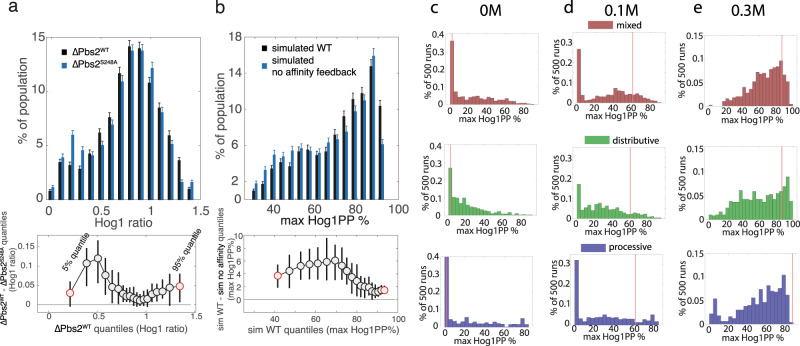
Mixed mechanism with positive feedback is more robust to protein concentration perturbations. **a** Histograms of the distribution of the maximal Hog1 nuclear to cytosolic ratio as a proxy for Hog1 activity for ΔPbs2^WT^ (black) and ΔPbs2^S248A^ strains (blue). Data are presented as percent of total cell population ± standard deviations computed using a bootstrap procedure. The difference in the distribution and its significance was assessed using a quantile shift function, plotting the difference of 5% to 95% quantiles of the Hog1 activation with a spacing of 5% against the quantile values of the ΔPbs2^WT^ strain. 95% confidence intervals computed via percentile bootstrap are indicated. **b** Histograms of the distribution of 2000 simulations of the best fitting model with mixed phosphorylation mechanism with starting protein concentrations randomly varied. The maximal percentage of double phosphorylated Hog1 after addition of 0.4 M NaCl is depicted for the WT model (black) and the same model with the affinity component of the positive feedback on Pbs2 abolished (blue). Data are presented as percent of total simulated runs ± standard deviations computed using a bootstrap procedure. As in (**a**) significance of the difference was established using a quantile shift function. Distribution of the maximal activation of Hog1 under different salt concentrations as a result of 500 simulations of the best fitting models of mixed (red), distributive (green) or processive (blue) phosphorylation mechanism with the involved protein species randomly varied in concentration by 0.5 to two times their physiological concentration. Simulation results without salt perturbation (**c**), with 0.1 M NaCl (**d**) or 0.3 M NaCl (**e**) added. Source data are provided as a Source Data file.

We can visualize the robustness of the response by analyzing potential differences in the population distribution of maximal Hog1 activity as measured for individual cells at a specific time point. Supp Fig. S11 illustrates how such differences in distribution manifest themselves via a hypothetical example, which ultimately can be assessed by so-called quantile shift functions (see Methods for details). Using this approach, we looked at the distribution of maximal Hog1 activation as the result of a response to 0.4 M NaCl measured in single cells via ratio of nuclear to cytosolic Hog1 fluorescence. Surprisingly, the distribution shows small but significant differences between wild type and Pbs2-S248 mutant cells with an increase of cells showing only intermediate activation (Fig. 6a). The statistical significance was determined and visualized using the well-established quantile shift function that captures the degree and location of the difference of two distributions by providing the quantile differences plotted against the quantile of the wild type distribution (Fig. 6a, Supp Fig. S12A)^40^.

To corroborate these results, we conducted in silico experiments in which expression levels of proteins in the Sln1 sub-branch and phosphatases (Sln1, Ssk1, Ssk2, Ste20, Pbs2, Ptp2, and Ptp3) were varied between half to twice their original concentration, mimicking the natural fluctuations of protein expression in single cells and providing a distribution of Hog1 activation. We performed 2000 simulations of a single-step addition of 0.4 M NaCl and noted the resulting maximal Hog1 double phosphorylation. This was carried out for wild-type cells with functioning feedback loops and repeated for a model in which the affinity feedback on Pbs2 was abolished. Consistent with the experimental results, loss of affinity feedback in the Pbs2-S248A mutant showed an increase in bimodality with a higher proportion of the cell population showing intermediate Hog1 activation between 20 and 60 percent Hog1 double phosphorylation, the significance of which was again quantified and visualized using a quantile shift function (Fig. 6b).

The absence of the affinity feedback reduces the processivity of the reaction making it more distributive. To generalize this behavior, we also simulated maximal Hog1 double phosphorylation upon 500 randomized perturbation schemes for the previously described best-fitting distributive, processive and mixed models. With no perturbation (0 M) or low NaCl concentrations (0.1 M), the processive mechanism resulted in slightly bimodal distributions with 2.4 times the number of perturbations generating complete activation (>70% Hog1 double phosphorylation) than the mixed mechanism, and the majority showing no activation. The distributive mechanism led to an accumulation of activation at an intermediate level (20%), while the mixed model distributed more around the unperturbed activation level (Fig. 6c, d). At higher levels of NaCl input (0.3 M), the processive mechanism failed to reach the maximal level of activation and displayed a disproportionate number of simulations that led to only very minimal activation. The distributive mechanism on the other hand, shows a more uniform distribution between 30 and 90% Hog1 activation. Strikingly, however, only the mixed mechanism predicted low basal levels of Hog1 activity, with reliable rapid and high activation upon exposure to the different NaCl conditions (Fig. 6e).

We thus conclude that the mixed Hog1 activation mechanism displays a unique and favorable system’s behavior that leads to increased robustness in the input-output relations, leading to decreased sensitivity of its output to initial intracellular conditions. This behavior is at least partially distinct from positive feedback that increases the overall reaction speed.

## Discussion

Effective processing of external information via subsequent intracellular response is of vital importance for every cell. From a systems-engineering perspective, it is desirable for this process to be robust towards external and internal state fluctuations. Similarly, the actual input threshold to induce a cellular response should be carefully chosen to avoid unnecessary energy expenditure. In the *S. cerevisiae* HOG pathway, Hog1 is activated by dual phosphorylation and thus the choice and kinetics of this phosphorylation mechanism can greatly affect signaling dynamics. A multitude of previous, mostly theoretical, work has focused on the extreme cases of distributive or processive mechanisms. Interestingly, however, our combined experimental and modeling analysis revealed that a mixed control mechanism regulated by positive feedback best explains the observed phosphorylation and signaling dynamics.

Previous theoretical analyses of a simple three-tiered MAPK cascade predicted that such a mixed mechanism may enhance the tunability of the response^41^. In particular, increased processivity can enhance the sensitivity to the external signal and lower ultrasensitivity. Indeed, increasing processivity diminished EC50 values, with the mixed mechanism achieving a lower value than distributive and processive mechanisms (Fig. 3f). Moreover, measurements of the Hill coefficient confirmed that the mixed mechanism showed lower ultrasensitivity than distributive Hog1 activation. This is probably due to the processive mechanism plateauing earlier with a lower maximal activation than the two other mechanisms. This would indicate that while a processive mechanism can guarantee ultrasensitivity, the range of dynamics is lower than what can be achieved by a distributive mechanism.

A second impact of the different phosphorylation mechanisms was observed when evaluating the robustness of the pathway output. The mixed mechanism was more robust towards perturbation of protein concentrations, and also less prone to exaggerated activation at lower salt concentrations. We speculate that the distributive nature of Hog1 phosphorylation at basal levels or low salt stress serves as an additional checkpoint, where mono-phosphorylated Hog1 species are dephosphorylated faster than the second phosphorylation step and thus full Hog1 activation can occur. However, after a certain input threshold has been crossed at higher salt concentrations, the positive feedback increases the processivity of the reaction, reliably promoting maximal Hog1 activation and increasing robustness compared to a solely distributive mechanism. Mechanistically, increased and erroneous double phosphorylation of Hog1 due to perturbations could be dampened as heightened association with Pbs2 sequesters the MAPKK and thus prevents further activation. At the same time, the increase in processivity also serves as positive feedback, leading to efficient Hog1 activation even in the case of internal noise such as reduced Hog1 concentration. Reflecting this potential mechanism, distributing Hog1 activation of a perturbed distributive model lacking the new mechanism, shows both a population of abnormally high activation as well as a population of abnormally low activation compared to a mixed model (Fig. 6d). Thus, the change in processivity could be interpreted as an example of “reversible complex formation”, a network motif found at the core of mammalian and yeast stress response pathways implicated in conveying robustness^42^.

According to our modeling efforts, corroborated by pull down assays, this switch in processivity is in part due to increased binding of phosphorylated Hog1 to Pbs2, that we termed affinity feedback. Based on the congruency between model prediction and experimental data, we postulate that phosphorylation of S248 on Pbs2 by Hog1 constitutes a plausible mechanism by which the higher affinity is achieved. Available data, however, cannot exclude that lower diffusion rates caused by cell shrinkage may contribute to increased processivity^43,44^. Indeed, it has been argued that molecular crowding might affect processivity in HeLa cells^45^. However, since the possibility of tuning MAPK activation results in specific and emergent systems output, regulated switching between a distributive and processive phosphorylation mechanism is best explained by an active feedback mechanism.

In addition to affinity feedback, our modeling approach also predicts that a second, catalytic feedback mechanism drastically increases the reaction rate. Interestingly, recent experiments confirm osmostress-mediated enhancement of the reaction between Pbs2 and Hog1^46^, and suggest that a new downstream osmosensor could fulfill the role of positive feedback. However, further work will be required to experimentally validate catalytic feedback mechanisms regulating HOG signaling.

Our findings on the processivity of Hog1 phosphorylation may have important implications to explain dynamic properties of MAPK pathways in mammalian systems. For example, the mammalian Hog1 homolog p38 was recently shown to be phosphorylated in a semi-processive manner^47^, which may be best explained by a mixed activation model. Likewise, mammalian ERK shows context-specific differences in its ratio of single and double phosphorylated species^48^. In even broader context, other switch-like transitions such as cell cycle progression or cell differentiation use an increasing number of phospho-sites of effector proteins as a strategy to alleviate the need for a cascade. For example, degradation of the *S. cerevisiae* cyclin-dependent kinase inhibitor Sic1 requires phosphorylation of at least six residues to allow S phase entry^49^. It has been argued that a distributive mechanism to phosphorylate these sites results in a highly ultrasensitive, switch-like response. Sic1 is phosphorylated by two distinct kinase complexes, Cln2-Cdk1 and Clb5-Cdk1, which bind and first phosphorylate priming phospho-sites on Sic1, which in turn triggers processive phosphorylation of the entire phospho-degron^11^. This systems behavior could thus be viewed as an extreme case of a mixed phosphorylation mechanism, switching from a distributive to a processive mode of activation. Theoretical analyses predicted that increasing the number of phosphorylation sites sharpens the threshold, but might only allow for a graded increase beyond the threshold^50^. However, a theoretical framework with a mixed mechanisms confirms its potential for additional behavior already in a system with three phosphorylation sites^51^. Indeed, experimental evidence for a mixed mechanism termed semi-processive phosphorylation was described for the multisite phosphorylation of Pho4^10^. It is thus tempting to speculate that the here described tunable phosphorylation mechanism in combination with regulatory feedback could be utilized not only to activate MAPK’s but may more generally apply to many multisite phosphorylation systems, as it confers increased robustness and the ability to finetune ultrasensitive signaling dynamics such as efficient switching and threshold adaption.

## Methods

### Yeast strain construction

Yeast strains and plasmids are listed in Supplementary Tables S3 and S4. For strains used in live cell imaging, yMU49 containing the nuclear HTA2-CFP marker was used as a starting strain. HOG1-YFP was amplified by PCR from yMU19, and a simple transformation protocol using lithium acetate, polyethylene glycol and heat shock induced transformation was utilized to create yMM001. The SKARS sensor^52^ or dPSTR^53^ were transformed into yMM001 after cutting of plasmids pED45 or pDA183 with corresponding restriction enzymes to create yMM003 or yMM004 respectively. The Pbs2 deleted strain was created by transformation of the PCR amplification product of the NAT cassette from pSP135 with primers containing sequences 1000 bp up- or downstream of the gene of interest. Successful plasmid cut and PCR product length were confirmed by gel electrophoresis.

Strains for in vitro kinase assays were constructed as follows. The wild type allele of Hog1 was cloned into p426TEG1 (PTEF1-GST, URA31, 2µ) to yield pRS426 GST-Hog1 (pEN133)^54^. Catalytically inactive GST-Pbs2 K/M (Lys389 to Met^55^) (pGY75), was cloned into pGEX46P1 by EcoRI-XhoI. GST-Pbs2 K/M^S248A^ (PBS2 with Ser248 to Ala mutation) (pGY77) were generated by site directed mutagenesis and verified by sanger sequencing. As a control, GST-Sic1 was also purified from *E. coli* as previously described^56^. Plasmids were incorporated into a *pbs2Δ* strain (yHS206) via homologous recombination.

For strains used in western blot experiments, Pbs2 mutations were introduced with a pop-in pop-out approach from the original YMN455 (HOG1-6HA::HIS PBS2::URA), the different Pbs2 alleles were PCR amplified from (pGY86,87, 88 for WT, Pbs2S248A and Pbs2S248E, respectively). Transformants were selected by FOA counterselection. Point mutations for YMN457 (Pbs2^S248E^), YMN459 (Pbs2^S248A^) and YMN460 (Pbs2^WT^) were verified by Sanger sequencing.

Strains used for Pbs2 interaction experiments were derived from YMN455, 457, 459 and 460 by PCR mediate integration of KAN resistance cassette to the HOG1 locus. The resulting strains are YMN551 (BY4741 *PBS2::URA HOG1::KAN*), YMN552 (BY4741 *HOG1::KAN* Pbs2^S248E^), YMN552 (BY4741 *HOG1::KAN* Pbs2^S248A^), and YMN553 (BY4741 HOG1::KAN Pbs2^WT^).

### Live cell imaging

For live cell imaging experiments, yeast cells were grown in Synthetic Defined (SD) media with 2% glucose. Exponentially-growing cells were transferred to a microfluidic device (Y04C, Merck Millipore) or 96 well plate and live cell imaging was carried out at 30 °C using a fully-automated inverted epi-fluorescence microscope (Ti-Eclipse, Nikon) in an incubation chamber. Osmotic stress was induced with the pressure controller (ONIX, Merck Millipore) or by manual pipetting by exchanging the medium with media containing NaCl at the specified concentrations. Images were taken with a high numerical aperture oil immersion objective lens (CFI Plan Apo 60X, Nikon; N.A. = 1.4), and controlled using micro-manager. Each frame was imaged with relevant fluorescent set-up (CFP, YFP, mCherry and Cy5 fluorescent filters with LED illumination). Cell segmentation, tracking and feature extraction were done using the MATLAB-based YeastQuant software^57^, using Alexa 680 fluorescent dye for cell segmentation^57^. The CFP channel was used to define cytosolic and nuclear regions based on Hta2-CFP images, by defining a certain intensity threshold. Individual cells during time-lapse imaging were followed by tracking the nucleus. The cytosol and nucleus of individual cells was segmented and various properties including cell area and average intensity of fluorescent signals in the segmented objects were quantified.

### In vitro kinase assays

The GST fusion protein encoding Pbs2^K/M^ and Pbs2^K/M S248A^ (pGY75 and pGY77 respectively) were expressed in *E. coli* and purified using glutathione-Sepharose beads (GE Healthcare) and STET buffer (10 mM Tris pH 8.0, 100 mM NaCl, 1 mM EDTA pH 8.0, 5% Triton X-100, 2 mM dithiothreitol (DTT), 1 mM phenylmethylsulfonyl fluoride (PMSF), 1 mM benzamidine, 2 μg/ml leupeptin, 2 g/ml of pepstatin). Active GST-Hog1 was purified from NaCl treated (0.4 M NaCl for 10 min) wild-type cells harboring a multicopy vector (pEN133, pRS426 TEG1-Hog1).

For kinase assays, 0.5 μg of GST-Hog1 was incubated with 0.25 μg of Pbs2^K/M^ or Pbs2^K/M S248A^ in the presence of kinase buffer (50 mM Tris–HCl pH 7.5, 10 mM MgCl_2_, 2 mM DTT) and 50 μM ATP together with [γ-^32^P]ATP (0.1 μCi/μl) and incubated for 15 min at 30 °C. The reaction was terminated by the addition of 5 × SDS loading buffer. Labeled proteins were resolved by SDS-PAGE, stained, dried and detected by autoradiography with Fujifilm BAS-5000 phosphoimager.

### Western blot measurements

Yeast cells were grown to mid-exponential log phase (OD660 = 0.7) before being subjected to osmostress (0.4 M NaCl) for the indicated times. Samples were fixed with 20% tricholoracetic acid, resolved with SDS-polyacrylamide gel electrophoresis and proteins transferred to PVDF membranes (Immobilon FL, IPFL85R). Total or phosphorylated Hog1 was detected using either primary antibody for phospho-Hog1 (Cell Signaling, 9215 S) or total Hog1 (Santa Cruz, SC-165978) with a final concentration of 0.2 μg/ml and visualized by secondary antibodies for mouse (LI-COR 926-32212) and rabbit (LI-COR, 926-68073). Incubations were done in PBS-BSA 5% (Sigma, A7906). Blocking and antibodies were diluted using Intercept blocking buffer (LI-COR, 927-60001) and fluorescence was detected with a LI-COR Odyssey Infrared Imaging System 9120. Western blot quantification was done using Image J and a paired t-Test (Paired Two Sample for Means was calculated).

### Pull down assays

Hog1 ORF was cloned into pSECT vector with BamHI (pEN111) and transformed into BL21. Expressed Hog1-HIS was purified using 500 μl of Complete His-Tag purification resin (Roche, 5893682001) for each 500 ml of *E. coli* pellet, following the manufacturer’s protocol. An overnight preculture (25 ml LB + Chloramphenicol 50 µg/ml) was inoculated into 1 L of LB for 2 h at 37 °C before addition of IPTG (1 mM) for 8 h at 20 °C. Cells were pelleted by centrifugation (5 min 5200 *g*) and pellets were stored at −80 °C. Yeast extracts were obtained by growing *hog1*::KAN Pbs2 (WT, S248A and S248E or *pbs2*::URA) until mid-exponential phase before applying mild osmotic stress (0.4 M NaCl). Cell lysates were obtained by thawing cells in sonication buffer (50 mM Sodium Phosphate, 30 mM NaCl and 0.5% NP-40) with protease inhibitor (PMSF 1 mM, Benzamidine 1 mM, and Leupeptin 2 µg/ml).

Purified bead-bound Hog1 HIS was evenly split into columns (Mo Bi Tec, M1002S) and incubated for 2 h at 4 °C with 5 mg of total protein obtained from NaCl-stressed yeast extracts diluted in kinase buffer (50 mM TrisHCL pH 7.5, 10 mM MgCl_2,_ ATP 50 µM). Samples were washed twice in the columns (1 min 80 *g*) with 500 µl of sonication buffer 100 mM NaCl and sonication buffer 200 mM. Finally, beads were resuspended in sample buffer and boiled before loading onto 10% SDS PAGE. Detection of Pbs2 was done by incubating the membranes with anti-Pbs2 antibody (Santa Cruz, sc-6813) with a final concentration of 2 µg/ml and total Hog1 (Santa Cruz, sc-165978) with a final concentration of 0.2 μg/ml. Incubations were done in PBS-BSA 5% (Sigma, A7906). Images from chemiluminescence were obtained using Claity ECL (Bio-Rad, 1705061) and a chemiluminescence program (Odyssey Fc Imager). Image quantifications were done with Image J (Fiji).

### Data sources and incorporation into model definition

The experimental data were collected from multiple sources. If not otherwise noted the BY4741 yeast strain exposed to 0.4 M NaCl stress was used. Importantly mass spectrometry measurements analyzing long term^29^ and very short-term changes in phospho-site abundance were included. Since the relative phospho-site change 60 s after NaCl addition varies slightly between these two studies, the mean of both values was taken for this time point.

To determine the degree of Hog1 phosphorylation, the maximal values determined by western blot measurements were used^26^. Data for Hog1 phosphorylation upon inhibition of Hog1 with or without addition of salt were taken from the same experimental set. Even though the authors used a different strain with a different perturbation agent (KCl instead of NaCl) the qualitative dynamics of the Hog1 response were essentially identical^34^. Moreover, the switch-like response resulting in full Hog1 phosphorylation for all salt concentrations above a certain, low threshold justified inclusion of these data.

Additional western blot measurements were utilized for different phosphatase mutants. Compared to mass spectrometry measurements, quantification of western blots is complex and these results often reveal more qualitative than quantitative results^58^. For example, different antibodies are known to have different binding properties^59^. As a consequence, measurements of Hog1 phosphorylation in Ptp2/Ptp3 mutants in the literature revealed quite varying results^19–21,26,60^. However, despite these differences, the data largely agree that deletion of Ptp2 results in increased basal levels and prolonged Hog1 phosphorylation, while deletion of Ptp3 shows only minute differences compared to wild-type controls. To account for this variability, we utilized the data from ref. ^20^, but allowed the parameters for the error model of these measurements to be sufficiently big and independent from other western blot data sets. Furthermore, as the antibody used detects phosphorylated tyrosine, the corresponding output species of the model was set to be Hog1 doubly phosphorylated or mono-phosphorylated at tyrosine 176.

Microscopy measurements of Hog1 relocation to the nucleus report on Hog1 activity, and such data was used to quantify the response of single branch and phospho-site mutants. Similarly, microscopy-based measurements of cell size were used to fit the volume module. Information on Hog1 activation at 0.2 M NaCl stimulation with varying frequency (2, 4, 8, 16 min) were extracted from ref. ^61^. Volume and Hog1 relocation measurements in cells deleted for *SLN1* or *SHO1* were taken from ref. ^62^. Although the authors used sorbitol instead of NaCl for their experiments, own measurements confirmed that the response of cells to 0.6 M sorbitol is quantitatively and qualitatively nearly identical to perturbations with 0.4 M NaCl. To assess negative feedback of Hog1 on Ssk2, we used published Hog1 relocation measurements^35^. Due to the fact that non-phosphorylatable Ssk2 mutant exhibit reduced cell shrinkage and thus a lower maximal Hog1 nuclear to cytosolic ratio, only data from later time points were included, which correct for the increased time it takes for the Hog1 ratio to reach basal levels. Data for Gpd1 expression changes were selected from ref. ^63^, and information about Ptp2 and Ptp3 mRNA levels were extracted from ref. ^19^. Western blot measurements of total Hog1 phosphorylation upon inhibition with small-molecule inhibitors in wild type and *ssk2Δ* strains were taken from refs. ^36^ for basal activity and^26^ for activity upon increase of osmolarity. As most antibodies used to measure Hog1 phosphorylation bind to both the doubly and mono-phosphorylated species^26^, these western blot measurements were considered as total amount of phosphorylated Hog1 irrespective of phosphorylation grade.

The observables in these data were incorporated as model variables by defining observables that correspond to the experimentally measured quantity. We defined the relative ratio between the current absolute number of a protein species and its absolute number at the very start, the phosphorylated percentage as the absolute number of phosphorylated protein divided by the total number of the protein, and the concentration ratio of Hog1 in the nucleus compared to the cytosol, for the mass spectrometry, western blot, and fluorescence ratio data sets respectively. Further details on how the observables and their corresponding data set were encoded in the model can be gathered from Supp Table S6: Observables.

### Modeling

All modeling was performed using the Data2Dynamics modeling environment^64^ and computation was carried out on the Euler cluster provided by ETH Zurich accessed via BASH scripts. Parameter optimization was carried out by multistart followed by a deterministic trust region algorithm. The goodness of fit was evaluated by Goodness-of-fit = −2* log(Likelihood). For parameter optimization, parameters were considered on a log-scale. In a first step, 10,000 starting parameter vectors were created. Sampling was done uniformly between the lower and upper boundaries of each parameter. Lower and upper boundaries were first set to be minus three to three, respectively, spanning six orders of magnitude. After successful parameter estimation (after either 400 iterations or if the value of the objective function changes by less than 1e-6) the 100 best-fitting parameter vectors were taken and their correlation coefficient determined. 10,000 starting vectors for the next optimization run were generated by sampling from a multivariate normal distribution whose parameters were determined by the 100 best fitting parameter vectors of the previous run. This procedure was repeated until the best goodness of fit of the latest run turned out as good or worse than the run before.

To minimize the risk of the optimization procedure redundantly reproducing solution clusters with very similar values, we included additional steps, such as optimization of crucial model topologies being redone up to eight times with different starting parameters, and ensuring that the relative ordering of the fits was consistent over various conditions, such as leaving out of data points, or changing of parameter boundaries. In general, parameter identifiability was not a major concern, as some model topologies were not able to recapitulate the data and we were primarily interested in the relative quality difference of each topology prediction. The size of the model and its complicated nature also impeded identifiability analysis by profile likelihoods. Instead, we simulated species concentration from a model with known parameters. From these simulations, we sampled data points equivalent in number and time points to the experimental dataset, and performed the parameter optimization procedure described above. All but twelve of the 82 newly fitted parameters were within one order of magnitude from the parameters used to generate the data, with a median of 0.11.

In all our models, certain parameters of the Sho1 sub-branch (Sho1 binding to Ste11, phosphorylation of Pbs2 by Sho1 and dissociation of activated Pbs2 from Sho1) assumed values much higher than expected (up to 10^6) after parameter optimization. We speculate that this reflects a stable complex that forms at the cell membrane which incorporates all involved species (Sho1, Ste11, Ste50, Ste20, the scaffolding protein Ahk1, and more) and brings them into close proximity so that reactions occur faster than expected by simple diffusion^65–67^. As this was observed with all model topologies, we feel confident to compare the different topologies relative to one another even if the models do not fully capture the Sho1 sub-branch. Similarly, we did not incorporate the latest information that describes how Ste11 only phosphorylates one phospho-site on Pbs2^46^.

Comparison of different models was done using the AIC.

1 $${{{{{\rm{AIC}}}}}}=2k-2{{{{{\rm{ln}}}}}}({L}_{\max })$$

With “*k*” being the number of fitted parameters and *L*_*max*_ the maximal likelihood.

Detailed information on the best fitting mixed model can be found in Supp Tables S6–S10.

### Processivity score

We introduced a score (2) to quantify the change in processivity of the Hog1-Pbs2 reaction over the course of the response.

2 $$\frac{[{{{{{\rm{Pbs}}}}}}2{{{{{\rm{PPHog}}}}}}1{{{{{\rm{P}}}}}}]*{k}_{{phospho}}+[{{{{{\rm{Pbs}}}}}}2{{{{{\rm{PPHog}}}}}}1{{{{{{\rm{P}}}}}}}_{{{{{{\rm{feedback}}}}}}}]*{k}_{{phosph}{o}_{{feed}}}}{[{{{{{\rm{Pbs}}}}}}2{{{{{\rm{PPHog}}}}}}1{{{{{\rm{P}}}}}}]*{k}_{{off}}+[{{{{{\rm{Pbs}}}}}}2{{{{{\rm{PPHog}}}}}}1{{{{{{\rm{P}}}}}}}_{{{{{{\rm{feedback}}}}}}}]*{k}_{{off}}}$$

It is defined as the probability of a monophosphorylated Hog1 bound to activated Pbs2 undergoing a second phosphorylation step against the probability of the proteins dissociating. Feedback on Pbs2 is reflected in a higher rate of phosphorylation of Hog1 while the dissociation constant is kept constant.

### Modeling distributive or processive MAPK phosphorylation mechanisms in a basic three-tiered MAPK cascade

We utilized the paradigm of Huang and Ferrell, which models the basic three-tiered MAPK cascade. We collected or generated data measuring activation of MAP kinases from both yeast and mammalian cells that display different degrees of ultrasensitivity quantified by their Hill-coefficient. We found that Hog1 activation in *S. cerevisiae* by NaCl stimulation occurs with a hill-coefficient of 3.3, while its mammalian counterpart p38 in HeLa cells is stimulated by anisomycin with a hill-coefficient of 3^45^. In comparison, the highly ultrasensitive activation of p38 via sorbitol in *Xenopus* oocytes occurs with a hill-coefficient of 14.4^13^, and the graded response of Erk2 in HeLa cells stimulated by EGF with a hill-coefficient of 1.2^45^.

The parameters of the basic model were optimized according to our described protocol and the best overall fit determined according to log-likelihood criteria. This was done for a distributive and a processive topology of MAPK activation. For Hog1 and both p38 data sets it was not possible to distinguish between a purely distributive or purely processive mechanism. Both models were able to fit the data equally well (difference in AIC was smaller than ten) and the utilized parameters still range within biologically feasible boundaries (Supp Fig. S1C)^13,68^. In contrast, the graded activation of Erk2 showed a clear preference for processive phosphorylation with significantly better fitting results, consistent with experimental evidence^45^. Experimental measurements also corroborate the possibility that mild ultrasensitive behavior can be achieved in nature using purely processive mechanisms. Thus, a processive mechanism with the correct parameters is also able to recapitulate even highly ultrasensitive behavior and thus cannot be readily discarded.

### Statistical analysis

All data were analyzed and visualized using MATLAB software. Error bars in histograms denote the standard deviation of the proportion of measurements within specific bin limits computed from 200 resamples. A MATLAB implementation of Wilcox quantile shift function was used to quantify and plot the difference between different distributions. The implementation computes quantiles using Harrel-Davis quantile estimator, performs 200 resamples for a bootstrap estimation of 95% confidence intervals and controls for multiple testing^69^. Multiple linear regression to determine whether a linear relationship between concentration changes of different proteins and maximal Hog1PP percentage exists was performed using built-in MATLAB functions.

### Reporting summary

Further information on research design is available in the Nature Portfolio Reporting Summary linked to this article.

## Supplementary information

Supplementary information

Reporting Summary

## Data Availability

Source data are provided in this paper. The fluorescence microscopy data generated in this study have been deposited in the ETH Research Collection database under the title “Positive feedback induces switch between distributive and processive phosphorylation of Hog1” (10.3929/ethz-b-000597634). Source data are provided with this paper.

## Source data

Source data file

## Code availability

The models and best-fitting parameter values generated in this study have been deposited in the BioModels database under accession code MODEL2206230001.
