# Supplementary material for: Positive feedback induces switch between distributive and processive phosphorylation of Hog1
Source: Nat Commun. 2023 Apr 29;14:2477. doi: 10.1038/s41467-023-37430-y (PMC10148820; doi:10.1038/s41467-023-37430-y)
Supplement: Supplementary file 1 — Supplementary information [file 41467_2023_37430_MOESM1_ESM.docx]

**Supplementary information**

**Positive feedback induces switch between distributive and processive phosphorylation of Hog1**

Maximilian Mosbacher^1, 2^, Sung Sik Lee^1, 3^, Gilad Yaakov^4, 5^, Mariona Nadal-Ribelles^4, 6^, Eulàlia de Nadal^4, 6^, Frank van Drogen^1^, Francesc Posas^4, 6^, Matthias Peter^1, #^ and Manfred Claassen^2, 7, #^

^1^Department of Biology, Institute of Biochemistry, ETH Zurich, Zurich, Switzerland

^2^Department of Biology, Institute of Molecular Systems Biology, ETH Zurich, Zurich, Switzerland

^3^ Scientific Center for Optical and Electron Microscopy, ETH Zurich, Zurich, Switzerland

^4^Department of Medicine and Life Sciences (MELIS), Universitat Pompeu Fabra (UPF), 08003, Barcelona, Spain

^5^Current address/affiliation: Department of Molecular Genetics, Weizmann Institute of Science, Rehovot, Israel

^6^Institute for Research in Biomedicine (IRB Barcelona), The Barcelona Institute of Science and Technology, [Baldiri Reixac, 10, 08028 Barcelona, Spain](https://maps.google.com/?q=Baldiri+Reixac,+10,+08028+Barcelona,+Spain&entry=gmail&source=g)

^7^ Current address/affiliation: Department of Internal Medicine I, University Hospital Tübingen, Faculty of Medicine, University of Tübingen, Germany

# corresponding authors

manfred.claassen@med.uni-tuebingen.de

matthias.peter@bc.biol.ethz.ch

**Overview of modelling approach**

We describe the HOG response mathematically in terms of biochemical reactions. Specifically, we used ordinary differential equations (ODEs) based on mass action kinetics. The following reaction illustrates how the reaction formulation translates to an ordinary differential equation for one of the three reactants.

$$\begin{aligned} A+B\to C \frac{d\left[ C \right]}{dt}=k*\left[ A \right]*\left[ B \right]\# \end{aligned}$$

( 1 )

In the hypothetical reaction compound A and compound B bind to form compound C, the change of concentration of C ($\left[ C \right]$) is determined by the product of rate constant k and concentration of A and B ($\left[ A \right]$, $\left[ B \right]$).

Box1 shows the more complicated reaction topology of the mixed phosphorylation mechanism of Hog1 by Pbs2 reported in this study. It can be written as a set of reactions and their corresponding mass action kinetics ODEs. Supp table S9 lists the corresponding list of reactions and ODEs for the competing reaction topologies assessed in this study, i.e. the processive and distributive phosphorylation mechanisms.

**
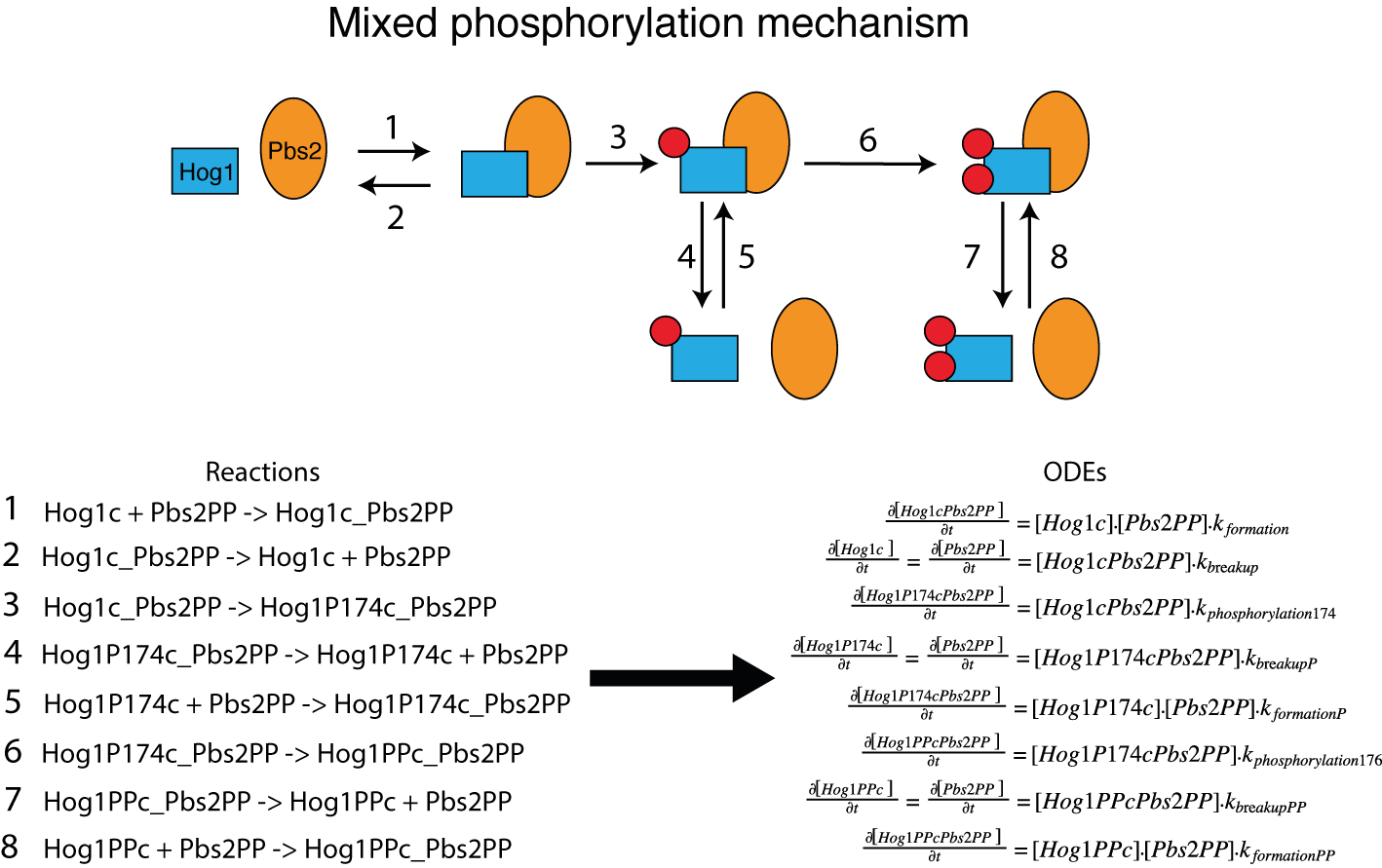
**

**Box 1: Conversion of a graphical illustration of the mixed phosphorylation mechanism into a series of ODEs.** The eight reactions implementing the mixed phosphorylation mechanism are displayed in a graphical scheme. This mechanism is shown as a set of equivalent reaction formulas and converted into a set of mass action kinetics ODEs. For the sake of clarity reactions dealing with the second possible mono-phosphorylation of Hog1P176 have been omitted.

As no experimental values are available for most of the rate constants and initial concentrations, these parameters need to be estimated from experimental data. Such procedures are well established and are based on comparing the experimental data with the data that we expect to see for a specific parametrization of the model via simulation (ODE integration). Parameter estimation is based on evaluating many different parametrizations and seeking to minimize the difference between experimental and the simulated data by adjusting the parameters. In this study, we quantify this difference by means of a likelihood function that assesses this difference with respect to the expected differences due to measurement noise. If we assume that the measurement noise of each data point is normally distributed, we arrive at the following function.

$$\begin{aligned} L\left( \theta| Y;\sigma\right)=\prod_{i=1}^{n} f\left( Y_{i} | \theta;\sigma\right)=\prod_{i=1}^{n} \frac{1}{\sqrt{2\pi\sigma^{2}}}\exp\left( -\frac{\left( Y_{i}-\hat{Y}_{i}\left( \theta\right) \right)^{2}}{2\sigma^{2}} \right) \# \end{aligned}$$

( 2 )

Thus, the likelihood is determined by the product of the squared difference of data point $Y_{i}$ and the simulated value $\hat{Y}_{i}$, which depends on model parameters $\theta$. This can be read simply as the sum of squares (SSQ), also known as the residual sum of squares (RSS), weighted by $\frac{1}{2\sigma^{2}}$ with $\sigma$ being the standard deviation of the experimental data.

$$\begin{aligned} L\left( \theta| Y;\sigma\right)= \frac{n}{\sqrt{2\pi\sigma^{2}}}\exp\left( -\frac{1}{2\sigma^{2}}\mathrm{SSQ}\left( \theta;Y \right) \right) \# \end{aligned}$$

( 3 )

$\sigma$ was either estimated directly from the experimental data or in cases where such was not possible, e.g. mass spectrometry measurements of single phosphorylated Hog1^8^, treated as an additional parameter to be estimated.

The implementation of the likelihood function of the full ODE system was performed by the Data2Dynamics modelling environment that also allows for parameter optimization with respect to the likelihood. For optimization we utilized a deterministic trust region algorithm that is part of the D2D package. We utilized the algorithm in a multi-start approach in which 10000 different starting parameter vectors were randomly chosen and local optimization performed on each one. Each individual parameter value of these starting vectors was sampled uniformly between a minimum and maximum value (10^-3^ and 10^3^) and independent of other variables. These boundaries were chosen to sample a sufficiently big range while reflecting the distribution of enzymatic reaction rates derived from *in vitro* experimental k_cat_ values^1^. This step resulted in 10000 optimized parameter vectors.

In a second step, we computed the correlation coefficient of the 100 best fitting parameter vectors. The best fitting parameter vector and this correlation coefficient was then used as the mean vector and covariance matrix of a multivariate normal distribution from which 10000 new starting parameters were generated. A graphical summary of this cyclical optimization process is provided in Box2.


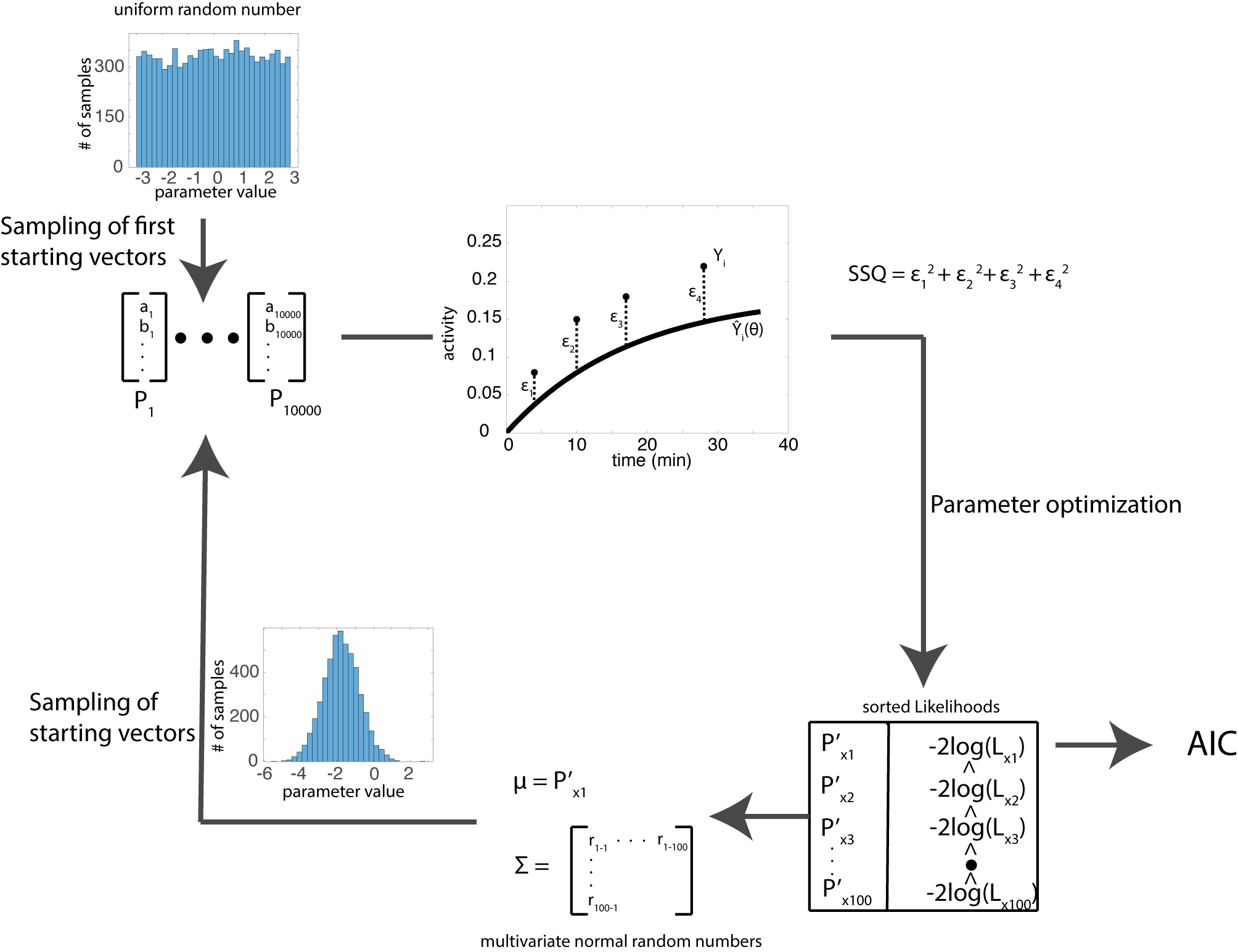


**Box 2: Schematic of the parameter optimization cycle**. Sampling from a uniform distribution generates the initial starting vectors. Parameter optimization is done by minimizing the SSQ, the sum of the squared residuals ε_i._ The 100 best optimized parameter vectors P’, as determined by their negative log-likelihood, and used to sample further similar starting vectors for a second round of refined parameter estimation. Comparison with other reaction topologies is performed via the Akaike information criterion (AIC), that constitutes a score that trades off the log-Likelihood and the complexity, i.e. the number of parameters of a reaction topology.

Comparison of models of different topology was done using the Akaike information criterion which penalizes the goodness of fit as given by the likelihood, by adding the number of parameters used in a model.

$$\mathrm{AIC}=2k-2\ln\left( L_{max} \right)$$

( 4 )

This is necessary, as some topologies have more parameters than others, thus being more likely to overfit.

**Volume Sub-model**

The volume sub module is implemented according to an established volume regulation model, the principles of which were first described in Klipp et al, 2005^2^ and later used in other modelling studies including Zi et al, 2010^3^.

Briefly, the flow of water in and out of a cell and thus the change of cell volume is modelled by equation 5.

$$\frac{d\left[ V_{os} \right]}{dt}=-G*Lp*\left( {PI}_{e}+{PI}_{t}-{PI}_{i} \right)$$

( 5 )

The change in volume (V_os_) is proportional to the osmotic pressure difference and the hydraulic membrane permeability $Lp$ and a geometrical factor $G$. In our model these two constants have been unified in a single constant, either G_parameter cytosol_ and G_parameter nucleus_ for the cytosolic and nuclear compartment respectively. The osmotic pressure difference itself is the difference between external osmotic pressure ($\mathrm{PI}_{e})$ minus internal osmotic pressure ($\mathrm{PI}_{i}$) plus the turgor pressure ($\mathrm{PI}_{t})$. An increase in external osmotic pressure by addition of salt leads to a positive pressure difference and outflow of water which equals a reduction in cell volume and is also reflected in a drop in turgor pressure.

As detailed in Klipp et al, 2005^2^, the change of turgor pressure has been traditionally modelled as dependent on volume change $\Delta PI_{t}\sim\Delta V/V^{PI_{t}=0}$, a formula derived from empirical observation. A time dependent value of turgor pressure, $\mathrm{PI}_{t}(t)$, is then implemented in our model using equation 6.

$$\mathrm{PI}_{t}(t)=\frac{\mathrm{PI}_{t}^{0}*(V_{os}(t)-V_{os}^{{PI}_{t}=0})}{V_{os}^{0}-V_{os}^{{PI}_{t}=0}}$$

( 6 )

${PI}_{t}^{0}$ and $V_{os}^{0}$ represent turgor pressure and volume at time point zero respectively. It is further assumed that cells retain a certain volume when turgor pressure drops to zero, $V_{os}^{{PI}_{t}=0}$. Importantly, this equation implies that turgor pressure is linearly dependent on the changing volume ($V_{os}(t)$).

External osmotic pressure is modelled by equation 7 and mainly depends on external salt concentration.

$$\mathrm{PI}_{e}=\mathrm{PI}_{e}^{0}+w*\left[ \mathrm{NaCl} \right]$$

( 7 )

Finally, internal osmotic pressure can be modelled using equation 8 (Boyle-Van’t-Hoff relation) and depends on the number of osmotically active moles ($n$) in relation to the gas constant (*R*), temperature (*T*) and changing cell volume (*V_os_*).

$$\mathrm{PI}_{i}=\frac{n*R*T}{V_{os}}= \frac{n_{0}+n_{Glyc}}{V_{os}}*R*T= \frac{n_{0}+\left[ \mathrm{glyc} \right]*V_{cyt}}{V_{os}}*R*T$$

( 8 )

Yeast contains a high number of osmolytes such as amino acids, various ions or sugars. It has been shown that glycerol plays a preeminent role in adjusting osmotic pressure differences via rapid accumulation after high-osmolarity pressure that is dependent on HOG activity^4^. Therefore, osmolyte molarity was split into a basal term of non-changing osmolytes (n_0_) and the changing molarity of cytosolic glycerol ([glyc]). To adapt to high external osmolarity, PI_i_ needs to be increased.

As already mentioned, glycerol is the defining osmolyte in yeast and therefore its intracellular concentration needs to be increased to balance increased external osmotic pressure^5^. Equation 9 describes this change of intracellular glycerol concentration using three terms dealing with glycerol production and two further terms describing glycerol export.

$$\frac{d\left[ \mathrm{glycerol} \right]}{dt}=\left[ base\_line \right]*k_{glyc const production}+ \left[ Gpd1 \right]*k_{gpd1 production}+\frac{\left[ Hog1PPc \right]^{n hog1 glyc}*k_{glyc hog1 dependent production}*\mathrm{maciafactor}}{\mathrm{BET}A^{n hog1 glyc}+\left[ Hog1PPc \right]^{n hog1 glyc}}-\left[ \mathrm{glycerol} \right]*k_{glyc exp0}-\frac{\left[ \mathrm{glycerol} \right]*PI_{t}^{n for fps1}*k_{glyc\exp fps1}}{\mathrm{GAMM}A^{n for fps1}+PI_{t}^{n for fps1}}$$

( 9 )

Firstly, glycerol is produced at a constant basal rate (k_glyc const production_). A second term models the production dependent on the concentration of Gpd1 ([Gpd1]) and its corresponding production rate (k_gpd1 production_). Our model also includes a description of an increase of Gpd1 expression by activated Hog1PP and thus this term describes the delayed mechanism of increased glycerol production via enzymes involved in glycerol metabolism that are upregulated after salt exposure. The third term models the fast acting, direct regulation of metabolic enzymes by activated cytosolic Hog1 ([Hog1PPc]) that leads to increased glycerol production (k_glyc hog1 dependent production_).

The constant export of glycerol is modelled in term four. It is dependent on glycerol concentration ([glycerol]) and a constant export rate (k_glyc exp0_). The final part of equation 9 models the immediate closure of Fps1, a transmembrane channel mediating glycerol export, upon a drop of turgor pressure. That this rapid closure is indeed the first step of osmo-adaption and highly significant has been shown by Tamás et al, 1999^6^. It has also been described that activated Hog1 actively close Fps1 via its positive regulators Rgc1/2^7^. On the other hand, other studies show that Hog1 kinase activity might not affect glycerol leakage in the rapid response but rather at a later point of the adaption^8,9^. Therefore, we opted to tie Fps1 channel closure to turgor pressure to guarantee the immediate increase of glycerol that is observed.

The variable compartment size should be reflected in the concentration of the species that thus change relative to changes in volume. In our models this is being addressed by introducing separate reactions for each protein species that quantify the change in concentration due to change in compartment volume (for an example using the species Ste11 see equation 10).

$$\frac{d[Ste11]}{dt}=-\left[ Ste11 \right]*(-G_{parameter cytosol}*\frac{\left( PI_{t}+PI_{e}-PI_{i} \right)}{V_{cytosol}})$$

( 10 )

Remembering the definition of volume change in equation 5, $-G_{parameter cytosol}*\frac{\left( PI_{t}+PI_{e}-PI_{i} \right)}{V_{cytosol}}$ can be rewritten as $\frac{d\left[ V_{os cytosol} \right]}{dt*V_{os cytosol}}$, the relative change of cytosolic volume in a certain time period.

To illustrate how these additional reactions are integrated, we want to show the example of how the termolecular and reversible reaction of two molecules of Ste11 binding to Ste50 is implemented in our models (equation 11).

$$\frac{d\left[ Ste11 \right]}{dt}=k_{1}*\left[ Ste11Ste50 \right]-k_{2}*\left[ Ste11 \right]^{2}\left[ Ste50 \right]$$

$$-\left[ Ste11 \right]*(-G*\frac{\left( PI_{t}+PI_{e}-PI_{i} \right)}{V_{cytosol}})$$

( 11 )

Equation 11 contains all reactions in which [Ste11] is implicated. Our modelling framework assembles a corresponding ODE for each protein species. Importantly, they all contain a term accounting for concentration changes due to volume changes.

**
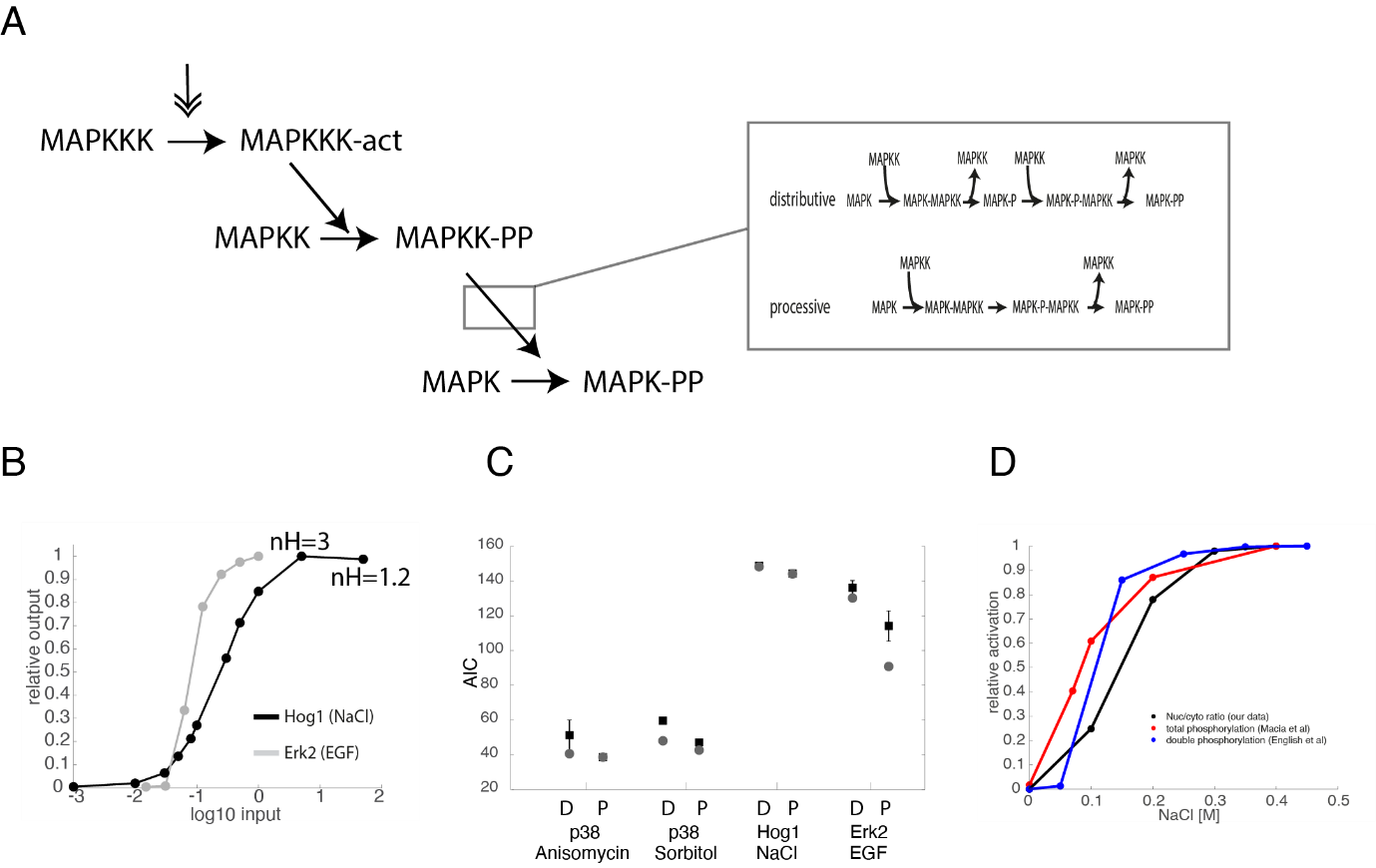
**

**Supp Figure S1: Modelling distributive or processive MAPK phosphorylation mechanisms**

(A) A schematic of the general MAPK model by ^10^. The two possible phosphorylation mechanisms, distributive or processive, are displayed. (B) Fitting a Hill function to data points of the activation of Hog1 by NaCl or Erk2 by EGF quantifies an ultrasensitive response of Hog1 activated by NaCl with a Hill coefficient of 3, and a graded response of Erk2 activated by EGF with a Hill coefficient 1.2. (C) A graph plotting the Akaike information Criterion (AIC) of the general MAPK model with either distributive (D) or processive (P) MAPK phosphorylation mechanism to different data sets. Median of the best 30 fitting runs and corresponding interquartile range is displayed by a black square and the single best fit via a grey circle. (D) Hill curves fit to relative Hog1 activation at different NaCl concentrations measured as Nuclear to cytosolic Hog1 ratio (black, nH=3.3), total Hog1 phosphorylation measured by antibody^11^ (red, nH=2.2) and phos-tag SDS-PAGE measurements of double phosphorylated Hog1^12^ (blue, nH=5.3).


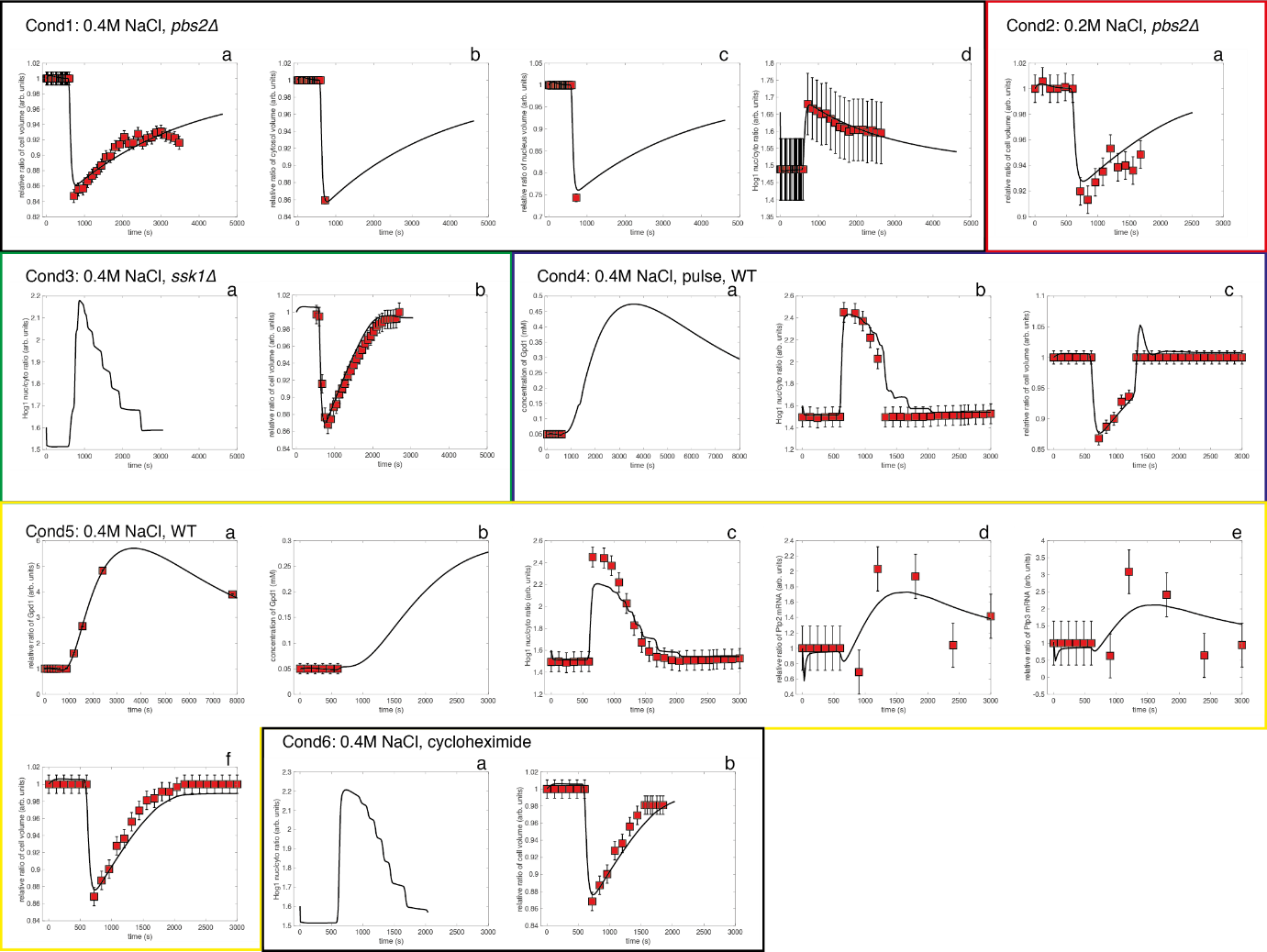


**Supp Figure S2: Data used for parameterization of the volume sub-model**

Data used for parameter optimization of the volume sub-model is shown as red squares. The simulation of the resulting best fit is indicated (solid black line). A total of six different conditions (boxes labeled Cond1-6), were fit simultaneously (see Supp Table S1 for details of data).

**
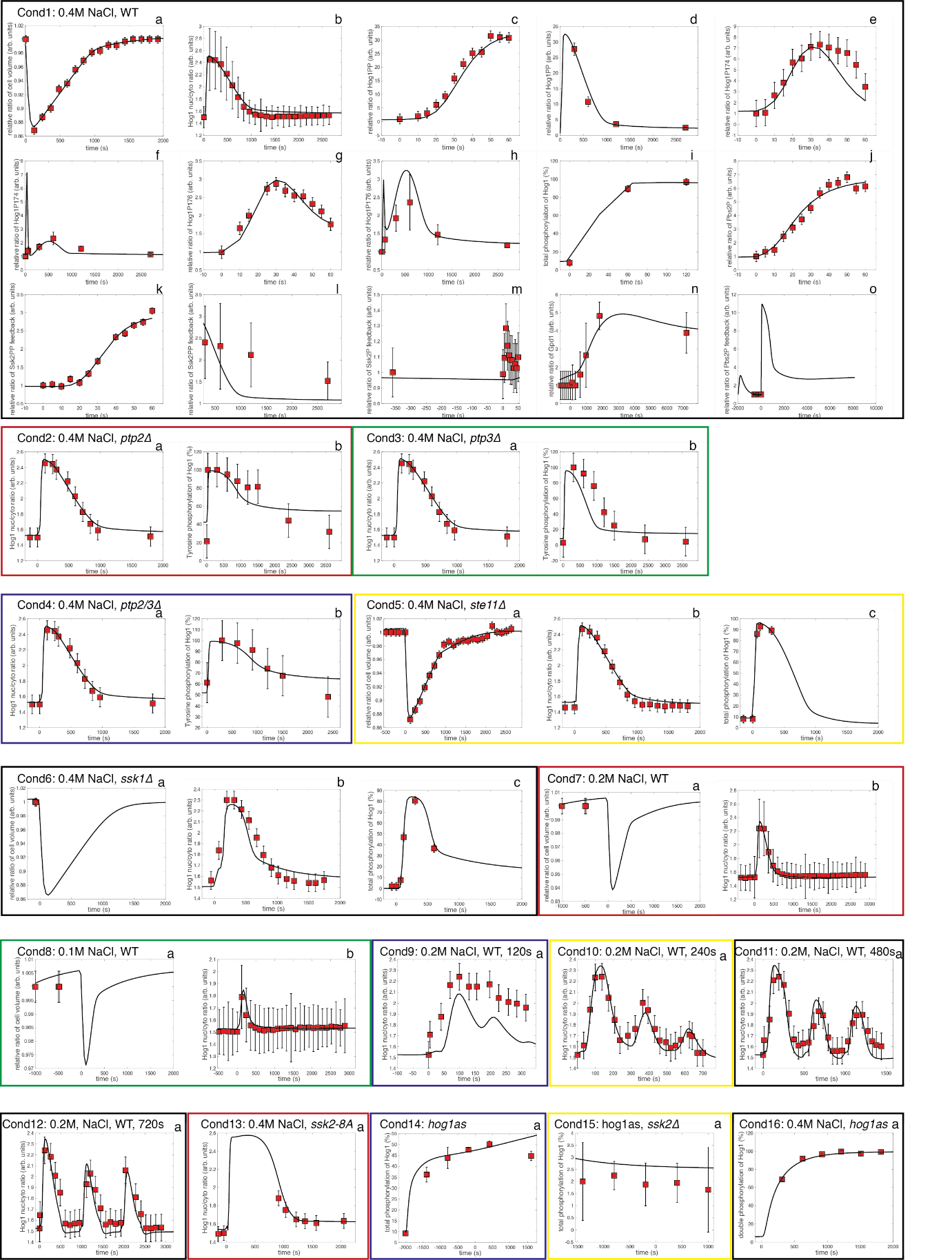
**

**Supp Figure S3: Data used for the parameterization of the overcomplete model**

Data points used for parameter optimization of the best fitting model as described in Figure 3B are shown as red squares. Note, that this data includes experimental measurements of monophosphorylated Hog1 species that were not previously used to generate the refined model (Figure 2B) that was used to distinguish between purely distributive and processive phosphorylation mechanisms and the presence or absence of feedback mechanisms. Thus, the best fitting model includes positive feedback on Pbs2, negative feedback, and a mixed phosphorylation mechanism as described in Figure 3A. Simulation of this model are indicated by the solid black line. The 16 conditions (boxes labeled Cond1-16) were fit simultaneously (see Supp Table S2 for details on data).

**
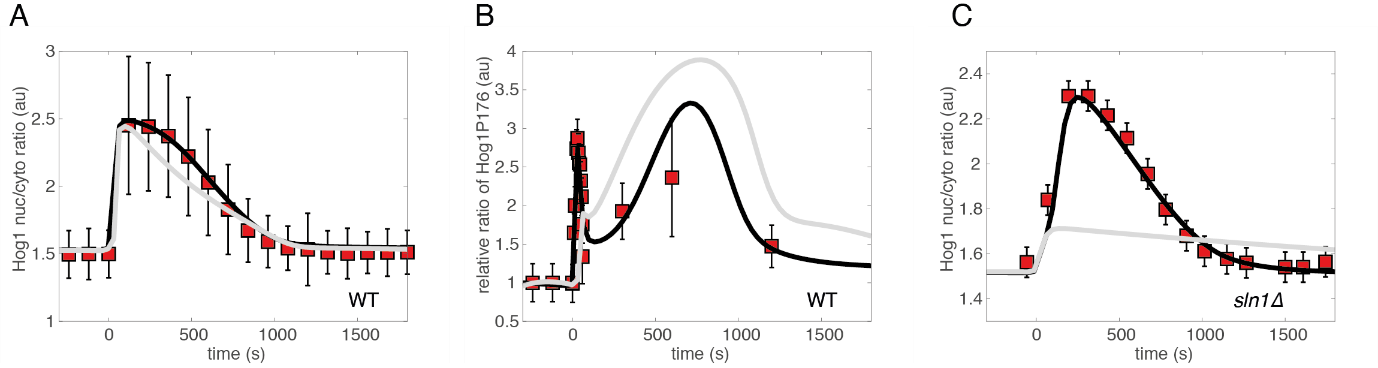
**

**Supp Figure S4: Differences between the best fitting results of the constrained and unconstrained processive model**

Data points used for parameter optimization of the full model are shown as red squares. Simulations of the resulting best fitting model from a topology with a processive phosphorylation mechanism and both negative feedback and positive feedback on Pbs2 are indicated for unconstrained (solid black line) and constrained (solid light grey) parameters. (A) Time courses of Hog1 nuclear to cytosolic ratio upon addition of 0.4M NaCl in wild type (WT) cells. Data are presented as mean values +/- standard deviation of n=1156 cells examined over 3 independent experiments (B) Time courses of the relative ratio between stimulated and basal levels of monophosphorylated Hog1-P176 upon addition of 0.4M NaCl in WT cells. Data for the first 60s by Kanshin et al are presented as values of peptide fold change +/- computational estimate of SEM and data by Vaga et al are presented as mean values of peptide fold change +/- standard deviation of n=3 independent experiments.^19,20^ (C) Time courses of Hog1 nuclear to cytosolic ratio upon addition of 0.4M NaCl in a *sln1Δ* strain. Data by Granados et al are presented as mean values +/- computational estimate of SEM with n=123 cells examined over 1 independent experiment.^13^

**
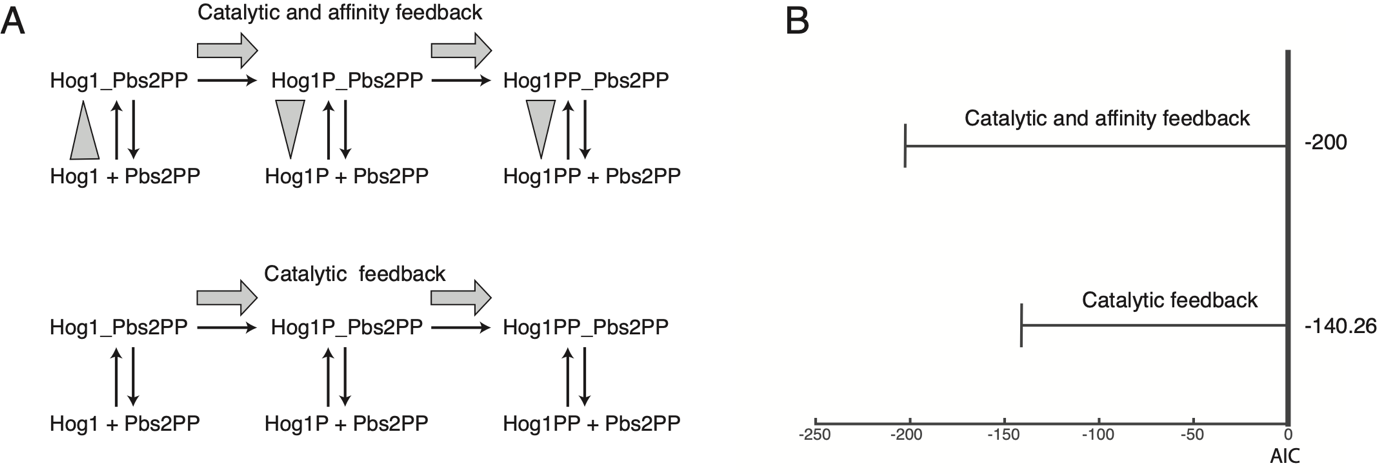
Supp Figure S5: Two component feedback mechanism leads to significantly better fitting results compared to a single component catalytic feedback model**

(A) Mechanisms of the two-component feedback and the single component catalytic feedback models. Grey arrows indicate increase in kinetic rate constants upon feedback. Grey triangles indicate a shift in the association/dissociation equilibrium towards the base of the triangle. (B) Akaike information criterion (AIC) was used to order optimized models with either two component feedback or single component catalytic feedback mechanism. A two-component model with catalytic and affinity feedback result in a significantly better fit.

**
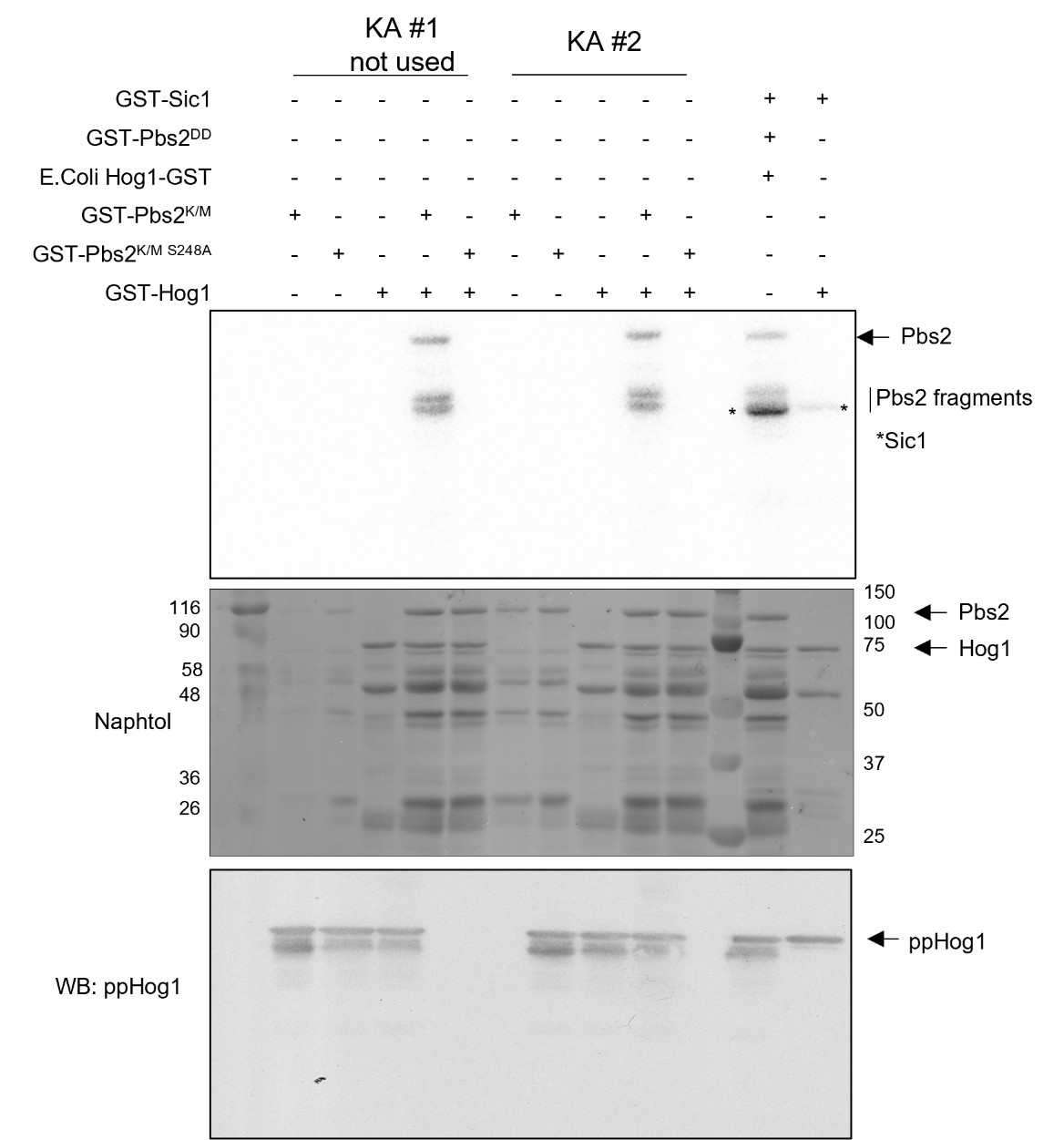
**

A


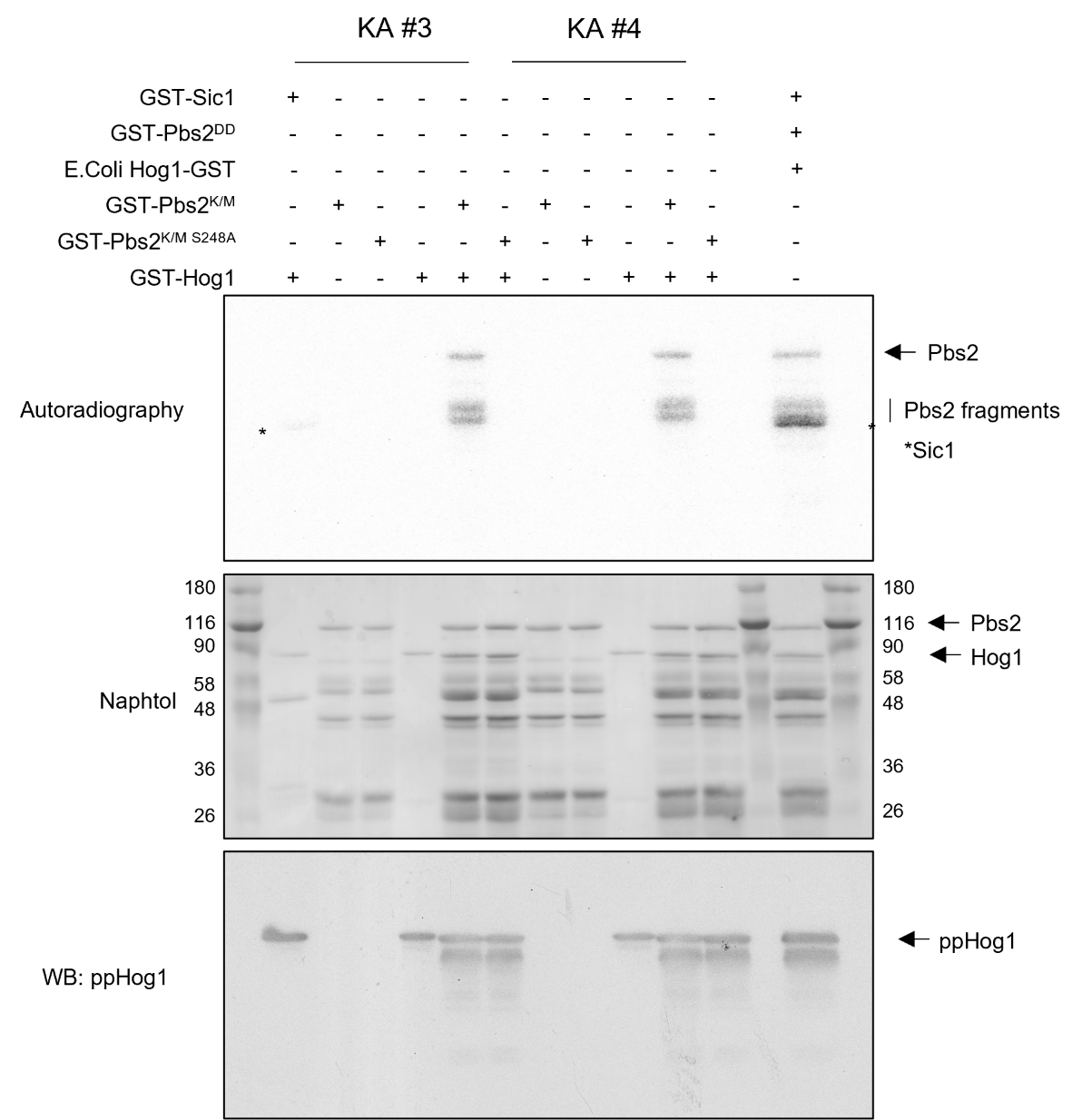


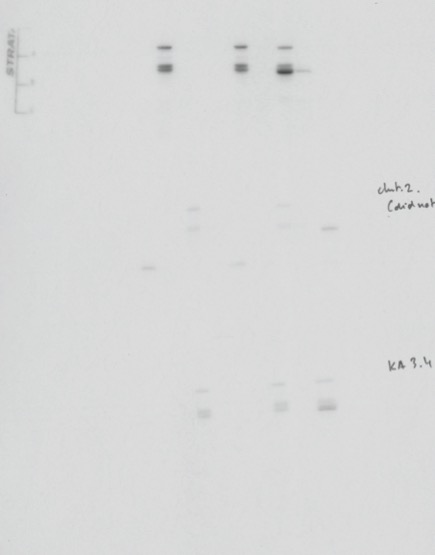


B

**
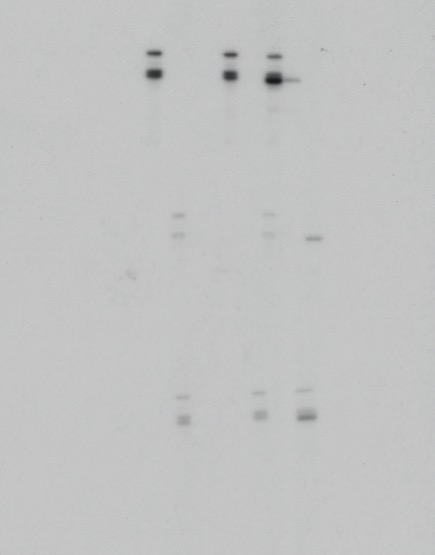
**

**Supp Figure S6: Pbs2 is phosphorylated by Hog1 at Ser248 *in vitro* (full gels of Figure 6A)**

(A) Representative replicate experiments for *in vitro* phosphorylation of Pbs2 by Hog1. Recombinant catalytically inactive GST- Pbs2K/M or Sic1 proteins were purified from E. coli and incubated with active GST-Hog1 purified from NaCl treated wild type yeast (when indicated) in kinase buffer containing ATP. Pbs2K/M, Pbs2K/M S248A was then added in the presence of radioactive ATP. Phosphorylated proteins were resolved by SDS–PAGE, stained (lower panel) and dried before detection by autoradiography (upper panel). As a control GST-Sic1 was incubated with active GST-Hog1 purified from yeast and with active GST-Hog1 activated by a constitutively active Pbs2 (Pbs2DD). (B) Film exposure images of representative replicate experiments with different exposure times.


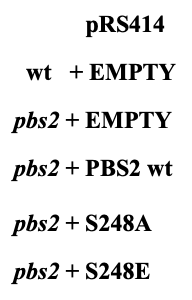

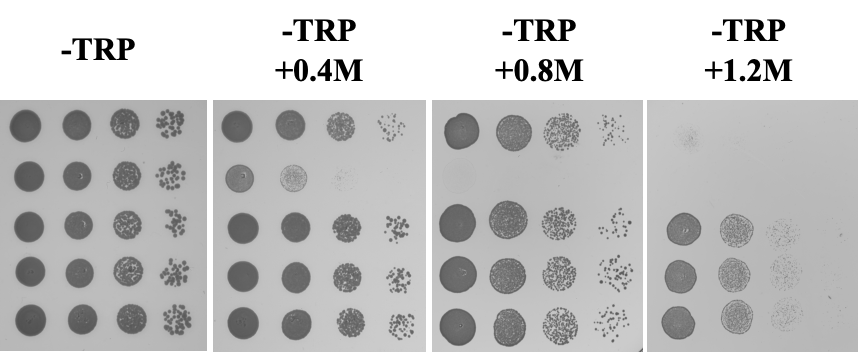


**Supp Figure S7: Hog1-dependent feedback on S248 is not essential for viability**

Growth assay comparing wild-type (WT) and *pbs2Δ* strains harboring an empty pRS414 control plasmid (empty) or pRS414-plasmids expressing wild-type or the Pbs2-S248A mutant. In contrast to *pbs2Δ* controls, growth of the Pbs2-S248A cells was not noticeably affected on plates containing 0.4M or 0.8M NaCl


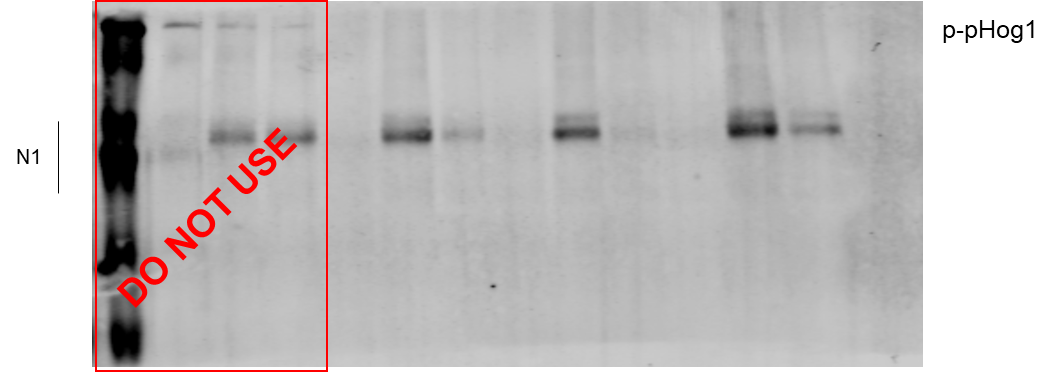


ppHog1


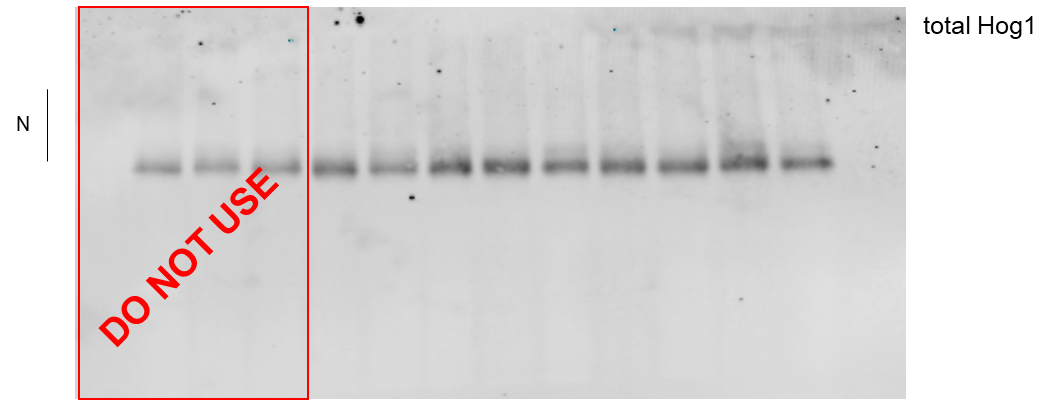


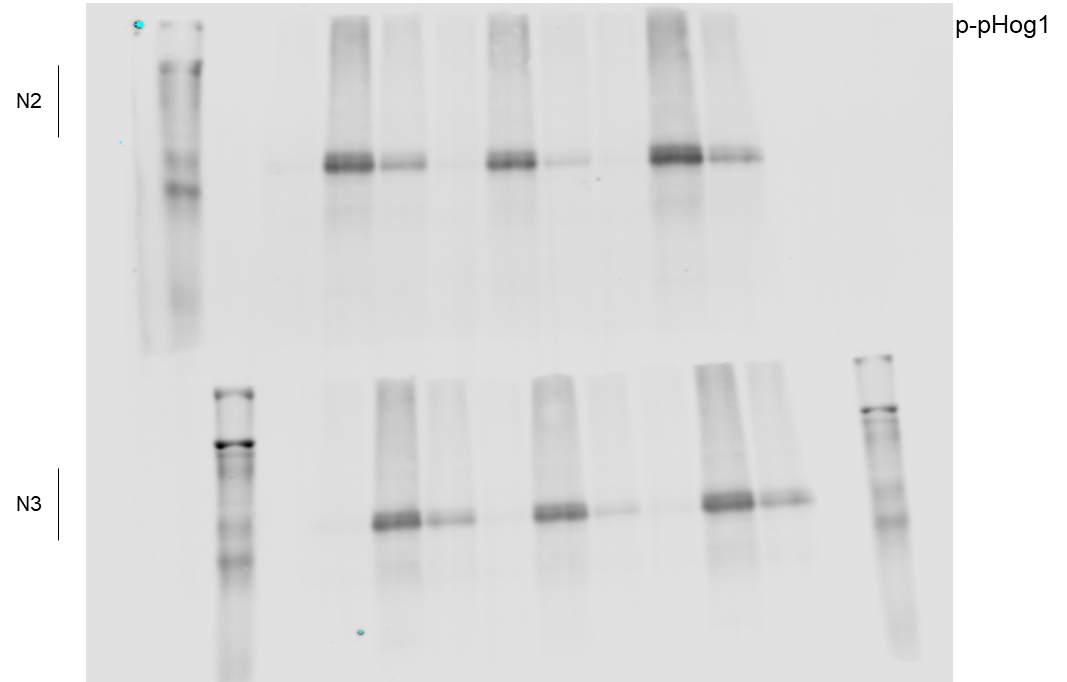

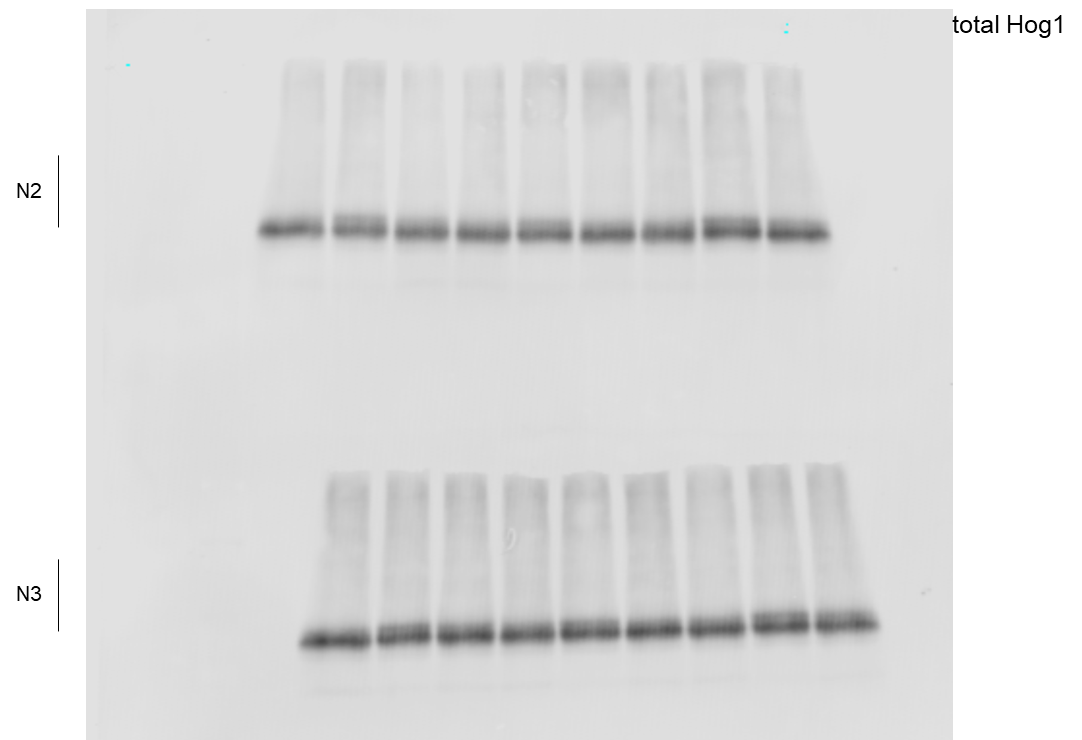


ppHog1


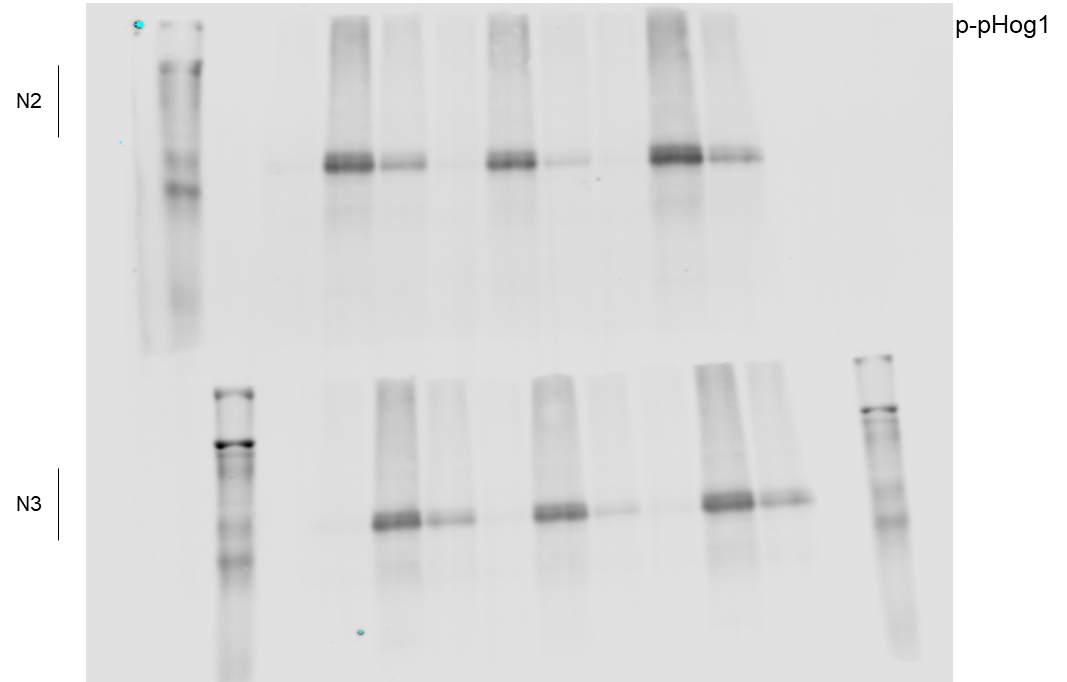


ppHog1

total Hog1


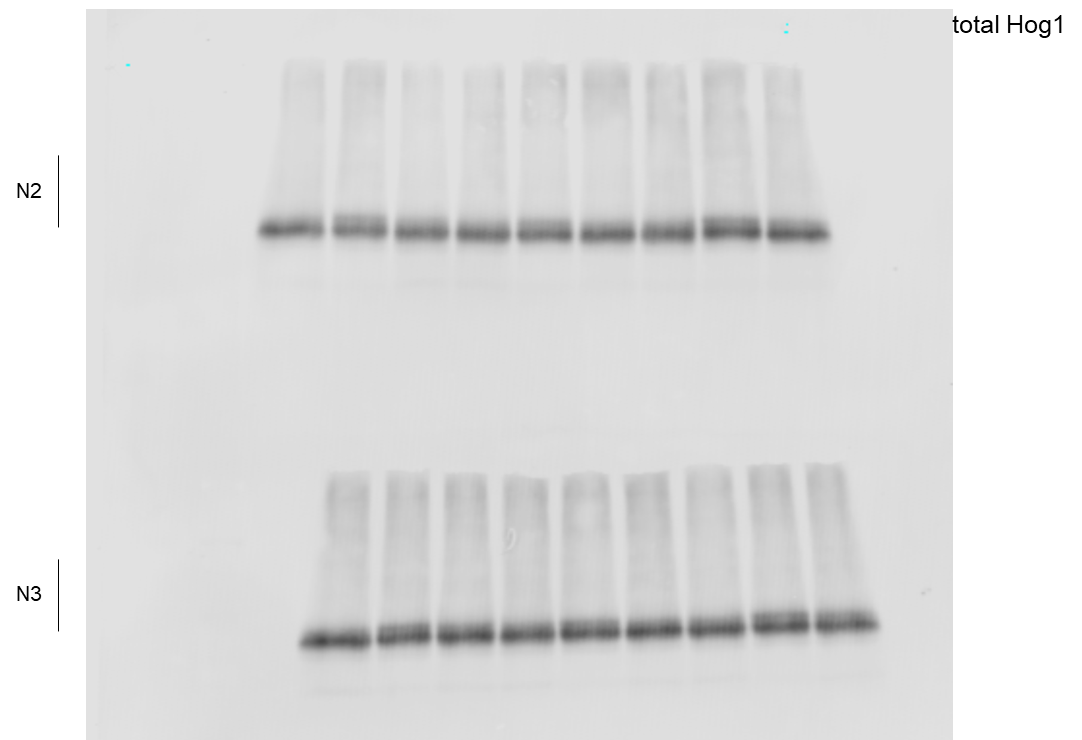


**Supp Figure S8: (full gels of Figure 5F)**

Pbs2-mediated feedback affects Hog1 activation. Hog1 phosphorylation was assessed by western blot using extracts prepared from the indicated strains exposed to 0.4M NaCl after 0, 10 and 15 minutes. Lanes labeled as "do not use" are not related to the experiment and represent internal control for phospo-Hog1 and total Hog1 antibody hybridization for western blots.

| **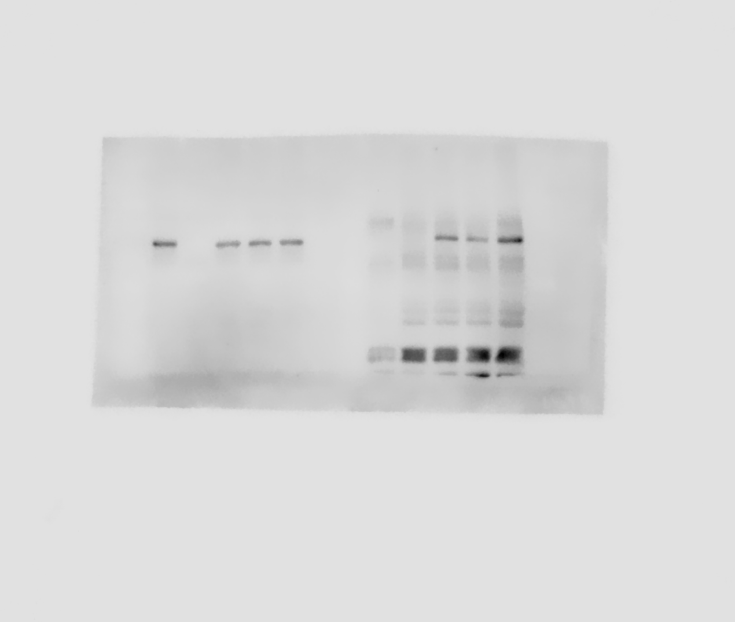**  **Rep1**  Pbs2  ∆  Input Pbs2  E  A  E  WT | **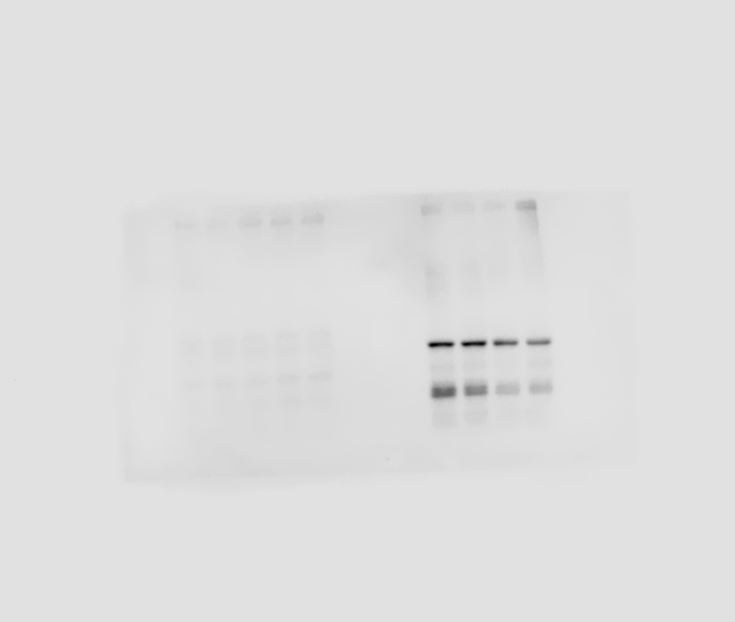**  Hog1-His |
| --- | --- |
| 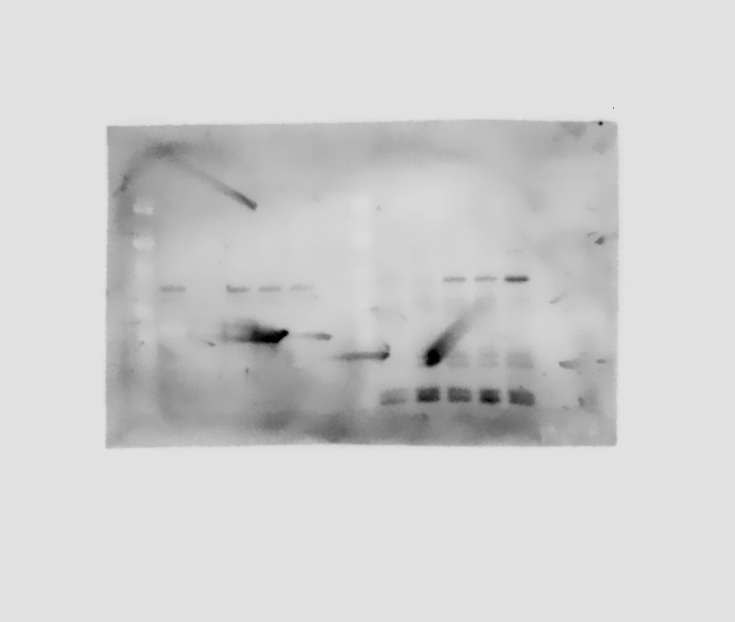  **Rep2**  Pbs2  Input Pbs2 | 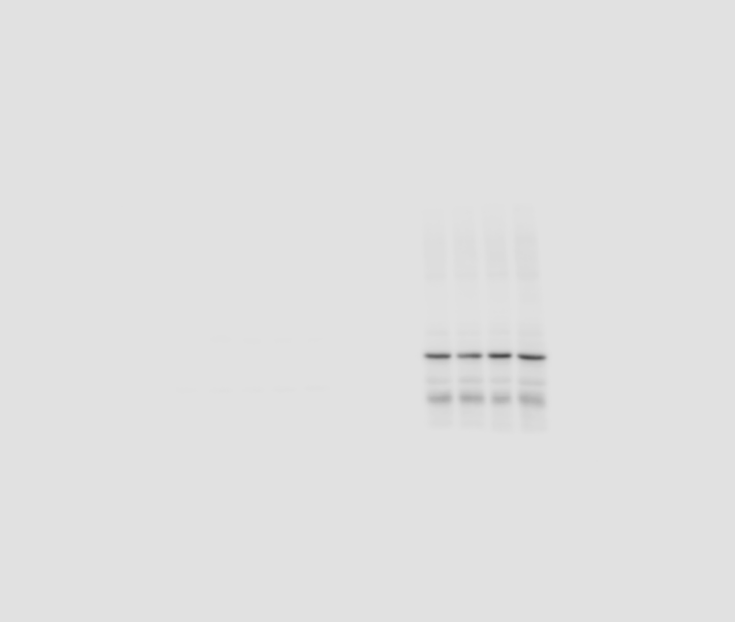  Hog1-His |
| 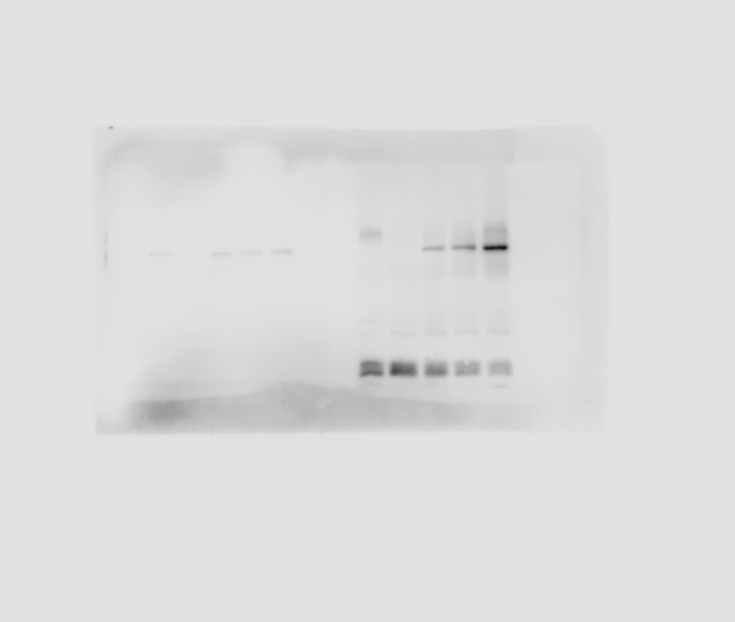  Input Pbs2  Pbs2  Input Pbs2  Pbs2  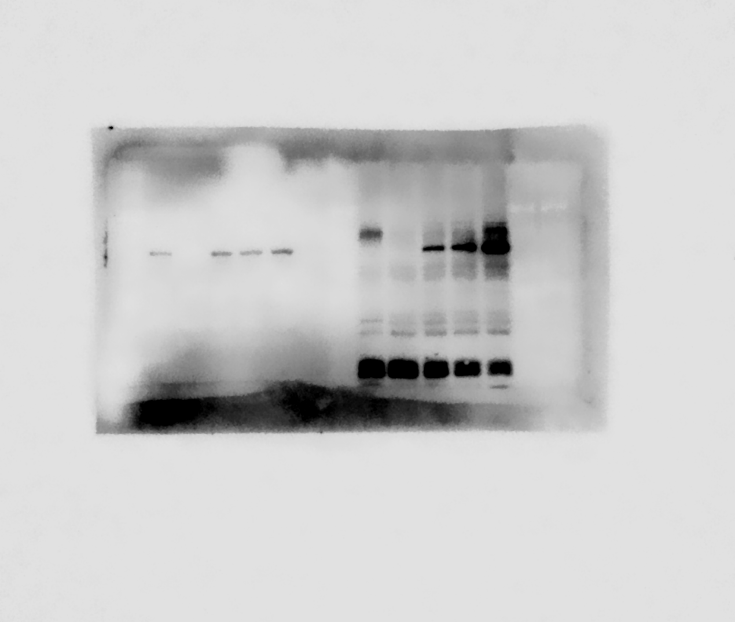  Input Pbs2  Prolonged exposure time  Input Pbs2 | 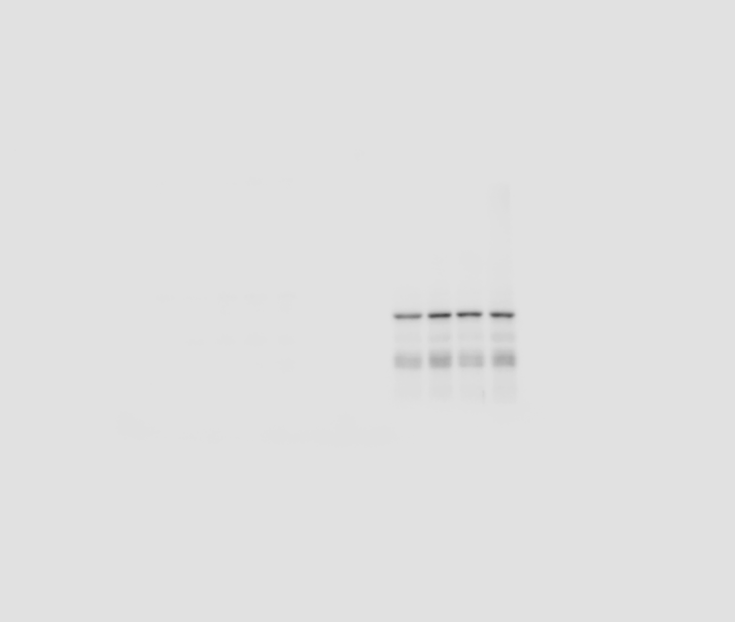  **Rep3**  Hog1-His |
| **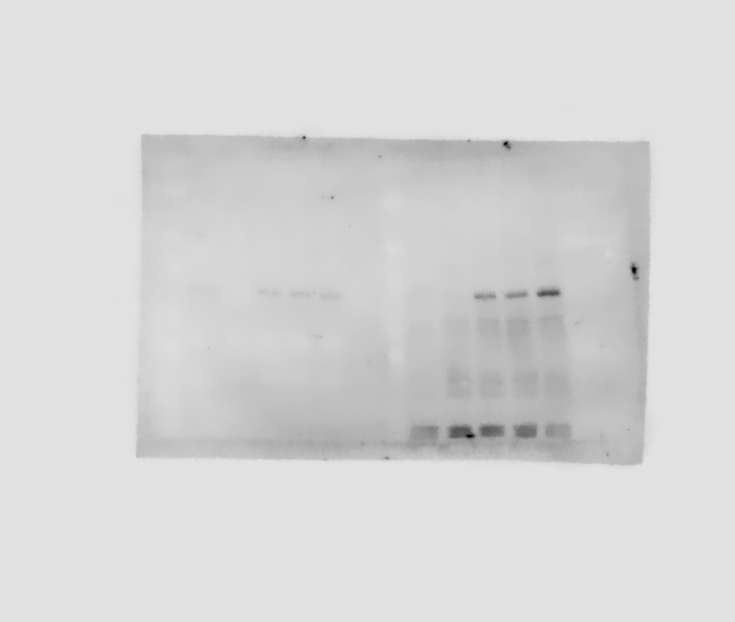**  Input Pbs2  Pbs2  Input Pbs2  **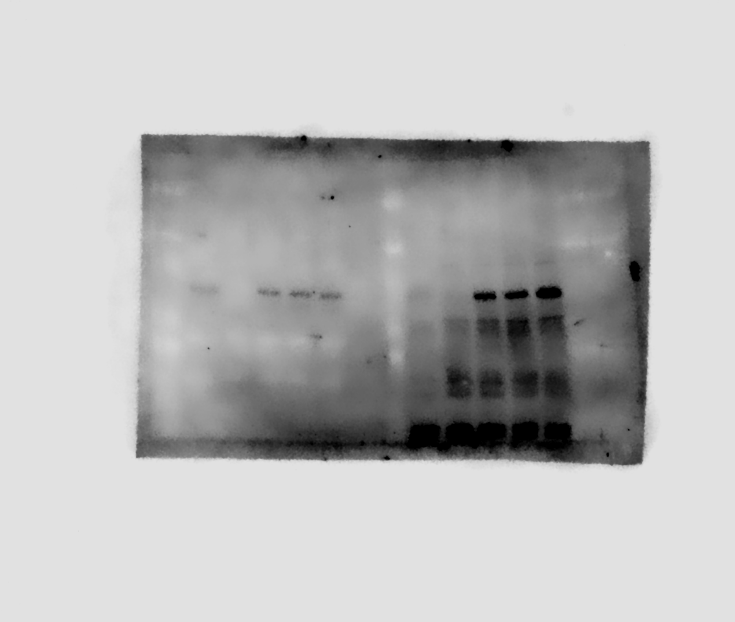**  Input Pbs2  Input Pbs2  Pbs2  Prolonged exposure time | **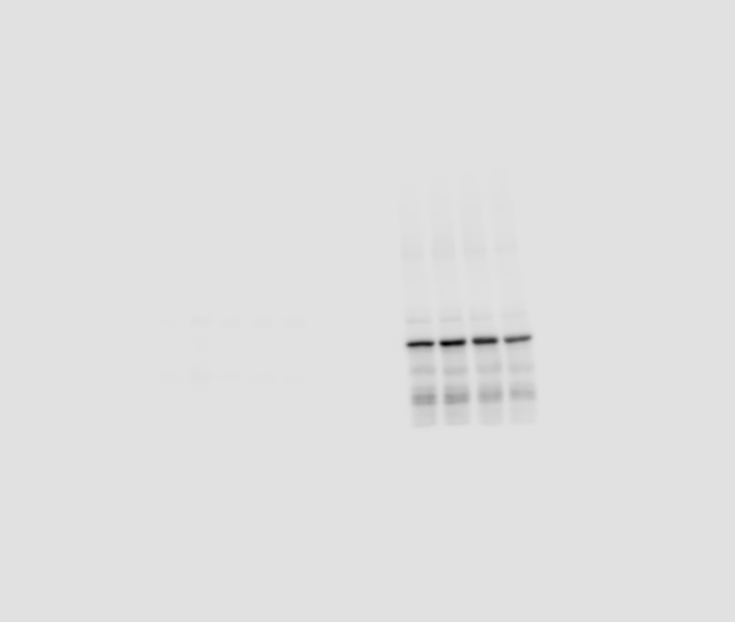**  **Rep4**  Hog1-His |
| 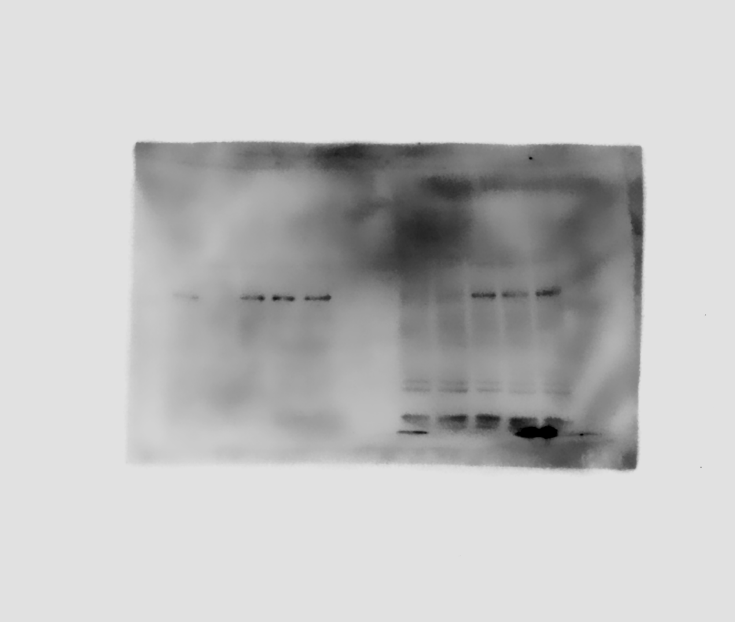  Input Pbs2  Pbs2  Input Pbs2 | 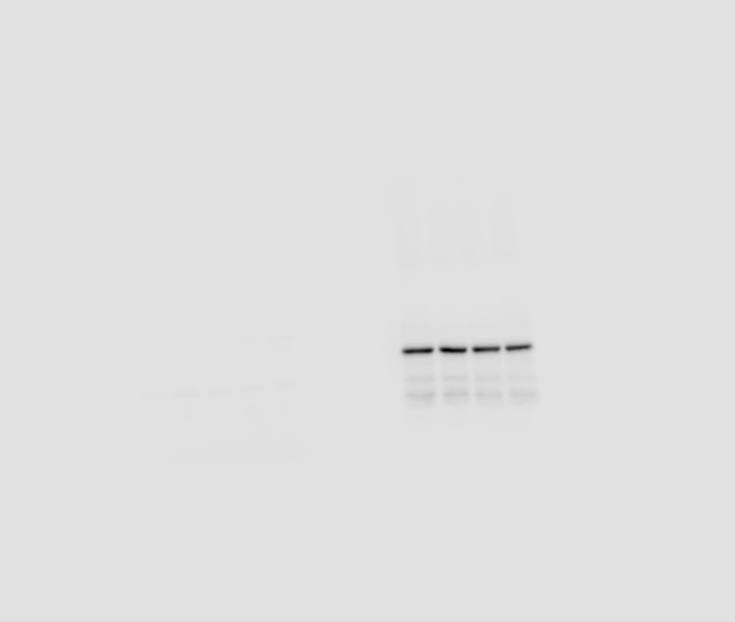  **Rep5**  Hog1-His |
| 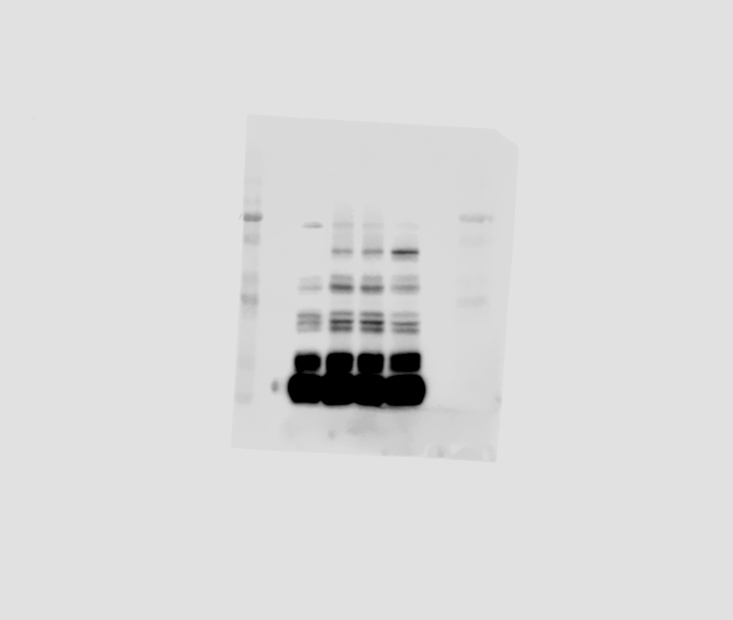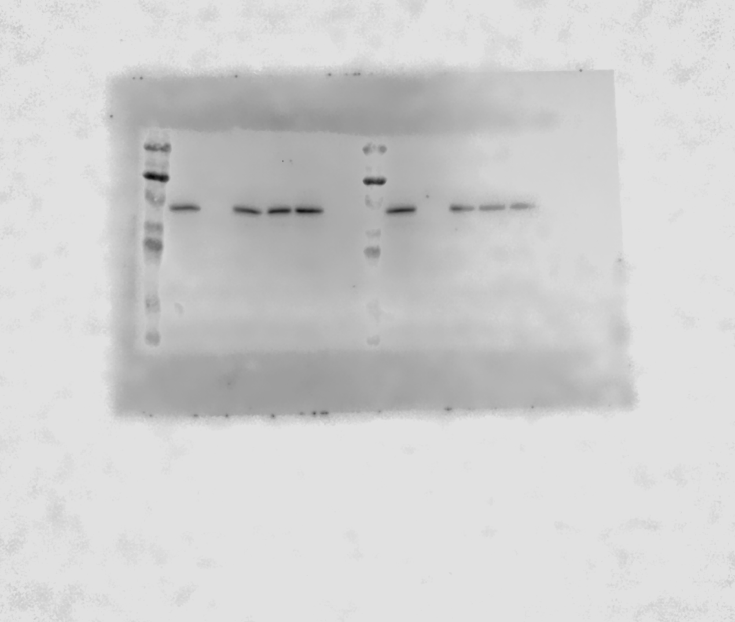  Input Pbs2  Pbs2 | 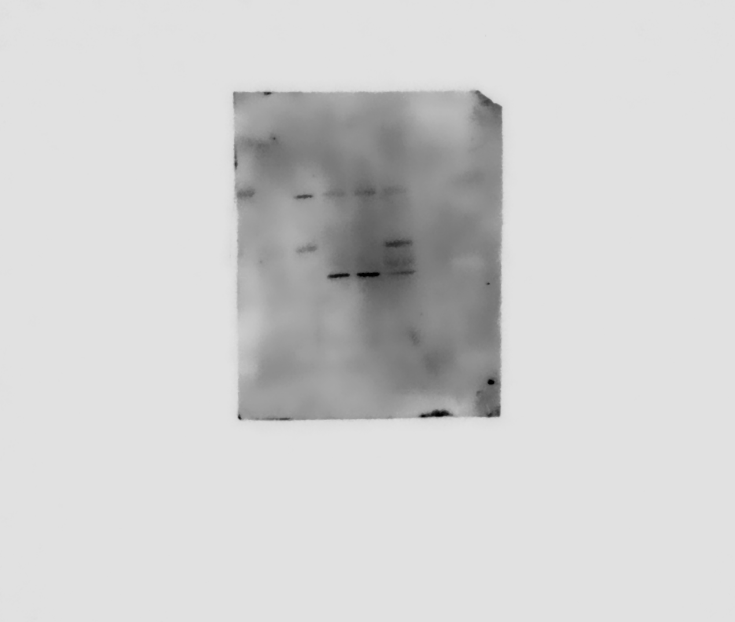  **Rep6**  Hog1-His |
| 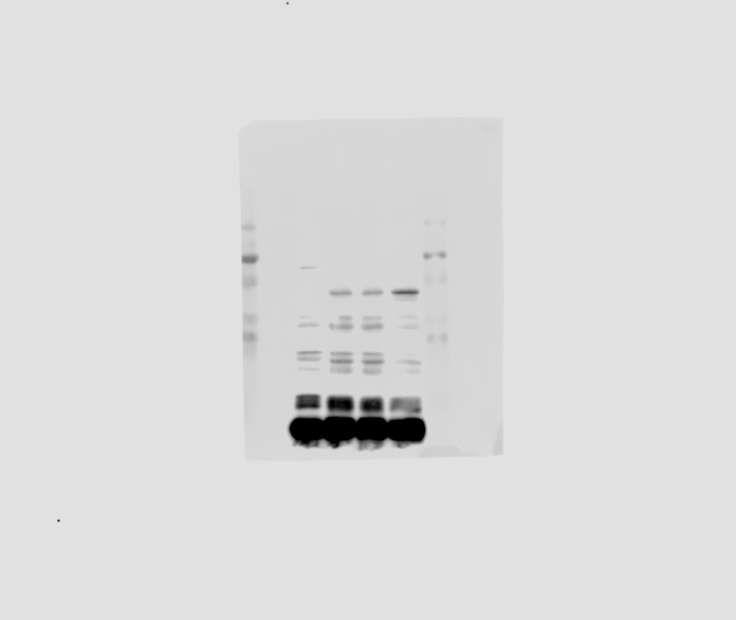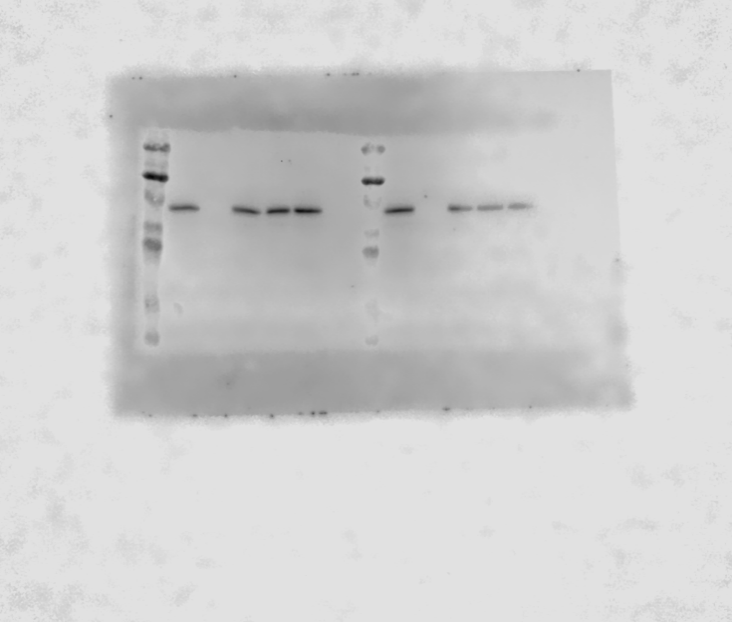  Pbs2  Input Pbs2 | 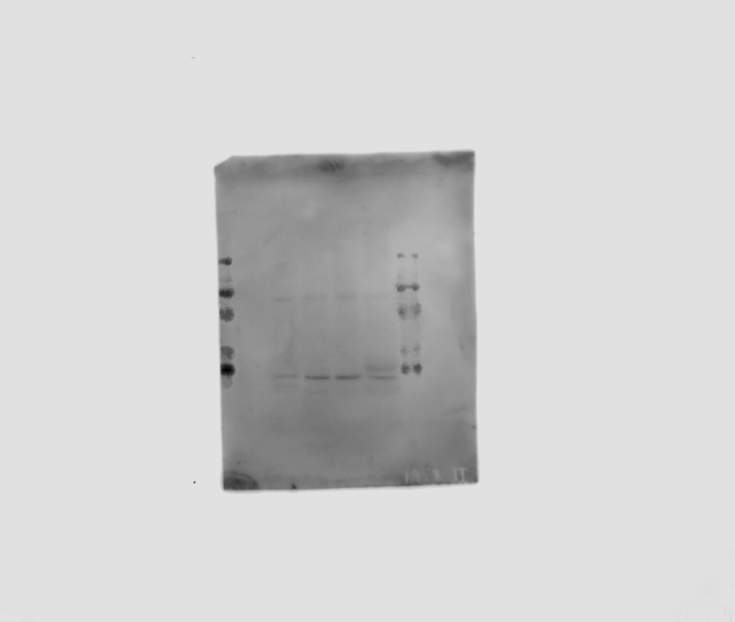  **Rep7**  Hog1-His |

Pbs2

**Supp Figure S9: (full gels of Figure 5 H)**

Full gels of 7 replicates of bead based pull down experiment. Cropped parts indicated in orange were used as representative replicate as shown in Figure 5H.

**
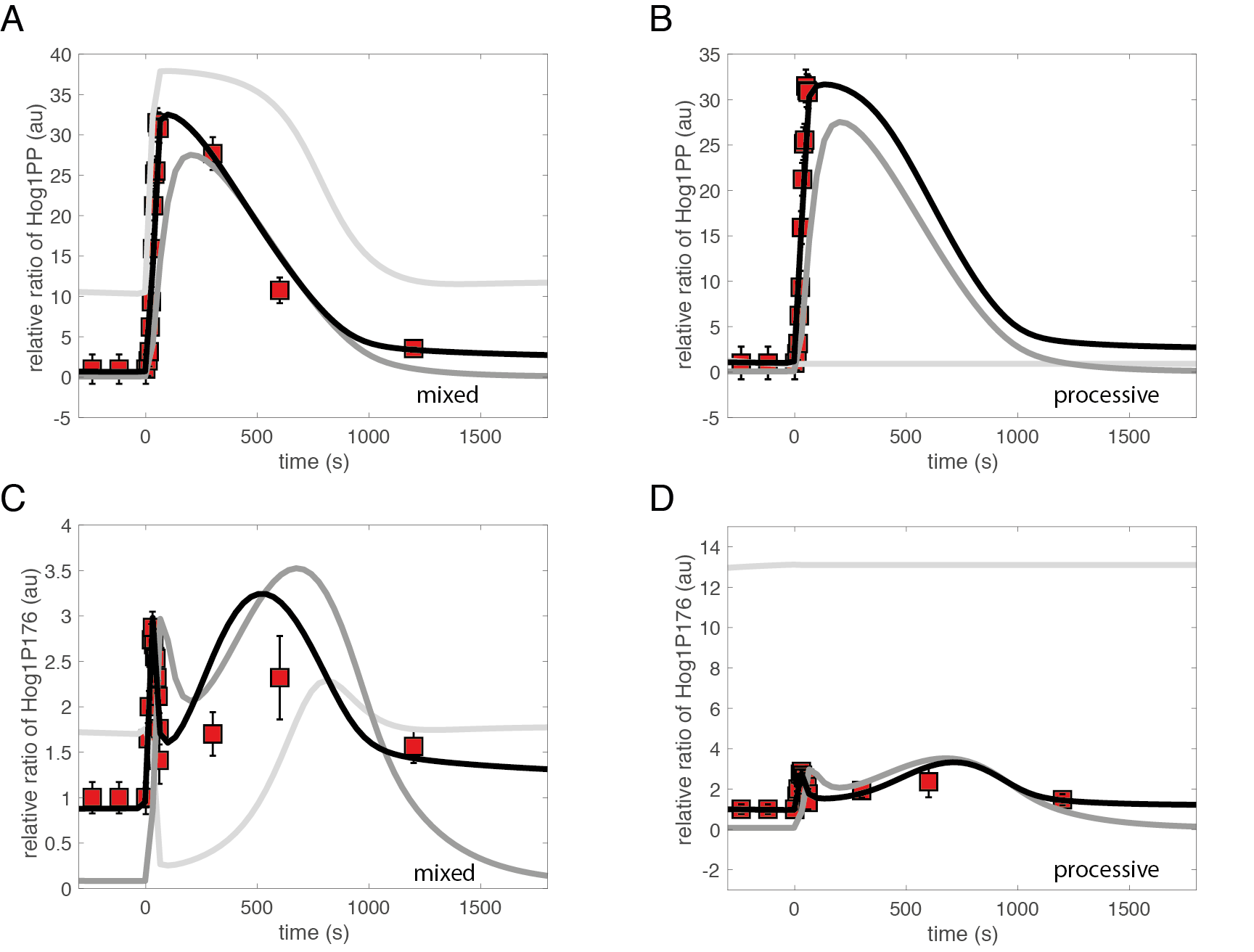
**

**Supp Figure S10: *In silico* over- and under expression of Pbs2 in best fitting mixed or processive model**

(A-D) Simulations are shown of selected Hog1 species from the best fitting model with negative feedback on Ssk2, positive feedback on Pbs2 and either mixed or processive phosphorylation mechanism of Hog1, optimized on data including the monophosphorylated Hog1 species. Graphs show experimental data points used for parameter optimization (red square) and simulations of the model with WT (solid black line), 0.1x (solid dark grey line) and 10x (solid light grey line) levels of Pbs2 concentrations. Time courses of the relative ratio between stimulated and basal levels following salt stimulation (0.4M NaCl) of dual phosphorylated Hog1-PP (A) and mono-phosphorylated Hog1-P176 (C) as the result of a mixed phosphorylation mechanism and dual phosphorylated Hog1-PP (B) and mono-phosphorylated Hog1-P176 (D) as the result of a processive phosphorylation mechanism are shown. (A-D) For the first 60s data by Kanshin et al are presented as values of peptide fold change +/- computational estimate of SEM with n= 1 independent experiment and data by Vaga et al are presented as mean values of peptide fold change +/- standard deviation of n=3 independent experiments.^19,20^


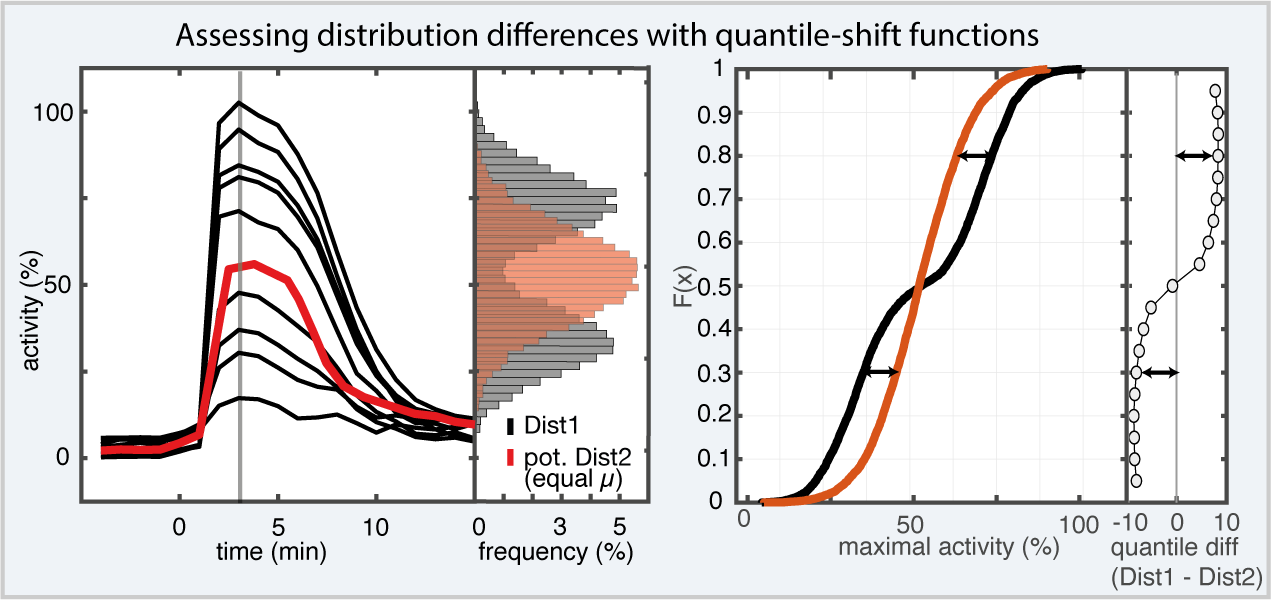


**Supp Figure S11: Hypothetical example of visualization of distribution differences**

Hypothetical example time courses for representative single cells are shown in black with the mean indicated in red. A grey line indicates the timepoint at which activity of each cell is determined. The resulting distribution is displayed in a black histogram and shows bimodal activity distribution. A normal distribution with equal mean serves as null hypothesis distribution and is overlayed in red. Empirical cumulative distribution functions (CDF) of the corresponding distributions are indicated in black or red. Exemplary arrows indicate the difference in the 30% and 80% quantile. Systematic computation of quantile differences allows quantification and assessment of significance of differences in the distributions over their whole range.


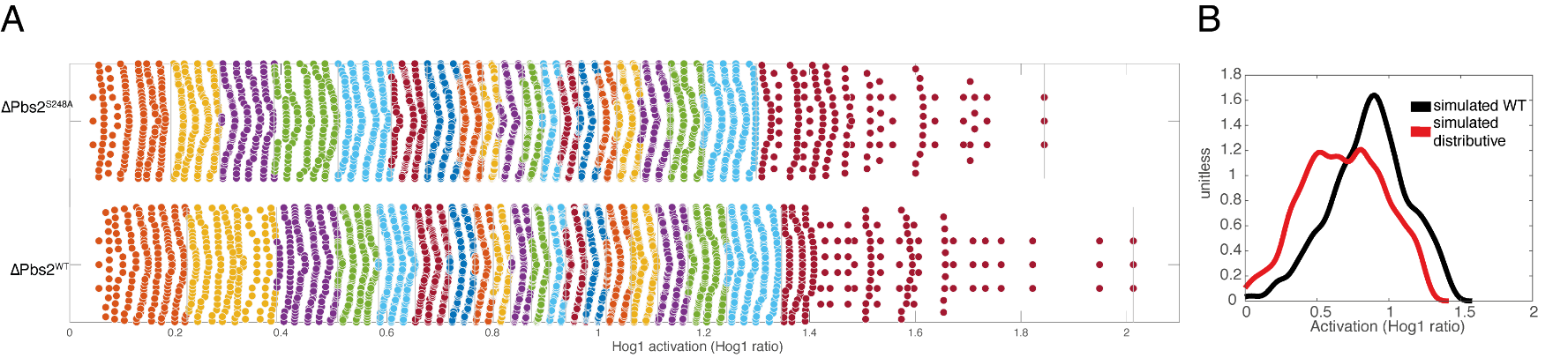


**Supp Figure S12: Changes in Hog1 activity distribution in ΔPbs2^S248A^ are reminiscent of distributive phosphorylation mechanism**

(A) The changes in the distribution of Hog1 activation between ΔPbs2^WT^ and ΔPbs2^S248A^ can be visualized by looking at the differences of the values corresponding to quantiles in a 5% spacing from 5% to 95%. Hog1 ratio values of single cells are depicted and colored according to the quantile they belong to. Proportionally more cells are found at lower Hog1 activation levels (0 to 0.6) for ΔPbs2^S248A^ resulting in lower values for the respective quantiles and thus visually tighter bands of the same color. (B) Kernel density estimation of the distribution of 500 simulations of the best fitting model with either a mixed phosphorylation mechanism (black line) or a distributive phosphorylation mechanism (red line) with starting protein concentrations of Hog1, phosphatases and protein species upstream of Hog1 randomly varied in a range of half to twice their original concentration. The normalized maximal Hog1 activation given by the Hog1 nuclear to cytosolic ratio after addition of 0.4M NaCl is depicted.

| **Condition** | **Description** |  |  | **Source** |
| --- | --- | --- | --- | --- |
| Condition1 | 0.4M NaCl, *pbs2Δ* | a | Data are presented as mean values +/-Computational estimate of SEM, n=785 cells examined over 3 independent experiments | this study |
|  |  | b | Data are presented as mean values +/-Computational estimate of SEM, n=785 cells examined over 3 independent experiments | this study |
|  |  | c | Data are presented as mean values +/-Computational estimate of SEM, n=785 cells examined over 3 independent experiments | this study |
|  |  | d | Data are presented as mean values +/-Computational estimate of SEM, n=785 cells examined over 3 independent experiments | this study |
| Condition2 | 0.2M NaCl, *pbs2Δ* | a | Data are presented as mean values +/-Computational estimate of SEM, n=137 cells examined over 1 independent experiments | this study |
| Condition3 | 0.4M NaCl, *ssk1Δ* | a |  |  |
|  |  | b | Data are presented as mean values +/-Computational estimate of SEM, n=140 cells examined over 1 independent experiments | ^13^ |
| Condition4 | 0.4M NaCl, pulse | a | Data are presented as mean values +/-Computational estimate of SEM, n=3 independent experiments | ^4^ |
|  |  | b | Data are presented as mean values +/-Computational estimate of SEM, n=777 cells examined over 2 independent experiments | this study |
|  |  | c | Data are presented as mean values +/-Computational estimate of SEM, n=777 cells examined over 2 independent experiments | this study |
| Condition5 | 0.4M NaCl, WT | a | Data are presented as values +/- Computational estimate of SEM, n=1 independent experiments | ^4^ |
|  |  | b | Data are presented as mean values +/-Computational estimate of SEM, n=3 independent experiments | ^4^ |
|  |  | c | Data are presented as mean values +/-Computational estimate of SEM, n=1156 cells examined over 3 independent experiments | this study |
|  |  | d | Data are presented as quantified values +/- Computational estimate of SEM, n=1 independent experiments | ^14^ |
|  |  | e | Data are presented as quantified values +/- Computational estimate of SEM, n=1 independent experiments | ^14^ |
|  |  | f | Data are presented as mean values +/-Computational estimate of SEM, n=1156 cells examined over 3 independent experiments | this study |
| Condition6 | 0.4M NaCl, Cycloheximide | a |  |  |
|  |  | b | Data are presented as mean values +/-Computational estimate of SEM, n=50-300 cells examined over 2 independent experiments | ^15^ |

**Supp Table S1:**

Description of each condition and the data used for the parameter optimization of the volume sub-model. The best fit for each condition is described in Supp Figure S2.

| **Condition** | **Description** |  |  | **Source** |
| --- | --- | --- | --- | --- |
| Condition1 | 0.4M NaCl, WT | a | Data are presented as mean values +/-Computational estimate of SEM, n=1156 cells examined over 3 independent experiments | this study |
|  |  | b | Data are presented as mean values +/- standard deviation, n=1156 cells examined over 3 independent experiments | this study |
|  |  | c | Data are presented as values +/-Computational estimate of SEM, n= 1 independent experiment | ^16^ |
|  |  | d | Data are presented as mean values +/- standard deviation, n=3 independent experiments | ^18^ |
|  |  | e | Data are presented as values +/-Computational estimate of SEM, n= 1 independent experiment | ^16^ |
|  |  | f | Data are presented as mean values +/- standard deviation, n=3 independent experiments | ^18^ |
|  |  | g | Data are presented as values +/-Computational estimate of SEM, n= 1 independent experiment | ^16^ |
|  |  | h | Data are presented as mean values +/- standard deviation, n=3 independent experiments | ^18^ |
|  |  | i | Data are presented as mean values +/- Computational estimate of SEM, n= 3 independent experiment | ^11^ |
|  |  | j | Data are presented as values +/-Computational estimate of SEM, n= 1 independent experiment | ^16^ |
|  |  | k | Data are presented as values +/-Computational estimate of SEM, n= 1 independent experiment | ^16^ |
|  |  | l | Data are presented as mean values +/- standard deviation, n=3 independent experiments | ^18^ |
|  |  | m | Data are presented as values +/-Computational estimate of SEM, n= 1 independent experiment | ^16^ |
|  |  | n | Data are presented as mean values +/- standard deviation, n=3 independent experiments | ^4^ |
|  |  | o | Data are presented as values +/-Computational estimate of SEM, n= 1 independent experiment | ^16^ |
| Condition2 | 0.4M NaCl, *ptp2Δ* | a | Data are presented as mean values +/-Computational estimate of SEM, n=1156 cells examined over 3 independent experiments | this study on basis of ^20^ |
|  |  | b | Data are presented as quantified values +/- Computational estimate of SEM, n=1 independent experiments | ^19^ |
| Condition3 | 0.4M NaCl, *ptp3Δ* | a | Data are presented as mean values +/-Computational estimate of SEM, n=1156 cells examined over 3 independent experiments | this study on basis of ^20^ |
|  |  | b | Data are presented as quantified values +/- Computational estimate of SEM, n=1 independent experiments | ^19^ |
| Condition4 | 0.4M NaCl, *ptp2/3Δ* | a | Data are presented as mean values +/-Computational estimate of SEM, n=1156 cells examined over 3 independent experiments | this study on basis of ^20^ |
|  |  | b | Data are presented as quantified values +/- Computational estimate of SEM, n=1 independent experiments | ^19^ |
| Condition5 | 0.4M NaCl, *ste11Δ* | a | Data are presented as mean values +/-Computational estimate of SEM, n=105 cells examined over 1 independent experiments | ^13^ |
|  |  | b | Data are presented as mean values +/-Computational estimate of SEM, n=105 cells examined over 1 independent experiments | ^13^ |
|  |  | c | Data are presented as mean values +/- Computational estimate of SEM, n= 3 independent experiment | ^11^ |
| Condition6 | 0.4M NaCl, *ssk1Δ* | a | Data are presented as mean values +/-Computational estimate of SEM, n=123 cells examined over 1 independent experiments | ^13^ |
|  |  | b | Data are presented as mean values +/-Computational estimate of SEM, n=123 cells examined over 1 independent experiments | ^13^ |
|  |  | c | Data are presented as mean values +/- Computational estimate of SEM, n= 3 independent experiment | ^11^ |
| Condition7 | 0.2M NaCl, WT | a | Data are presented as mean values +/-Computational estimate of SEM, n=691 cells examined over 2 independent experiments | this study |
|  |  | b | Data are presented as mean values +/-Computational estimate of SEM, n=691 cells examined over 2 independent experiments | this study |
| Condition8 | 0.1M NaCl, WT | a | Data are presented as mean values +/-Computational estimate of SEM, n=641 cells examined over 2 independent experiments | this study |
|  |  | b | Data are presented as mean values +/-Computational estimate of SEM, n=641 cells examined over 2 independent experiments | this study |
| Condition9 | 0.2M NaCl, pulse, t0=2min | a | Data are presented as mean values +/-Computational estimate of SEM, n=50-300 cells examined over 2 independent experiments | ^15^ |
| Condition10 | 0.2M NaCl, pulse, t0=4min | a | Data are presented as mean values +/-Computational estimate of SEM, n=50-300 cells examined over 2 independent experiments | ^15^ |
| Condition11 | 0.2M NaCl, pulse, t0=8min | a | Data are presented as mean values +/-Computational estimate of SEM, n=50-300 cells examined over 2 independent experiments | ^15^ |
| Condition12 | 0.2M NaCl, pulse, t0=16min | a | Data are presented as mean values +/-Computational estimate of SEM, n=50-300 cells examined over 2 independent experiments | ^15^ |
| Condition13 | 0.4M NaCl, *ssk2-8A* | a | Data are presented as mean values +/-Computational estimate of SEM, n=at least 600 cells examined over 3 independent experiments | ^17^ |
| Condition14 | *hog1as* | a | Data are presented as mean values +/- standard deviation, n=3 independent experiment | ^11^ |
| Condition15 | *hog1as, ssk2Δ* | a | Data are presented as mean values +/- standard deviation, n=3 independent experiment | ^11^ |
| Condition16 | 0.4M NaCl, *hog1as* | a | Data are presented as mean values +/-SEM, n=2 independent experiment | ^12^ |

**Supp Table S2:**

Description of each condition and the data used for the parameter optimization of the overcomplete model. The best fit for each condition is described in Supp Figure S3.

| **Strain ID** | **Description** | **Background** | **Source** | **Used in figure** |
| --- | --- | --- | --- | --- |
| BY4741 | MATa hisΔ1; leuΔ0; met15Δ0; ura3Δ0 | BY4741 | OpenBiosystems | Parental |
| W303 | leu2-3,112 trp1-1 can1-100 ura3-1 ade2-1 his3-11,15 | W303 | Lab collection | Parental |
| Pbs2Δ | pbs2Δ | W303 | Lab collection | Parental |
| yMU49 | HTA2-CFP | BY4741 | Lab collection | Parental |
| yMM001 | HOG1-YFP HTA2-CFP | BY4741 | This study | Parental |
| yMU19 | HOG1-YFP | W303 | Lab collection | Parental |
| yHS206 | Hta2-CFP Hog1-mCherry pbs2Δ | W303 | Lab collection | Parental |
| yMM003 | HOG1-YFP HTA2-CFP Fus3_SKARS_reporter_mCherry_URA3 | BY4741 | This study | Fig.2 Supp Fig.S3 |
| yMM008 | Hog1-YFP HTA2-CFP Fus3_SKARS_reporter_mCherry_URA3 pbs2Δ | BY4741 | This study | Supp Fig.S2 |
| yMM004 | HOG1-YFP HTA2-CFP pSTL1-dPSTRr | BY4741 | This study | Fig.2 Supp Fig.S3 |
| yHS29 | HOG1-YFP HTA2-CFP pRPS2-Cherry-TMD | W303 | Lab collection | Fig.3C |
| yMM009 | HTA2-CFP HOG1-mCherry pbs2Δ::PBS2-WT | W303 | This study | Fig. 5C, 6B |
| yMM010 | HTA2-CFP HOG1-mCherry pbs2Δ::PBS2-S248A | W303 | This study | Fig. 5C, 6A |
| yJT367 | HTA2-CFP-KAN, HOG1-6HA::HIS, pbs2Δ::PBS2-wt | BY4741 | This study | Fig 5E |
| yJT369 | HTA2-CFP-KAN, HOG1-6HA::HIS, pbs2Δ::PBS2-S248E | BY4741 | This study | Fig 5E |
| YMN455 | HOG1-6HA::HIS PBS2::URA |  | Lab collection | Parental |
| YMN457 | PBS2-S248E |  | This study | Fig 5F, Supp Fig S8 |
| YMN459 | PBS2-S248A |  | This study | Fig 5F, Supp Fig S8 |
| YMN460 | PBS2-WT |  | This study | Fig 5F, Supp Fig S8 |
| YMN551 | *PBS2::URA HOG1::KAN* | BY4741 | This study | Fig 5G/H,  Supp Fig S9 |
| YMN552 | *HOG1::KAN* Pbs2^S248E^ | BY4741 | This study | Fig 5G/H, Supp Fig S9 |
| YMN553 | *HOG1::KAN* Pbs2^S248A^ | BY4741 | This study | Fig 5G/H,  Supp Fig S9 |
| YMN554 | HOG1::KAN Pbs2^WT^ | BY4741 | This study | Fig 5G/H,  Supp Fig S9 |

**Supp Table S3: Strain list**

| **Plasmid ID** | **Description** | **Source** |
| --- | --- | --- |
| pED45 | Fus3_SKARS_reporter_mCherry_URA3 | ^21^ |
| pSP135 | pAgTEF1-natMX-tAgTEF1 | Lab collection |
| pDA183 | pSIVU pRPL24A mCherry SZ2 tSIF2 -- pSTL1 UbiY 2xSv40NLS NewLinkerSZ1 | ^22^ |
| pGY75 | GST-PBS2 K/M (pGEX6P1mut) | This study |
| pGY77 | GST-PBS2 K/M S248A (pGEX6P1mut) | This study |
| pGY86 | PBS2 WT pRS414 | This study |
| pGY87 | PBS2 S248A pRS414 | This study |
| pGY88 | PBS2 S248E pRS414 | This study |
| pEN133 | TEG1-Hog1 pRS426 | This study |
| pFD236 | Hog1-GFP_URA3 | Lab collection |
| pEN111 | Hog1-HIS | This study |

**Supp Table S4: Plasmid list**

| **Primer sequence (5’ to 3’)** | **Name** | **Resulting strain** |
| --- | --- | --- |
| AGGGTAGCTGCTATTGTGGG | Pbs2_prom forward | YMN457, YMN459, YMN460 |
| TGCTTTTTTTTTGTTGTTATATTCACGTGCCTGTTTGCTTTTATTTGGATATTAACGCTATAAACCACCCATATGTAATG | Pbs2_ORF reverse |  |
|  |  |  |
| CAAAGGGAAAACAGGGAAAACTACAACTATCGTATATAATACAGCTGAAGCTTCGTACGC | hog1::KAN forward | YMN551, YMN552, YMN553, YMN554 |
| GAAGTAAGAATGAGTGGTTAGGGACATTAAAAAAACACGTATAGGCCACTAGTGGATCTG | hog1::KAN reverse |  |
|  |  |  |
| GTACCTGTTTACAGTTGAG | Hog1 forward | yMM001 |
| GTTAGAATTAATGAATGAGAGC | Hog1 reverse |  |

**Supp Table S5: Primer list**

| Observables | Definition | Additional comment (respective position in Supp Figure 3) |
| --- | --- | --- |
| volume | (V_os + 1874)/(init_V_os + 1874) | Normalized change in volume (Cond1: volume; Cond5: volume; Cond6: volume; Cond7: volume; Cond8: volume) |
| hog1_ratio | (Hog1PPn + Hog1n + Hog1P174n + Hog1P176n)/(Hog1PPc + Hog1c + Hog1P174c + Hog1P176c + Hog1PPc_Pbs2PP + Hog1P174c_Pbs2PP + Hog1P176c_Pbs2PP + Hog1PPc_Pbs2PP_phosphorylated + Hog1P176c_Pbs2PP_phosphorylated + Hog1P174c_Pbs2PP_phosphorylated + Hog1c_Pbs2PP_phosphorylated + Hog1c_Pbs2 + Hog1P176c_Pbs2 + Hog1P174c_Pbs2 + Hog1PPc_Pbs2 + Hog1c_Pbs2_phosphorylated + Hog1P176c_Pbs2_phosphorylated + Hog1P174c_Pbs2_phosphorylated + Hog1PPc_Pbs2_phosphorylated) | Ratio between nuclear and cytosolic Hog1 (Cond1: Hog1 ratio; Cond5: Hog1 ratio; Cond6: Hog1 ratio; Cond7: Hog1 ratio; Cond8: Hog1 ratio; Cond13: Hog1 ratio) |
| output_hog1_ratio_mutant | See hog1_ratio | Ratio between nuclear and cytosolic Hog1 for data set with different error parameter (Cond2: Hog1 ratio mutant; Cond3: Hog1 ratio mutant; Cond4: Hog1 ratio mutant; Cond9: Hog1 ratio mutant; Cond10: Hog1 ratio mutant; Cond11: Hog1 ratio mutant; Cond12: Hog1 ratio mutant |
| output_Hog1PP | (molecules_Hog1PPc + molecules_Hog1PPn) /(starting_molecules_Hog1PPc + starting_molecules_Hog1PPn) | Relative ratio of double phosphorylated Hog1 (Cond1: Hog1PP 60s) |
| output_Hog1PP_vaga | See output_Hog1PP | Relative ratio of double phosphorylated Hog1 for data set with different error parameter (Cond1: Hog1PP) |
| Hog1_total_phosphorylation | (molecules_Hog1P174 + molecules_Hog1P176 + molecules_Hog1PPc + molecules_Hog1PPn)/(molecules_Hog1P174 + molecules_Hog1P176 + molecules_Hog1PPc + molecules_Hog1PPn +molecules_Hog1c + molecules_Hog1n)*100 | Percentage of mono or double phosphorylated Hog1 (Cond1: Hog1 total phosphorylation; Cond5: Hog1 total phosphorylation; Cond6: Hog1 total phosphorylation |
| Hog1_total_phosphorylation_inhibition | See Hog1_total_phosphorylation | Percentage of mono or double phosphorylated Hog1 for data sets with different error parameter (Cond14: Hog1 total phosphorylation; Cond15: Hog1 total parameter) |
| output_tyrosine_phosphorylation | ( molecules_Hog1P176 + molecules_Hog1PPc + molecules_Hog1PPn)/(molecules_Hog1P174 + molecules_Hog1P176 + molecules_Hog1PPc + molecules_Hog1PPn +molecules_Hog1c + molecules_Hog1n)*100 | Percentage with double phosphorylated or mono phosphorylated at tyrosine 176 Hog1 (Cond2: Hog1 tyrosine phosphorylation; Cond3: Hog1 tyrosine phosphorylation; Cond4: Hog1 tyrosine phosphorylation) |
| output_Pbs2P | (molecules_Pbs2P) / starting_molecules_Pbs2P | Relative rate of monophosphorylated Pbs2 (Cond1: Pbs2P 60s) |
| output_Hog1P174 | (molecules_Hog1P174) / starting_molecules_Hog1P174 | Relative ratio of Hog1 monophosphorylated at Threonine 174 (Cond1: Hog1P174 60s) |
| output_Hog1P174_vaga | See output_Hog1P174 | Relative ratio of Hog1 monophosphorylated at Threonine 174 for data set with different error parameter (Cond1: Hog1P174) |
| output_Hog1P176 | (molecules_Hog1P176) / starting_molecules_Hog1P176 | Relative ratio of Hog1 monophosphorylated at Tyrosine174 (Cond1: Hog1P176 60s) |
| output_Hog1P176_vaga | (molecules_Hog1P176) / starting_molecules_Hog1P176 | Relative ratio of Hog1 monophosphorylated at Tyrosine 174 for data set with different error parameter (Cond1: Hog1P176) |
| output_Ssk2_inactivation | (molecules_Ssk2_second_inactive )/(starting_molecules_Ssk2_second_inactive) | Relative ratio of double phosphorylated Ssk2 (Cond1: Ssk2PP feedback 60s) |
| output_Ssk2_inactivation_vaga | See output_Ssk2_inactivation | Relative ratio of double phosphorylated Ssk2 for data set with different error parameter (Cond1: Ssk2PP feedback) |
| output_Ssk2_inactivation_mono | (molecules_Ssk2_inactive )/(starting_molecules_Ssk2_inactive) | Relative ratio of mono phosphorylated Ssk2 (Cond1: Ssk2P feedback) |
| output_double_phosphorylation_ratio | (molecules_Hog1PPc + molecules_Hog1PPn)/(total_hog1) | Percentage of double phosphorylated Hog1 (Cond16: Hog1PP percentage) |
| output_Gpd1_ratio | (molecules_Gpd1 )/(starting_molecules_Gpd1) | Relative ratio of Gpd1 expression (Cond1: Gpd1) |
| output_Pbs2_phosphorylation_ratio | (molecules_Pbs2_phosphorylated)/ (starting_molecules_Pbs2_phosphorylated) | Relative ratio of Pbs2 phosphorylated by feedback (Cond1: Pbs2P feedback) |

| Species/Parameter | Definition | Additional comments |
| --- | --- | --- |
| to_molecule_number_cytosol | V_os_cytosol * 6.02214 | Conversion factor for concentration to absolute number of molecules in the cytosol |
| to_molecule_number_nucleus | V_os_nucleus * 6.02214 | Conversion factor for concentration to absolute number of molecules in the nucleus |
| to_molecule_number_cytosol_start | Init_V_os_cytosol * 6.02214 | Conversion factor for starting concentration to absolute number of molecules in the cytosol |
| to_molecule_number_nucleus_start | Init_V_os_nucleus * 6.02214 | Conversion factor for starting concentration to absolute number of molecules in the nucleus |
| molecules_Hog1PPc | (Hog1PPc + Hog1PPc_Pbs2PP + Hog1PPc_Pbs2PP_phosphorylated + Hog1PPc_Pbs2 + Hog1PPc_Pbs2_phosphorylated) * to_molecule_number_cytosol | Total number of double phosphorylated Hog1 in the cytosol |
| molecules_Hog1PPn | Hog1PPn*to_molecule_number_nucleus | Total number of double phosphorylated Hog1 in the nucleus |
| starting_molecules_Hog1PPc | init_Hog1PPc*to_molecule_number_cytosol_start | Total number of double phosphorylated Hog1 in the cytosol at the start |
| starting_molecules_Hog1PPn | init_Hog1PPn*to_molecule_number_nucleus_start | Total number of double phosphorylated Hog1 in the nucleus at the start |
| molecules_Hog1P174 | (Hog1P174c + Hog1P174c_Pbs2PP + Hog1P174c_Pbs2PP_phosphorylated + Hog1P174c_Pbs2 + Hog1P174c_Pbs2_phosphorylated ) * to_molecule_number_cytosol + Hog1P174n * to_molecule_number_nucleus | Total number of Hog1 monophosphorylated at Threonine 174 |
| molecules_Hog1P176 | (Hog1P176c + Hog1P176c_Pbs2PP + Hog1P176c_Pbs2PP_phosphorylated + Hog1P176c_Pbs2 + Hog1P176c_Pbs2_phosphorylated) * to_molecule_number_cytosol + Hog1P176n * to_molecule_number_nucleus | Total number of Hog1 monophosphorylated at Tyrosine 176 |
| molecules_Hog1c | (Hog1c + Hog1c_Pbs2PP + Hog1c_Pbs2PP_phosphorylated + Hog1c_Pbs2 + Hog1c_Pbs2_phosphorylated) * to_molecule_number_cytosol | Total number of unphosphorylated Hog1 in the cytosol |
| molecules_Hog1n | Hog1n*to_molecule_number_nucleus | Total number of unphosphorylated Hog1 in the nucleus |
| molecules_Pbs2P | (Pbs2P + Sho1_active_Ste11_Pbs2P + Pbs2P_phosphorylated + Sho1_active_Ste11_Pbs2P_phosphorylated) * to_molecule_number_cytosol | Total number of monophosphorylated Pbs2 |
| starting_molecules_Pbs2P | (init_Pbs2P + init_Pbs2P_phosphorylated)* to_molecule_number_cytosol_start | Total number of monophosphorylated Pbs2 at the start |
| molecules_Ssk2_second_inactive | (Ssk2_phosphorylated_second + Ssk2P_phosphorylated_second) * to_molecule_number_cytosol | Total number of double phosphorylated Ssk2 by feedback |
| molecules_Ssk2_inactive | (Ssk2_phosphorylated + Ssk2P_phosphorylated) * to_molecule_number_cytosol | Total number of mono phosphorylated Ssk2 by feedback |
| total_hog1 | (Hog1c + Hog1P174c + Hog1P176c + Hog1PPc + Hog1c_Pbs2PP + Hog1P174c_Pbs2PP + Hog1P176c_Pbs2PP + Hog1PPc_Pbs2PP + Hog1c_Pbs2_phosphorylated + Hog1P176c_Pbs2_phosphorylated + Hog1P174c_Pbs2_phosphorylated + Hog1PPc_Pbs2_phosphorylated + Hog1c_Pbs2PP_phosphorylated + Hog1P176c_Pbs2PP_phosphorylated + Hog1P174c_Pbs2PP_phosphorylated + Hog1PPc_Pbs2PP_phosphorylated + Hog1c_Pbs2 + Hog1P176c_Pbs2 + Hog1P174c_Pbs2 + Hog1PPc_Pbs2 )*to_molecule_number_cytosol + ( Hog1n + Hog1PPn + Hog1P174n + Hog1P176n)*to_molecule_number_nucleus | Total number of Hog1 of any phosphorylation state in the cell |
| molecules_Gpd1 | Gpd1 * to_molecule_number_cytosol | Total number of Gpd1 |
| molecules_Pbs2_phosphorylated | (Sho1_Pbs2_phosphorylated + Sho1_active_Pbs2_phosphorylated + Sho1_active_Ste11_Pbs2_phosphorylated_inactive + Sho1_active_Ste11_Pbs2_phosphorylated + Sho1_active_Ste11_Pbs2P_phosphorylated + Sho1_active_Ste11_Pbs2PP_phosphorylated + Sho1_active_Ste11_Pbs2_inactive_phosphorylated + Sho1_active_Ste11_Pbs2_phosphorylated + Sho1_active_Ste11_Pbs2P_phosphorylated + Pbs2_phosphorylated + Pbs2P_phosphorylated + Pbs2PP_phosphorylated + Hog1c_Pbs2_phosphorylated + Hog1P176c_Pbs2_phosphorylated + Hog1P174c_Pbs2_phosphorylated + Hog1PPc_Pbs2_phosphorylated + Hog1c_Pbs2PP_phosphorylated + Hog1P176c_Pbs2PP_phosphorylated + Hog1P174c_Pbs2PP_phosphorylated + Hog1PPc_Pbs2PP_phosphorylated) * to_molecule_number_cytosol | Total number of feedback phosphorylated Pbs2 |
| starting_molecules_Gpd1 | init_Gpd1 * to_molecule_number_cytosol_start | Total number of Gpd1 at the start |
| starting_molecules_Pbs2_phosphorylated | (init_Pbs2PP_phosphorylated + init_Pbs2P_phosphorylated + init_Pbs2_phosphorylated + init_Hog1c_Pbs2PP_phosphorylated + init_Hog1P174c_Pbs2PP_phosphorylated + init_Hog1P176c_Pbs2PP_phosphorylated + init_Hog1PPc_Pbs2PP_phosphorylated ) * to_molecule_number_cytosol_start | Total number of feedback phosphorylated Pbs2 at the start |

**Supp Table S6:**

Description of all observables used in the model and the respective experimental data they approximate.

| **Glycerol production** | |
| --- | --- |
| Reaction | Reaction rate |
| -> glycerol | $\left[\text{base\_line}\right]*\text{k\_glyc\_const\_production}$ |
| glycerol -> | $\left[\text{glycerol}\right]*\text{k\_glyc\_exp\_0}$ |
| glycerol -> | $\frac{\left[\text{glycerol}\right]*\text{PI\_t}^{\text{n\_for\_fps1}}*\text{k\_glyc\_exp\_fps1}}{\text{GAMMA}^{\text{n\_for\_fps1}}+\text{PI\_t}^{\text{n\_for\_fps1}}}$ |
| Gpd1-> glycerol + Gpd1 | $\left[\text{Gpd1}\right]\text{*k\_gpd1\_production}$ |
| -> glycerol | $\frac{\left[\text{Hog1PPc}\right]^{\text{n\_hog1\_glyc}}*\text{k\_glyc\_hog1\_dependent\_production}*\text{macia\_factor}}{\text{BETA}^{\text{n\_hog1\_glyc}}\text{+}\left[\text{Hog1PPc}\right]^{\text{n\_hog1\_glyc}}}$ |
| Volume change and salt addition | |
| Reaction | Reaction rate |
| ->V_os_cytosol_ | $-\text{G\_parameter\_cytosol}*(\text{PI}_{t}+\text{PI}_{\text{e}}+\text{PI}_{\text{i}})$ |
| ->V_os_nucleus_ | $-\text{G\_parameter\_nucleus}*(\text{PI}_{\text{t}}+\text{PI}_{\text{e}}+\text{PI}_{\text{i}})$ |
| ->V_os_ | $-\text{G\_parameter\_cell}*(\text{PI}_{\text{t}}+\text{PI}_{\text{e}}+\text{PI}_{\text{i}})$ |
| ->NaCl | $\text{first\_injection}(\frac{1}{\sqrt{2*\pi*\text{time\_for\_perturbation}^{2}}})*e^{\frac{(-(\text{t}-\text{time\_first})^{2}}{2*\text{time\_for\_perturbation}^{2}}}$ |

| **Algebraic pressure parameters** | |
| --- | --- |
| Parameter | Reaction rate |
| PI_t_ | $\left\{\frac{PI_{t}^{0}*(\text{V}_{\text{os}}-\text{V}_{\text{os}}^{\text{PI}_{\text{t}}\text{=0}})}{\begin{matrix} (\text{init\_V}_{\text{os}}-\text{V}_{\text{os}}^{\text{PI}_{\text{t}}\text{=0}}) \\ \text{PI}_{\text{t}}^{\text{below\_threshold}} \end{matrix}}\right\}$ |
| PI_i_ | $2520.3891e-6*(\text{n\_0}+\text{V}_{\text{os\_cytosol}}*\left[\text{glycin}\right])*\frac{1}{\text{V}_{\text{os}}}$ |
| PI_e_ | $\text{PI}_{\text{e\_0}}+\left[\text{NaCl}\right]*\text{w\_parameter}$ |

| Parameter | Parameter value^a^ | Unit | Additional information |
| --- | --- | --- | --- |
| k_glyc_const_production | 10.48 | s^-1^ | Constant basal production of glycerol |
| k_glyc_exp_0 | 3.98e-5 | s^-1^ | Rate of passive glycerol transport to the outside of the cell |
| n_for_fps1 | 9.92 | au |  |
| k_glyc_exp_fps1 | 0.09 | s^-1^ | Rate of glycerol transport to the outside via the Fps1 channel |
| GAMMA | 102.68 | 10^6^ J m^-3^ |  |
| k_gpd1_production | 100.00 | s^-1^ | Rate of glycerol production by Gpd1 |
| n_hog1_glyc | 7.14 | au |  |
| k_glyc_hog1_dependent_production | 43.83 | mM s^-1^ | Rate of glycerol production dependent on Hog1 activity in the cytosol |
| macia_factor | 1 | au | Factor that simulates inhibition of Hog1 activity when put to 0 |
| BETA | 0.03 | au |  |
| G_parameter_cytosol | 0.10 | 10^-24^m^6^J^-1^s^-1^ | Shrinkage factor for cytosol |
| G_parameter_nucleus | 0.02 | 10^-24^m^6^J^-1^s^-1^ | Shrinkage factor for nucleus |
| G_parameter_cell | 0.20 | 10^-24^m^6^J^-1^s^-1^ | Shrinkage factor for cell |
| first_injection | 400000 | μM | NaCl concentration added by a bolus injection |
| time_for_perturbation | 15 | s | Time duration of bolus injection |
| time_first | 2000 | s | Timepoint of NaCl injection |
| ^^ | 55.95 | 10^6^Jm^-3^ | Turgor pressure of unperturbed cell |
| $\text{PI}_{\text{t}}^{\text{below\_threshold}}$ | 0.04 | 10^6^Jm^-3^ | Minimal turgor pressure |
| n_0 | 2.33e8 | 10^-15^μmol | Contribution by osmolarity other than glycerol |
| PI_e_0_ | 208.11 | 10^6^Jm^-3^ | External pressure in unperturbed state |
| w_parameter | 2.98e-4 | 10^6^Jm^-3^M^-1^ | Conversion factor of external salt concentration to external pressure |

| Species | Initial value (unit) | Additional information |
| --- | --- | --- |
| base_line | 1 (μM) | Proxy for the protein machinery needed for basal glycerol production |
| glycerol | 44218 (μM) | Intracellular glycerol |
| Hog1PPc | 0.0105 (mM) | Double phosphorylated Hog1 in the cytosol |
| V_os_cytosol_ | 2677 (10^-16^m^-3^) | Starting volume of the cytosol |
| V_os_nucleus_ | 267.7 (10^-16^m^-3^) | Starting volume of the nucleus |
| V_os_ | 3480 (10^-16^m^-3^) | Starting volume of the complete cell, includes volume that is considered incompressible and is not part of either cytosol or nucleus |
| NaCl | 1 (μM) | Extracellular NaCl concentration |

| **Gpd1 transcription** | |
| --- | --- |
| Reaction | Reaction rate |
| Gene_off -> Gene_on | $\left[\text{Gene\_off}\right]*\text{TRANS\_gene\_on\_background}$ |
| Gene_on -> Gene_off | $\left[\text{Gene\_on}\right]*\text{TRANS\_gene\_off\_background}$ |
| Gene_off + Hog1PPn -> Gene_on_Hog1PPn | $\left[\text{Gene\_off}\right]*\left[\text{Hog1PPn}\right]*\text{TRANS\_gene\_on}$ |
| Gene_on_Hog1PPn -> Gene_off + Hog1PPn | $\left[\text{Gene\_on\_Hog1PPn}\right]\text{*TRANS\_gene\_off}$ |
| Gene_on -> Gene_on_r | $\left[\text{Gene\_on}\right]*\text{TRANS\_gene\_on\_r}$ |
| Gene_on_Hog1PPn -> Gene_on_r_Hog1PPn | $\left[\text{Gene\_on\_Hog1PPn}\right]*\text{TRANS\_gene\_on\_r}$ |
| Gene_on_r -> Gene_on | $\left[\text{Gene\_on\_r}\right]*\text{TRANS\_gene\_off\_r}$ |
| Gene_on_r_Hog1PPn -> Gene_on_Hog1PPn | $\left[\text{Gene\_on\_r\_Hog1PPn}\right]*\text{TRANS\_gene\_off\_r}$ |
| Gene_on_r -> Gene_on_r + mRNA | $\left[\text{Gene\_on\_r}\right]*\text{TRANS\_transcription\_slow}$ |
| Gene_on_r_Hog1PPn -> Gene_on_r_Hog1PPn + mRNA | $\left[\text{Gene\_on\_r\_Hog1PPn}\right]*\text{TRANS\_transcription*macia\_factor}$ |
| mRNA -> | $\left[\text{mRNA}\right]*\text{RNA\_export}$ |
| -> mRNA_cytosol | $\frac{\left[\text{mRNA}\right]*\text{V}_{\text{os\_nucleus}}*\text{RNA\_export}}{\text{V}_{\text{os\_cytosol}}}$ |
| mRNA_cytosol -> mRNA_cytosol + Gpd1_pre | $\left[\text{mRNA\_cytosol}\right]*\text{TRANS\_translation*heximide\_factor}$ |
| mRNA_cytosol -> | $\left[\text{mRNA\_cytosol}\right]*\text{TRANS\_mRNA\_deg}$ |
| Gpd1_pre -> Gpd1 | $\left[\text{Gpd1\_pre}\right]*\text{TRANS\_maturation\_gpd1}$ |
| Gpd1 -> | $\left[\text{Gpd1}\right]*\text{Gpd1\_degradation}$ |

| Parameter | Parameter value^a^ | Unit | Additional information |
| --- | --- | --- | --- |
| TRANS_gene_on_background | 1.4887 | s^-1^ | Slow, basal rate of closed complex formation at promoter for transcription |
| TRANS_gene_off_background | 0.2462 | s^-1^ | Basal rate of disassociation of closed complex from promoter (gene shut off) |
| TRANS_gene_on | 83.4449 | mM^-1^s^-1^ | Rate of priming of promoter for transcription with double phosphorylated Hog1 |
| TRANS_gene_off | 0.1892 | s^-1^ | Rate of disassociation of closed complex from promoter (gene shut off) with double phosphorylated Hog1 bound |
| TRANS_Gene_on_r | 0.0011 | s^-1^ | Rate of formation of open complex |
| TRANS_gene_off_r | 0.0041 | s^-1^ | Rate of closure of open complex |
| TRANS_transcription_slow | 0.0554 | s^-1^ | Slow, basal transcription rate |
| TRANS_transcription | 15.6135 | s^-1^ | Transcription rate with double phosphorylated Hog1 bound |
| macia_factor | 1 | au | Factor that simulates inhibition of Hog1 activity when put to 0 preventing double phosphorylated Hog1 to bind to DNA |
| RNA_export | 55.6801 | s^-1^ | Rate of RNA export into the cytosol |
| V_os_cytosol_ | See above |  | See Volume sub-model |
| V_os_nucleus_ | See above |  | See Volume sub-model |
| TRANS_translation | 1.3533 | s^-1^ | Rate of translation |
| Heximide_factor | 1 | au | Factor that simulates inhibition of translational activity when put to 0 such as by the addition of Heximide |
| TRANS_maturation_gpd1 | 9.7949e-04 | s^-1^ | Rate of protein folding of Gpd1 |
| Gpd1_degradation | 3.7574e-04 | s^-1^ | Rate of degradation of the Gpd1 protein |

| Species | Initial value (unit) | Additional information |
| --- | --- | --- |
| Gene_off | 1.0000e-04 (mM) | Arbitrary concentration value that represents Gene in off state |
| Gene_on | 0 (mM) | Gene in on state |
| Hog1PPn | 0 (mM) | Concentration of double phosphorylated Hog1 in the nucleus |
| Gene_on_r | 0 (mM) | Gene with transcription complex in open position |
| Gene_on_Hog1PPn | 0 (mM) | Double phosphorylated Hog1 bound to Gene in closed position |
| Gene_on_r_Hog1PPn | 0 (mM) | Double phosphorylated Hog1 bound to Gene in open position |
| mRNA | 0.0025 (mM) | Concentration of mRNA in the nucleus |
| mRNA_cytosol | 0 (mM) | Concentration of mRNA in the cytosol |
| Gpd1_pre | 0 (mM) | Concentration of unfolded Gpd1 protein |
| Gpd1_a_ | 0.05 (mM) | Concentration of folded Gpd1 |

_a_ Total amount of Gpd1 in the cell: 807 molecules^23^

**Supp Table S7: Volume module**

Description of the reactions, reaction rates, parameter values and species simulating glycerol concentration and volume changes in our model (volume module).

| **Sho1 sub-branch** | |
| --- | --- |
| Reaction | Reaction rate |
| Sho1 -> Sho1_active | $\frac{\left[\text{Sho1}\right]*\text{SHO1\_sho1\_activation*(PI}_{\text{t\_for\_sho1}})^{\text{n\_sho1}}}{\text{Sho1\_sigmoid\_parameter}+\text{(PI}_{\text{t\_for\_sho1}})^{\text{n\_sho1}}}*\text{SHO1\_shut\_off\_parameter}$ |
| Sho1_active -> Sho1 | $\left[\text{Sho1\_active}\right]*\text{SHO1\_sho1\_dephosphorylation}$ |
| Sho1 + Pbs2 -> Sho1_Pbs2 | $\left[\text{Sho1}\right]*\left[\text{Pbs2}\right]*\text{SHO1\_sho1\_pbs2\_association}$ |
| Sho1_Pbs2 -> Sho1 + Pbs2 | $\left[\text{Sho1\_Pbs2}\right]*\text{SHO1\_sho1\_pbs2\_break\_up}$ |
| Sho1_Pbs2 -> Sho1_active_Pbs2 | $\frac{\left[\text{Sho1\_Pbs2}\right]*\text{SHO1\_sho1\_activation*(PI}_{\text{t\_for\_sho1}})^{\text{n\_sho1}}}{\text{Sho1\_sigmoid\_parameter}+\text{(PI}_{\text{t\_for\_sho1}})^{\text{n\_sho1}}}*\text{SHO1\_shut\_off\_parameter}$ |
| Sho1_active + Pbs2 -> Sho1_active_Pbs2 | $\left[\text{Sho1\_active}\right]*\left[\text{Pbs2}\right]*\text{SHO1\_sho1\_pbs2\_association}$ |
| Sho1_active_Pbs2 -> Sho1_active + Pbs2 | $\left[\text{Sho1\_active\_Pbs2}\right]*\text{SHO1\_sho1\_pbs2\_break\_up}$ |
| 2 Ste11 + Ste50 -> Ste11_Ste50 | $\left[\text{Ste11}\right]^{2}*\left[\text{Ste50}\right]*\text{Ste11\_ste50\_formation}$ |
| Ste11_Ste50 -> 2 Ste11 + Ste50 | $\left[\text{Ste11\_Ste50}\right]*\text{Ste11\_ste50\_break\_up}$ |
| Sho1_active_Pbs2 + Ste11_Ste50 -> Sho1_active_Ste11_Pbs2_inactive | $\left[\text{Sho1\_active\_Pbs2}\right]*\left[\text{Ste11\_50}\right]*\text{SHO1\_binding\_of\_ste11}$ |
| Sho1_active_Ste11_Pbs2_inactive + Ste20 -> Sho1_active_Ste11_Pbs2 + Ste20 | $\left[\text{Sho1\_active\_Ste11\_Pbs2\_inactive}\right]*\left[\text{Ste20}\right]*\text{k\_ste11\_phosphorylation\_by\_ste20}$ |
| Sho1_active + Ste11_Ste50 -> Sho1_active_Ste11 | $\left[\text{Sho1\_active}\right]*\left[\text{Ste11\_50}\right]*\text{SHO1\_binding\_of\_ste11}$ |
| Sho1_active_Ste11 + Ste20 -> Sho1_active_Ste11PP + Ste20 | $\left[\text{Sho1\_active\_Ste11}\right]*\left[\text{Ste20}\right]*\text{k\_ste11\_phosphorylation\_by\_ste20}$ |
| Sho1_active_Ste11PP + Pbs2 -> Sho1_active_Ste11_Pbs2 | $\left[\text{Sho1\_active\_Ste11PP}\right]*\left[\text{Pbs2}\right]*\text{SHO1\_sho1\_pbs2\_association}$ |
| Sho1_active_Ste11_Pbs2 -> Sho1_active_Ste11PP + Pbs2 | $\left[\text{Sho1\_active\_Ste11\_Pbs2}\right]*\text{SHO1\_sho1\_pbs2\_break\_up}$ |
| Sho1_active_Ste11_Pbs2 -> Sho1_active_Ste11_Pbs2P | $\left[\text{Sho1\_active\_Ste11\_Pbs2}\right]*\text{SHO1\_phosphorylation\_of\_pbs2}$ |
| Sho1_active_Ste11_Pbs2P -> Sho1_active_Ste11PP + Pbs2P | $\left[\text{Sho1\_active\_Ste11\_Pbs2P}\right]*\text{SHO1\_sho1\_pbs2\_break\_up}$ |
| Sho1_active_Ste11_Pbs2P -> Sho1_active_Ste11_Pbs2PP | $\left[\text{Sho1\_active\_Ste11\_Pbs2P}\right]*\text{SHO1\_phosphorylation\_of\_pbs2}$ |
| Sho1_active_Ste11PP + Pbs2P -> Sho1_active_Ste11_Pbs2P | $\left[\text{Sho1\_active\_Ste11PP}\right]*\left[\text{Pbs2P}\right]*\text{SHO1\_sho1\_pbs2\_association}$ |
| Sho1_active_Ste11_Pbs2PP -> Sho1_active_Ste11PP + Pbs2PP | $\left[\text{Sho1\_active\_Ste11\_Pbs2PP}\right]*\text{SHO1\_sho1\_pbs2\_break\_up\_duo}$ |
| Sho1_active_Ste11PP -> Sho1_active + Ste11P | $\left[\text{Sho1\_active\_Ste11PP}\right]*\text{SHO1\_break\_up\_of\_sho1\_ste11}$ |
| Ste11PP -> Ste11_Ste50 | $\left[\text{Ste11PP}\right]*\text{Ste11\_dephosphorylation}$ |

| Parameter | Parameter value | Unit | Additional information |
| --- | --- | --- | --- |
| SHO1_sho1_activation | 1.2221e+06 | au |  |
| n_sho1 | 9.7701 | au |  |
| PI_t_for_sho1_ | 1/PI_t_ |  | See **Volume Sub-model** for details of PI_t_ |
| Sho1_sigmoid_parameter | 1.1577e-04 | au |  |
| SHO1_shut_off_parameter | 1 | au | Switch parameter to enable/disable Sho1 sub-branch |
| SHO1_sho1_dephosphorylation | 0.0184 | s^-1^ | Dephosphorylation and thus deactivation of activated Sho1 |
| SHO1_sho1_pbs2_association | 7.7446 | mM^-1^s^-1^ | Association of Pbs2 to Sho1 |
| SHO1_sho1_pbs2_break_up | 0.5213 | s^-1^ | Disassociation of Pbs2 from Sho1 |
| Ste11_ste50_formation | 4.0738 | mM^-1^s^-1^ | Association of two Ste11 molecules and one Ste50 |
| Ste11_ste50_break_up | 0.8730 | s^-1^ | Disassociation of Ste11-Ste50 complex |
| SHO1_binding_of_ste11 | 1.4880e+06 | mM^-1^s^-1^ | Association of Sho1 and Ste11-Ste50 complex |
| k_ste11_phosphorylation_by_ste20 | 5.1928 | mM^-1^s^-1^ | Phosphorylation of Ste11 by Ste20 |
| SHO1_phosphorylation_of_pbs2 | 4.8295e+06 | s^-1^ | Phosphorylation of one Pbs2 phosphosite by activated Ste11 in big Sho1 complex |
| SHO1_sho1_pbs2_break_up_duo | 8.3426e+09 | s^-1^ | Disassociation of double phosphorylated Pbs2 and big Sho1 complex |
| SHO1_break_up_of_sho1_ste11 | 88.9406 | s^-1^ | Disassociation of Sho1 and Ste11-Ste50 complex |
| Ste11_dephosphorylation | 435.6122 | s^-1^ | Dephosphorylation of Ste11 by unspecified phosphatase |

| Species | Initial value (unit) | Additional information |
| --- | --- | --- |
| Sho1_a_ | 0.1334 (mM) | Free Sho1 |
| Sho1_active | 0 (mM) | Sho1 activated upon salt stress |
| Pbs2_b_ | 0.1138 (mM) | Free Pbs2 |
| Sho1_Pbs2 | 0.0102 (mM) | Sho1 bound to Pbs2 |
| Sho1_active_Pbs2 | 0 (mM) | Sho1 activated upon salt stress bound to Pbs2 |
| Ste11_c_ | 0.0421 (mM) | Free Ste11 |
| Ste50_d_ | 0.0263 (mM) | Free Ste50 |
| Ste11_Ste50 | 0 (mM) | Complex consisting of two molecules Ste11 and one Ste50 |
| Sho1_active_Ste11_Pbs2_inactive | 0 (mM) | Complex of activated Sho1, inactive Ste11-Ste50 complex and Pbs2 |
| Ste20_e_ | 0.0161 (mM) | Free Ste20 |
| Sho1_active_Ste11_Pbs2 | 0 (mM) | Complex of activated Sho1, activated Ste11-Ste50 complex and Pbs2 |
| Sho1_active_Ste11 | 0 (mM) | Complex of activated Sho1 and inactive Ste11-Ste50 complex |
| Sho1_active_Ste11PP | 0 (mM) | Complex of activated Sho1 and activated Ste11-Ste50 complex |
| Sho1_active_Ste11_Pbs2P | 0 (mM) | Complex of activated Sho1, activated Ste11-Ste50 complex and monophosphorylated Pbs2 |
| Pbs2P | 0.0029 (mM) | Free monophosphorylated Pbs2 |
| Sho1_active_Ste11_Pbs2PP | 0 (mM) | Complex of activated Sho1, activated Ste11-Ste50 complex and double phosphorylated Pbs2 |
| Pbs2PP | 0 (mM) | Free double phosphorylated Pbs2 |
| Ste11PP | 0 (mM) | Free activated Ste11-Ste50 complex |

_a_ Total amount of Sho1 in the cell: 2330 molecules^23^

_b_ Total amount of Pbs2 in the cell: 2160 molecules^23^

_c_ Total amount of Ste11 in the cell: 736 molecules^23^

_d_ Total amount of Ste50 in the cell: 1670 molecules^23^

_e_ Total amount of Ste20 in the cell: 259 molecules^23^

**Supp Table S8: Sho1 sub-branch**

Description of the reactions, reaction rates, parameter values and species of the sub-model simulating the Sho1 sub-branch including activation of Pbs2.

| **Sln1 sub-branch** | |
| --- | --- |
| Reaction | Reaction rate |
| Sln1 -> Sln1P | $\left[\text{Sln1}\right]*\text{PI}_{\text{t}}*\text{k\_sln1\_autophosphorylation}$ |
| Sln1P + Ypd1-> Sln1 + Ypd1P | $\left[\text{Sln1P}\right]*\left[\text{Ypd1}\right]*\text{k\_sln1p\_to\_ypd1\_phosphotransfer}$ |
| Sln1 + Ypd1P -> Sln1P + Ypd1 | $\left[\text{Sln1}\right]*\left[\text{Ypd1P}\right]*\text{k\_ypd1p\_to\_sln1\_phosphotransfer}$ |
| Ypd1P + Ssk1 -> Ypd1 + Ssk1P | $\left[\text{Ypd1P}\right]*\left[\text{Ssk1}\right]*\text{k\_ypd1p\_to\_ssk1\_phosphotransfer}$ |
| Ssk1P -> Ssk1 | $\left[\text{Ssk1P}\right]*\text{k\_ssk1P\_dephosphorylation}$ |
| Ssk2 + Ssk1 -> Ssk2P + Ssk1 | $\left[\text{Ssk2}\right]*\left[\text{Ssk1}\right]*\text{k\_ssk2\_autophosphorylation\_assisted\_by\_ssk1*Sln1\_shut\_off\_parameter}$ |
| Ssk2P -> Ssk2 | $\left[\text{Ssk2P}\right]*\text{k\_ssk2\_dephosphorylation}$ |
| Ssk2P + Pbs2 -> Pbs2P + Ssk2P | $\left[\text{Ssk2P}\right]*\left[\text{Pbs2}\right]*\text{k\_pbs2\_phosphorylation\_by\_ssk2P\_mono}$ |
| Pbs2P + Ssk2P -> Pbs2PP + Ssk2P | $\left[\text{Ssk2P}\right]*\left[\text{Pbs2P}\right]*\text{k\_pbs2\_phosphorylation\_by\_ssk2P\_duo}$ |
| Pbs2PP -> Pbs2P | $\left[\text{Pbs2PP}\right]*\text{k\_pbs2PP\_dephosphorylation\_duo}$ |
| Pbs2P -> Pbs2 | $\left[\text{Pbs2P}\right]*\text{k\_pbs2P\_dephosphorylation\_mono}$ |

| Parameter | Parameter value | Unit | Additional information |
| --- | --- | --- | --- |
| PI_t_ | variable |  | Variable turgor pressure. See Volume sub-model |
| k_sln1_autophosphorylation | 0.01 | s^-1^ | Basal autophosphorylation of Sln1 |
| k_sln1p_to_ypd1_phosphotransfer | 51.92 | mM^-1^s^-1^ | Transfer of phosphate from Sln1 to Ypd1 |
| k_ypd1P_to_sln1_phosphotransfer | 418.02 | mM^-1^s^-1^ | Transfer of phosphate from Ypd1 to Sln1 |
| k_ypd1p_to_ssk1_phosphotransfer | 999.77 | mM^-1^s^-1^ | Transfer of phosphate from Ypd1 to Ssk1 |
| k_ssk1P_dephosphorylation | 0.23 | s^-1^ | Dephosphorylation of Ssk1 by unspecified phosphatase |
| k_ssk2_autophosphorylation_assisted_by_ssk1 | 28.61 | mM^-1^s^-1^ | Autophosphorylation of Ssk2 induced by association to Ssk1 |
| k_ssk2_dephosphorylation | 1.38 | s^-1^ | Dephosphorylation of Ssk2 by unspecified phosphatase |
| k_pbs2_phosphorylation_by_ssk2P_mono | 448.75 | mM^-1^s^-1^ | Phosphorylation of Pbs2 by phosphorylated Ssk2 |
| k_pbs2p_phosphorylation_by_ssk2P_duo | 434.41 | mM^-1^s^-1^ | Phosphorylation of Pbs2P by phosphorylated Ssk2 |
| k_pbs2PP_dephosphorylation_duo | 0.83 | s^-1^ | Dephosphorylation of Pbs2PP by unspecified phosphatase |
| k_pbs2P_dephosphorylation_mono | 1.08 | s^-1^ | Dephosphorylation of Pbs2P by unspecified phosphatase |

| **Pbs2 feedback** | |
| --- | --- |
| Pbs2 + Hog1PPc -> Pbs2_phosphorylated + Hog1PPc | $\left[\text{Pbs2}\right]*\left[\text{Hog1PPc}\right]*\text{k\_pbs2\_phosphorylation\_by\_hog1\_feedback*switch\_pbs2\_phosphorylation\_feedback*macia\_factor}$ |
| Pbs2P + Hog1PPc -> Pbs2P_phosphorylated + Hog1PPc | $\left[\text{Pbs2P}\right]*\left[\text{Hog1PPc}\right]*\text{k\_pbs2P\_phosphorylation\_by\_hog1\_feedback*switch\_pbs2\_phosphorylation\_feedback*macia\_factor}$ |
| Pbs2PP + Hog1PPc -> Pbs2PP_phosphorylated + Hog1PP | $\left[\text{Pbs2PP}\right]*\left[\text{Hog1PPc}\right]*\text{k\_pbs2PP\_phosphorylation\_by\_hog1\_feedback*switch\_pbs2\_phosphorylation\_feedback*macia\_factor}$ |
| Pbs2_phosphorylated -> Pbs2 | $\left[\text{Pbs2\_phosphorylated}\right]*\text{k\_pbs2\_phosphorylated\_dephosphorylation\_feedback*switch\_pbs2\_phosphorylation\_feedback}$ |
| Pbs2P_phosphorylated -> Pbs2P | $\left[\text{Pbs2P\_phosphorylated}\right]*\text{k\_pbs2\_phosphorylated\_dephosphorylation\_feedback*switch\_pbs2\_phosphorylation\_feedback}$ |
| Pbs2PP_phosphorylated -> Pbs2PP | $\left[\text{Pbs2PP\_phosphorylated}\right]*\text{k\_pbs2\_phosphorylated\_dephosphorylation\_feedback*switch\_pbs2\_phosphorylation\_feedback}$ |
| Pbs2PP_phosphorylated -> Pbs2P_phosphorylated | $\left[\text{Pbs2PP\_phosphorylated}\right]*\text{k\_pbs2PP\_dephosphorylation\_duo}$ |
| Pbs2P_phosphorylated -> Pbs2_phosphorylated | $\left[\text{Pbs2P\_phosphorylated}\right]*\text{k\_pbs2P\_dephosphorylation\_mono}$ |

| Parameter | Parameter value | Unit | Additional Information |
| --- | --- | --- | --- |
| k_pbs2_phosphorylation_by_hog1_feedback | 6.07 | mM^-1^s^-1^ | Feedback phosphorylation of Pbs2 by double phosphorylated Hog1 |
| k_pbs2P_phosphorylation_by_hog1_feedback | 0.03 | mM^-1^s^-1^ | Feedback phosphorylation of Pbs2P by double phosphorylated Hog1 |
| k_pbs2PP_phosphorylation_by_hog1_feedback | 149.00 | mM^-1^s^-1^ | Feedback phosphorylation of Pbs2PP by double phosphorylated Hog1 |
| k_pbs2_phosphorylated_dephosphorylation_feedback | 0.38 | s^-1^ | Dephosphorylation of feedback phosphorylation by unspecified phosphatase |
| k_pbs2PP_dephosphorylation_duo | 0.83 | s^-1^ | Dephosphorylation of Pbs2PP by unspecified phosphatase |
| k_pbs2P_dephosphorylation_mono | 1.08 | s^-1^ | Dephosphorylation of Pbs2P by unspecified phosphatase |
| switch_pbs2_phosphorylation_feedback | 1 | au | Switch parameter to enable/disable pbs2 feedback |
| macia_factor | 1 | au | Factor that simulates inhibition of Hog1 activity when put to 0 |

| **Ssk2 feedback** | |
| --- | --- |
| Ssk2 + Hog1PPc -> Ssk2_phosphorylated + Hog1PPc | $\left[\text{Ssk2}\right]*\left[\text{Hog1PPc}\right]*\text{k\_ssk2\_phosphorylation\_by\_hog1ppc*switch\_ssk2\_phosphorylation\_feedback*macia\_factor}$ |
| Ssk2_phosphorylated + Hog1PPc -> Ssk2_phosphorylated_second + Hog1PPc | $\begin{matrix} \left[\text{Ssk2\_phosphorylated}\right]*\left[\text{Hog1PPc}\right]*\text{k\_ssk2\_phosphorylated\_phosphorylation\_by\_hog1ppc} \\ \text{*switch\_ssk2\_phosphorylation\_feedback*macia\_factor} \end{matrix}$ |
| Ssk2P + Hog1PPc -> Ssk2P_phosphorylated + Hog1PPc | $\left[\text{Ssk2P}\right]*\left[\text{Hog1PPc}\right]*\text{k\_ssk2\_phosphorylation\_by\_hog1ppc*switch\_ssk2\_phosphorylation\_feedback*macia\_factor}$ |
| Ssk2P_phosphorylated + Hog1PPc -> Ssk2P_phosphorylated_second + Hog1PPc | $\begin{matrix} \left[\text{Ssk2P\_phosphorylated}\right]*\left[\text{Hog1PPc}\right]*\text{k\_ssk2\_phosphorylated\_phosphorylation\_by\_hog1ppc} \\ \text{*switch\_ssk2\_phosphorylation\_feedback*macia\_factor} \end{matrix}$ |
| Ssk2_phosphorylated_second -> Ssk2_phosphorylated | $\left[\text{Ssk2\_phosphorylated\_second}\right]*\text{k\_ssk2\_phosphorylated\_second\_dephosphorylation}$ |
| Ssk2_phosphorylated -> Ssk2 | $\left[\text{Ssk2\_phosphorylated}\right]*\text{k\_ssk2\_phosphorylated\_dephosphorylation}$ |
| Ssk2P_phosphorylated_second -> Ssk2P_phosphorylated | $\left[\text{Ssk2P\_phosphorylated\_second}\right]*\text{k\_ssk2\_phosphorylated\_second\_dephosphorylation}$ |
| Ssk2P_phosphorylated -> Ssk2P | $\left[\text{Ssk2P\_phosphorylated}\right]*\text{k\_ssk2\_phosphorylated\_dephosphorylation}$ |
| Ssk2P_phosphorylated + Pbs2 -> Pbs2P + Ssk2P_phosphorylated | $\left[\text{Ssk2P\_phosphorylated}\right]*\left[\text{Pbs2}\right]*\text{k\_pbs2\_phosphorylation\_by\_ssk2P\_mono*feedback\_ssk2\_mono}$ |
| Pbs2P + Ssk2P_phosphorylated -> Pbs2PP + Ssk2P_phosphorylated | $\left[\text{Ssk2P\_phosphorylated}\right]*\left[\text{Pbs2P}\right]*\text{k\_pbs2\_phosphorylation\_by\_ssk2P\_duo*feedback\_ssk2\_duo}$ |
| Ssk2P_phosphorylated_second + Pbs2 -> Pbs2P + Ssk2P_phosphorylated_second | $\left[\text{Ssk2P\_phosphorylated\_second}\right]*\left[\text{Pbs2}\right]*\text{k\_pbs2\_phosphorylation\_by\_ssk2P\_mono*feedback\_ssk2\_second\_mono}$ |
| Pbs2P + Ssk2P_phosphorylated_second -> Pbs2PP + Ssk2P_phosphorylated_second | $\left[\text{Ssk2P\_phosphorylated\_second}\right]*\left[\text{Pbs2P}\right]*\text{k\_pbs2\_phosphorylation\_by\_ssk2P\_duo*feedback\_ssk2\_second\_duo}$ |
| Ssk2P_phosphorylated + Pbs2_phosphorylated -> Pbs2P_phosphorylated + Ssk2P_phosphorylated | $\left[\text{Ssk2P\_phosphorylated}\right]*\left[\text{Pbs2\_phosphorylated}\right]*\text{k\_pbs2\_phosphorylation\_by\_ssk2P\_mono*feedback\_ssk2\_mono}$ |
| Pbs2P_phosphorylated + Ssk2P_phosphorylated -> Pbs2PP_phosphorylated + Ssk2P_phosphorylated | $\left[\text{Ssk2P\_phosphorylated}\right]*\left[\text{Pbs2P\_phosphorylated}\right]*\text{k\_pbs2\_phosphorylation\_by\_ssk2P\_duo*feedback\_ssk2\_duo}$ |
| Ssk2P_phosphorylated_second + Pbs2_phosphorylated -> Pbs2P_phosphorylated + Ssk2P_phosphorylated_second | $\left[\text{Ssk2P\_phosphorylated\_second}\right]*\left[\text{Pbs2\_phosphorylated}\right]*\text{k\_pbs2\_phosphorylation\_by\_ssk2P\_mono*feedback\_ssk2\_second\_mono}$ |
| Pbs2P_phosphorylated + Ssk2P_phosphorylated_second -> Pbs2PP_phosphorylated + Ssk2P_phosphorylated_second | $\left[\text{Ssk2P\_phosphorylated\_second}\right]*\left[\text{Pbs2P\_phosphorylated}\right]*\text{k\_pbs2\_phosphorylation\_by\_ssk2P\_duo*feedback\_ssk2\_second\_duo}$ |
| **Unchanged reactions with Pbs2/Ssk2 feedback** | |
| Ssk2P + Pbs2_phosphorylated -> Pbs2P_phosphorylated + Ssk2P | $\left[\text{Ssk2P}\right]*\left[\text{Pbs2\_phosphorylated}\right]*\text{k\_pbs2\_phosphorylation\_by\_ssk2P\_mono}$ |
| Pbs2P_phosphorylated + Ssk2P -> Pbs2PP_phosphorylated + Ssk2P | $\left[\text{Ssk2P}\right]*\left[\text{Pbs2P\_phosphorylated}\right]*\text{k\_pbs2\_phosphorylation\_by\_ssk2P\_duo}$ |

| Parameter | Parameter value | Unit | Additional Information |
| --- | --- | --- | --- |
| switch_ssk2_phosphorylation_feedback | 1 | au | Switch parameter to enable/disable feedback on Ssk2 |
| macia_factor | 1 | au | Factor that simulates inhibition of Hog1 activity when put to 0 |
| k_ssk2_phosphorylation_by_hog1ppc | 0.01 | mM^-1^s^-1^ | Feedback phosphorylation of Ssk2 by double phosphorylated Hog1 |
| k_ssk2_phosphorylated_phosphorylation_by_hog1ppc | 13.39 | mM^-1^s^-1^ | Feedback phosphorylation of phosphorylated Ssk2 by double phosphorylated Hog1 |
| k_ssk2_phosphorylated_second_dephosphorylation | 457.61 | s^-1^ | Dephosphorylation of feedback induced double phosphorylated Ssk2 by unspecified phosphatase |
| k_ssk2_phosphorylated_dephosphorylation | 5.02E-05 | s^-1^ | Dephosphorylation of feedback induced phosphorylated Ssk2 by unspecified phosphatase |
| k_pbs2_phosphorylation_by_ssk2P_mono | 448.75 | mM^-1^s^-1^ | Phosphorylation of Pbs2 by phosphorylated Ssk2 |
| k_pbs2p_phosphorylation_by_ssk2P_duo | 434.41 | mM^-1^s^-1^ | Phosphorylation of Pbs2P by phosphorylated Ssk2 |
| feedback_ssk2_mono | 1.92E-05 | mM^-1^s^-1^ | Multiplicative decrease of phosphorylation of Pbs2 by feedback induced monophosphorylated Ssk2 |
| feedback_ssk2_duo | 1.17E-05 | mM^-1^s^-1^ | Multiplicative decrease of phosphorylation of Pbs2P by feedback induced monophosphorylated Ssk2 |
| feedback_ssk2_second_mono | 0.84 | mM^-1^s^-1^ | Multiplicative decrease of phosphorylation of Pbs2 by feedback induced double phosphorylated Ssk2 |
| feedback_ssk2_second_duo | 7.85e-4 | mM^-1^s^-1^ | Multiplicative decrease of phosphorylation of Pbs2P by feedback induced double phosphorylated Ssk2 |

| Species | Initial value (unit) | Additional information |
| --- | --- | --- |
| Sln1_a_ | 0.0003 (mM) | Free Sln1 |
| Sln1P | 0.0407 (mM) | Free phosphorylated and activated Sln1 |
| Ypd1_b_ | 0.3926 (mM) | Free Ypd1 |
| Ypd1P | 0 (mM) | Free phosphorylated Ypd1 |
| Ssk1_c_ | 0 (mM) | Free Ssk1 activated |
| Ssk1P | 0.0744 (mM) | Free Ssk1 inactivated by phosphorylation |
| Ssk2_d_ | 0.0032 (mM) | Free Ssk2 inactive |
| Ssk2P | 0 (mM) | Free Ssk2 inactivated by phosphorylation |
| Ssk2_phosphorylated | 0.0084 (mM) | Free monophosphorylated, inactive Ssk2 |
| Ssk2_phosphorylated_second | 1.0720e-06 (mM) | Free double phosphorylated, inactive Ssk2 |
| Ssk2P_phosphorylated | 0.0019 (mM) | Free monophosphorylated, active Ssk2 |
| Ssk2P_phosphorylated_second | 1.0720e-06 (mM) | Free double phosphorylated, active Ssk2 |
| Pbs2_e_ | 0.1138 (mM) | Free Pbs2 |
| Pbs2P | 0.0029 (mM) | Free monophosphorylated Pbs2 |
| Pbs2PP | 0 (mM) | Free double activated Pbs2 |
| Pbs2_phosphorylated | 0.01 (mM) | Free Pbs2 with feedback phosphorylation |
| Pbs2P_phosphorylated | 3.4293e-05 (mM) | Free monophosphorylated Pbs2 with feedback phosphorylation |
| Pbs2PP_phosphorylated | 1.26e-6 (mM) | Free double activated Pbs2 with feedback phosphorylation |

_a_ Total amount of Sln1 in the cell: 656 molecules^23^

_b_ Total amount of Ypd1 in the cell: 6330 molecules^23^

_c_ Total amount of Ssk1 in the cell: 1200 molecules^23^

_d_ Total amount of Ssk2 in the cell: 217 molecules^23^

_e_ Total amount of Pbs2 in the cell: 2160 molecules^23^

**Supp Table S9: Sln1 sub-branch**

Description of the reactions, reaction rates, parameter values, and species of the sub-model simulating the Sln1 sub-branch including feedback mechanisms on Ssk2 and Pbs2.

| **Hog1 shuttling** | |
| --- | --- |
| Reaction | Reaction rate |
| Hog1c -> | $\left[\text{Hog1c}\right]*\text{k\_hog1c\_import}$ |
| Hog1n -> | $\left[\text{Hog1n}\right]*\text{k\_hog1n\_export}$ |
| -> Hog1n | $\frac{\left[\text{Hog1c}\right]*\text{V}_{\text{os\_cytosol}}*\text{k\_hog1c\_import}}{\text{V}_{\text{os\_nucleus}}}$ |
| -> Hog1c | $\frac{\left[\text{Hog1n}\right]*\text{V}_{\text{os\_nucleus}}*\text{k\_hog1n\_export}}{\text{V}_{\text{os\_cytosol}}}$ |
| Hog1PPc -> | $\left[\text{Hog1PPc}\right]*\text{k\_hog1PPc\_imp}*\text{shuttling}$ |
| Hog1PPn -> | $\left[\text{Hog1PPn}\right]*\text{k\_hog1PPn\_exp}*\text{shuttling}$ |
| -> Hog1PPn | $\frac{\left[\text{Hog1PPc}\right]*\text{V}_{\text{os\_cytosol}}*\text{k\_hog1PPc\_imp}*\text{shuttling}}{\text{V}_{\text{os\_nucleus}}}$ |
| -> Hog1PPc | $\frac{\left[\text{Hog1PPn}\right]*\text{V}_{\text{os\_nucleus}}*\text{k\_hog1PPn\_exp}*\text{shuttling}}{\text{V}_{\text{os\_cytosol}}}$ |
| Hog1P176c -> | $\left[\text{Hog1P176c}\right]*\text{k\_hog1c\_import}$ |
| Hog1P176n -> | $\left[\text{Hog1P176n}\right]*\text{k\_hog1n\_export}$ |
| -> Hog1P176n | $\frac{\left[\text{Hog1P176c}\right]*\text{V}_{\text{os\_cytosol}}*\text{k\_hog1c\_import}}{\text{V}_{\text{os\_nucleus}}}$ |
| -> Hog1P176c | $\frac{\left[\text{Hog1P176n}\right]*\text{V}_{\text{os\_nucleus}}*\text{k\_hog1n\_export}}{\text{V}_{\text{os\_cytosol}}}$ |
| Hog1P174c -> | $\left[\text{Hog1P174c}\right]*\text{k\_hog1c\_import}$ |
| Hog1P174n -> | $\left[\text{Hog1P174n}\right]*\text{k\_hog1n\_export}$ |
| -> Hog1P174n | $\frac{\left[\text{Hog1P174c}\right]*\text{V}_{\text{os\_cytosol}}*\text{k\_hog1c\_import}}{\text{V}_{\text{os\_nucleus}}}$ |
| -> Hog1P174c | $\frac{\left[\text{Hog1P174n}\right]*\text{V}_{\text{os\_nucleus}}*\text{k\_hog1n\_export}}{\text{V}_{\text{os\_cytosol}}}$ |

| Parameter | Parameter value^a^ | Unit | Additional information |
| --- | --- | --- | --- |
| k_hog1c_import | 0.1346 | s^-1^ | Passive, basal import of non-phosphorylated and mono-phosphorylated Hog1 |
| k_hog1n_export | 0.8902 | s^-1^ | Passive, basal export of non-phosphorylated and mono-phosphorylated Hog1 |
| V_os_cytosol_ | var |  | Variable parameter of the changing cytosol volume depending on pressure: see Supp Volume sub-model |
| V_os_nucleus_ | var |  | Variable parameter of the changing nuclear volume depending on pressure: see Supp Volume sub-model |
| K_hog1PPc_imp | 0.0231 | s^-1^ | Active import of double phosphorylated Hog1 |
| K_hog1PPn_exp | 1.3125e-6 | s^-1^ | Active export of double phosphorylated Hog1 is basically non-existent |
| shuttling | 1 | dimensionless | Auxilliary parameter to switch shuttling on or off |

| **Hog1 phosphorylation mixed mechanism** | |
| --- | --- |
| Hog1c + Pbs2PP -> Hog1c_Pbs2PP | $\left[\text{Hog1c}\right]*\left[\text{Pbs2PP}\right]*\text{HOG1\_pbs2pp\_formation}$ |
| Hog1c_Pbs2PP -> Hog1c + Pbs2PP | $\left[\text{Hog1c\_Pbs2PP}\right]*\text{HOG1\_pbs2PP\_break\_up}$ |
| Hog1c_Pbs2PP -> Hog1P174c_Pbs2PP | $\left[\text{Hog1c\_Pbs2PP}\right]*\text{HOG1\_pbs2PP\_phosphorylation\_174\_mono}$ |
| Hog1c_Pbs2PP -> Hog1P176c_Pbs2PP | $\left[\text{Hog1c\_Pbs2PP}\right]*\text{HOG1\_pbs2PP\_phosphorylation\_176\_mono}$ |
| Hog1P176c + Pbs2PP -> Hog1P176c_Pbs2PP | $\left[\text{Hog1P176c}\right]*\left[\text{Pbs2PP}\right]*\text{HOG1\_pbs2pp\_formation\_P}$ |
| Hog1P174c + Pbs2PP -> Hog1P174c_Pbs2PP | $\left[\text{Hog1P174c}\right]*\left[\text{Pbs2PP}\right]*\text{HOG1\_pbs2pp\_formation\_P}$ |
| Hog1P174c_Pbs2PP -> Hog1P174c + Pbs2PP | $\left[\text{Hog1P174c\_Pbs2PP}\right]*\text{HOG1\_pbs2PP\_break\_up\_P}$ |
| Hog1P176c_Pbs2PP -> Hog1P176c + Pbs2PP | $\left[\text{Hog1P176c\_Pbs2PP}\right]*\text{HOG1\_pbs2PP\_break\_up\_P}$ |
| Hog1P174c_Pbs2PP -> Hog1PPc_Pbs2PP | $\left[\text{Hog1P174c\_Pbs2PP}\right]*\text{HOG1\_pbs2PP\_phosphorylation\_176\_duo}$ |
| Hog1P176c_Pbs2PP -> Hog1PPc_Pbs2PP | $\left[\text{Hog1P176c\_Pbs2PP}\right]*\text{HOG1\_pbs2PP\_phosphorylation\_174\_duo}$ |
| Hog1PPc + Pbs2PP -> Hog1PPc_Pbs2PP | $\left[\text{Hog1PPc}\right]*\left[\text{Pbs2PP}\right]*\text{HOG1\_pbs2pp\_formation\_PP}$ |
| Hog1PPc_Pbs2PP -> Hog1PPc + Pbs2PP | $\left[\text{Hog1PPc\_Pbs2PP}\right]*\text{HOG1\_pbs2PP\_break\_up\_PP}$ |
| Hog1c + Pbs2PP_phosphorylated -> Hog1c_Pbs2PP_phosphorylated | $\left[\text{Hog1c}\right]*\left[\text{Pbs2PP\_phosphorylated}\right]*\text{HOG1\_pbs2pp\_formation}*\text{feedback\_pbs2\_formation}$ |
| Hog1c_Pbs2PP_phosphorylated -> Hog1c + Pbs2PP_phosphorylated | $\left[\text{Hog1c\_Pbs2PP\_phosphorylated}\right]*\text{HOG1\_pbs2PP\_break\_up}*\text{feedback\_pbs2\_break\_up}$ |
| Hog1c_Pbs2PP_phosphorylated -> Hog1P174c_Pbs2PP_phosphorylated | $\left[\text{Hog1c\_Pbs2PP\_phosphorylated}\right]*\text{HOG1\_pbs2PP\_phosphorylation\_174\_mono}*\text{feedback\_pbs2\_mono}$ |
| Hog1c_Pbs2PP_phosphorylated -> Hog1P176c_Pbs2PP_phosphorylated | $\left[\text{Hog1c\_Pbs2PP\_phosphorylated}\right]*\text{HOG1\_pbs2PP\_phosphorylation\_176\_mono}*\text{feedback\_pbs2\_mono}$ |
| Hog1P176c + Pbs2PP_phosphorylated -> Hog1P176c_Pbs2PP_phosphorylated | $\left[\text{Hog1P176c}\right]*\left[\text{Pbs2PP\_phosphorylated}\right]*\text{HOG1\_pbs2pp\_formation\_P}*\text{feedback\_pbs2\_formation\_p}$ |
| Hog1P174c + Pbs2PP_phosphorylated -> Hog1P174c_Pbs2PP_phosphorylated | $\left[\text{Hog1P174c}\right]*\left[\text{Pbs2PP\_phosphorylated}\right]*\text{HOG1\_pbs2pp\_formation\_P}*\text{feedback\_pbs2\_formation\_p}$ |
| Hog1P174c_Pbs2PP_phosphorylated -> Hog1P174c + Pbs2PP_phosphorylated | $\left[\text{Hog1P174c\_Pbs2PP\_phosphorylated}\right]*\text{HOG1\_pbs2PP\_break\_up\_P}*\text{feedback\_pbs2\_break\_p}$ |
| Hog1P176c_Pbs2PP_phosphorylated -> Hog1P176c + Pbs2PP_phosphorylated | $\left[\text{Hog1P176c\_Pbs2PP\_phosphorylated}\right]*\text{HOG1\_pbs2PP\_break\_up\_P}*\text{feedback\_pbs2\_break\_p}$ |
| Hog1P174c_Pbs2PP_phosphorylated -> Hog1PPc_Pbs2PP_phosphorylated | $\left[\text{Hog1P174c\_Pbs2PP\_phosphorylated}\right]*\text{HOG1\_pbs2PP\_phosphorylation\_176\_duo}*\text{feedback\_pbs2\_duo}$ |
| Hog1P176c_Pbs2PP_phosphorylated -> Hog1PPc_Pbs2PP_phosphorylated | $\left[\text{Hog1P176c\_Pbs2PP\_phosphorylated}\right]*\text{HOG1\_pbs2PP\_phosphorylation\_174\_duo}*\text{feedback\_pbs2\_duo}$ |
| Hog1PPc + Pbs2PP_phosphorylated -> Hog1PPc_Pbs2PP_phosphorylated | $\left[\text{Hog1PPc}\right]*\left[\text{Pbs2PP\_phosphorylated}\right]*\text{HOG1\_pbs2pp\_formation\_PP}*\text{feedback\_pbs2\_formation\_pp}$ |
| Hog1PPc_Pbs2PP_phosphorylated -> Hog1PPc + Pbs2PP_phosphorylated | $\left[\text{Hog1PPc\_Pbs2PP\_phosphorylated}\right]*\text{HOG1\_pbs2PP\_break\_up\_PP}*\text{feedback\_pbs2\_break\_up\_pp}$ |
| **Formation of Hog1 – Pbs2 complexes that act as targets for the Ptc1 phosphatase** | |
| Hog1c + Pbs2 -> Hog1c_Pbs2 | $\left[\text{Hog1c}\right]*\left[\text{Pbs2}\right]*\text{HOG1\_pbs2pp\_formation}$ |
| Hog1c_Pbs2 -> Hog1c + Pbs2 | $\left[\text{Hog1c\_Pbs2}\right]*\text{HOG1\_pbs2PP\_break\_up}$ |
| Hog1P176c + Pbs2 -> Hog1P176c_Pbs2 | $\left[\text{Hog1P176c}\right]*\left[\text{Pbs2}\right]*\text{HOG1\_pbs2pp\_formation\_P}$ |
| Hog1P174c + Pbs2 -> Hog1P174c_Pbs2 | $\left[\text{Hog1P174c}\right]*\left[\text{Pbs2}\right]*\text{HOG1\_pbs2pp\_formation\_P}$ |
| Hog1P174c_Pbs2 -> Hog1P174c + Pbs2 | $\left[\text{Hog1P174c\_Pbs2}\right]*\text{HOG1\_pbs2PP\_break\_up\_P}$ |
| Hog1P176c_Pbs2 -> Hog1P176c + Pbs2 | $\left[\text{Hog1P176c\_Pbs2}\right]*\text{HOG1\_pbs2PP\_break\_up\_P}$ |
| Hog1PPc + Pbs2 -> Hog1PPc_Pbs2 | $\left[\text{Hog1PPc}\right]*\left[\text{Pbs2}\right]*\text{HOG1\_pbs2pp\_formation\_PP}$ |
| Hog1PPc_Pbs2 -> Hog1PPc + Pbs2 | $\left[\text{Hog1PPc\_Pbs2}\right]*\text{HOG1\_pbs2PP\_break\_up\_PP}$ |
| **Formation of Hog1 – Pbs2 with feedback complexes that act as targets for the Ptc1 phosphatase** | |
| Hog1c + Pbs2_phosphorylated -> Hog1c_Pbs2_phosphorylated | $\left[\text{Hog1c}\right]*\left[\text{Pbs2\_phosphorylated}\right]*\text{HOG1\_pbs2pp\_formation}*\text{feedback\_pbs2\_formation}$ |
| Hog1c_Pbs2_phosphorylated -> Hog1c + Pbs2_phosphorylated | $\left[\text{Hog1c\_Pbs2\_phosphorylated}\right]*\text{HOG1\_pbs2PP\_break\_up}*\text{feedback\_pbs2\_break\_up}$ |
| Hog1P176c + Pbs2_phosphorylated -> Hog1P176c_Pbs2_phosphorylated | $\left[\text{Hog1P176c}\right]*\left[\text{Pbs2\_phosphorylated}\right]*\text{HOG1\_pbs2pp\_formation\_P}*\text{feedback\_pbs2\_formation\_p}$ |
| Hog1P174c + Pbs2_phosphorylated -> Hog1P174c_Pbs2_phosphorylated | $\left[\text{Hog1P174c}\right]*\left[\text{Pbs2\_phosphorylated}\right]*\text{HOG1\_pbs2pp\_formation\_P}*\text{feedback\_pbs2\_formation\_p}$ |
| Hog1P174c_Pbs2_phosphorylated -> Hog1P174c + Pbs2_phosphorylated | $\left[\text{Hog1P174c\_Pbs2\_phosphorylated}\right]*\text{HOG1\_pbs2PP\_break\_up\_P}*\text{feedback\_pbs2\_break\_p}$ |
| Hog1P176c_Pbs2_phosphorylated -> Hog1P176c + Pbs2_phosphorylated | $\left[\text{Hog1P176c\_Pbs2\_phosphorylated}\right]*\text{HOG1\_pbs2PP\_break\_up\_P}*\text{feedback\_pbs2\_break\_p}$ |
| Hog1PPc + Pbs2_phosphorylated -> Hog1PPc_Pbs2_phosphorylated | $\left[\text{Hog1PPc}\right]*\left[\text{Pbs2\_phosphorylated}\right]*\text{HOG1\_pbs2pp\_formation\_PP}*\text{feedback\_pbs2\_formation\_pp}$ |
| Hog1PPc_Pbs2_phosphorylated -> Hog1PPc + Pbs2_phosphorylated | $\left[\text{Hog1PPc\_Pbs2\_phosphorylated}\right]*\text{HOG1\_pbs2PP\_break\_up\_PP}*\text{feedback\_pbs2\_break\_up\_pp}$ |
|  |  |

| Parameter | Parameter value^a^ | Unit | Additional information |
| --- | --- | --- | --- |
| HOG1_pbs2pp_formation | 153.85 | mM^-1^s^-1^ | Rate of association between Hog1 and Pbs2PP |
| HOG1_pbs2pp_break_up | 921.51 | s^-1^ | Rate of disassociation between Hog1 and Pbs2PP |
| HOG1_pbs2pp_formation_P | 60.39 | mM^-1^s^-1^ | Rate of association between monophosphorylated Hog1 and Pbs2PP |
| HOG1_pbs2pp_break_up_P | 828.51 | s^-1^ | Rate of disassociation between monophosphorylated Hog1 and Pbs2PP |
| HOG1_pbs2pp_formation_PP | 180.55 | mM^-1^s^-1^ | Rate of association between double phosphorylated Hog1 and Pbs2PP |
| HOG1_pbs2pp_break_up_PP | 56.34 | s^-1^ | Rate of disassociation between double phosphorylated Hog1 and Pbs2PP |
|  |  |  |  |
| HOG1_pbs2pp_phosphorylation_174_duo | 4.23 | s^-1^ | Phosphorylation of Thr174 on Hog1P176 |
| HOG1_pbs2pp_phosphorylation_174_mono | 71.17 | s^-1^ | Phosphorylation of Thr174 on non-phosphorylated Hog1 |
| HOG1_pbs2pp_phosphorylation_176_duo | 1.94 | s^-1^ | Phosphorylation of Tyr176 on Hog1P174 |
| HOG1_pbs2pp_phosphorylation_176_mono | 290.54 | s^-1^ | Phosphorylation of Tyr176 on non-phosphorylated Hog1 |
|  |  |  |  |
| feedback_pbs2_formation | 0.89 | dimensionless | Change in the association rate between Hog1 and Pbs2 |
| feedback_pbs2_break_up | 6.18 | dimensionless | Change in the disassociation rate between Hog1 and Pbs2 |
| feedback_pbs2_formation_p | 0.18 | dimensionless | Change in the association rate between Hog1P and Pbs2 |
| feedback_pbs2_break_p | 0.16 | dimensionless | Change in the disassociation rate between Hog1P and Pbs2 |
| feedback_pbs2_formation_pp | 1.67 | dimensionless | Change in the association rate between Hog1PP and Pbs2 |
| feedback_pbs2_break_up_pp | 5.52 | dimensionless | Change in the disassociation rate between Hog1 and Pbs2 |
| feedback_pbs2_mono | 2.40 | dimensionless | Change in the phosphorylation rate of the first phosphorylation of Hog1 |
| feedback_pbs2_duo | 66.70 | dimensionless | Change in the phosphorylation rate of the second phosphorylation of Hog1 |

| **Hog1 dephosphorylation** | |
| --- | --- |
| Hog1PPc + additional_phosphatase_cyto -> Hog1P174c + additional_phosphatase_cyto | $\left[\text{Hog1PPc}\right]*\left[\text{additional\_phosphatase\_cyto}\right]*\text{DEPHOSPHORYLATION\_hog1ppc\_to\_Hog1P174c\_by\_additional\_phosphatase}$ |
| Hog1PPn + additional_phosphatase_nucleus -> Hog1P174n + additional_phosphatase_nucleus | $\left[\text{Hog1PPn}\right]*\left[\text{additional\_phosphatase\_nucleus}\right]*\text{DEPHOSPHORYLATION\_Hog1PPn\_to\_hog1p174n\_by\_additional\_phosphatase}$ |
| Hog1P176c + additional_phosphatase_cyto -> Hog1c + additional_phosphatase_cyto | $\left[\text{Hog1P176c}\right]*\left[\text{additional\_phosphatase\_cyto}\right]*\text{DEPHOSPHORYLATION\_Hog1P176c\_to\_hog1c\_by\_additional\_phosphatase}$ |
| Hog1P176n + additional_phosphatase_nucleus -> Hog1n + additional_phosphatase_nucleus | $\left[\text{Hog1P176n}\right]*\left[\text{additional\_phosphatase\_nucleus}\right]*\text{DEPHOSPHORYLATION\_Hog1P176n\_to\_hog1c\_by\_additional\_phosphatase}$ |
|  |  |
| Hog1PPc + Ptp3 -> Hog1P174c + Ptp3 | $\left[\text{Hog1PPc}\right]*\left[\text{Ptp3}\right]*\text{DEPHOSPHORYLATION\_hog1ppc\_to\_Hog1P174c\_by\_Ptp3}*\text{Ptp3\_factor}$ |
| Hog1P176c + Ptp3 -> Hog1c + Ptp3 | $\left[\text{Hog1P176c}\right]*\left[\text{Ptp3}\right]*\text{DEPHOSPHORYLATION\_hog1Pq76c\_to\_hog1c\_by\_ptp3}*\text{Ptp3\_factor}$ |
|  |  |
| Hog1PPc_Pbs2PP + Ptc1 -> Hog1P176c_Pbs2PP + Ptc1 | $\left[\text{Hog1PPc\_Pbs2PP}\right]*\left[\text{Ptc1}\right]*\text{DEPHOSPHORYLATION\_Hog1PPc\_to\_hog1P176\_by\_ptc1}$ |
| Hog1PPc_Pbs2 + Ptc1 -> Hog1P176c_Pbs2 + Ptc1 | $\left[\text{Hog1PPc\_Pbs2}\right]*\left[\text{Ptc1}\right]*\text{DEPHOSPHORYLATION\_Hog1PPc\_to\_hog1P176\_by\_ptc1}$ |
| Hog1PPc_Pbs2PP_phosphorylated + Ptc1 -> Hog1P176c_Pbs2PP_phosphorylated + Ptc1 | $\left[\text{Hog1PPc\_Pbs2PP\_phosphorylated}\right]*\left[\text{Ptc1}\right]*\text{DEPHOSPHORYLATION\_Hog1PPc\_to\_hog1P176\_by\_ptc1}$ |
| Hog1PPc_Pbs2_phosphorylated + Ptc1 -> Hog1P176c_Pbs2_phosphorylated + Ptc1 | $\left[\text{Hog1PPc\_Pbs2\_phosphorylated}\right]*\left[\text{Ptc1}\right]*\text{DEPHOSPHORYLATION\_Hog1PPc\_to\_hog1P176\_by\_ptc1}$ |
| Hog1P174c_Pbs2PP + Ptc1 -> Hog1c_Pbs2PP + Ptc1 | $\left[\text{Hog1P174c\_Pbs2PP}\right]*\left[\text{Ptc1}\right]*\text{DEPHOSPHORYLATION\_Hog1P174c\_to\_hog1c\_by\_ptc1}$ |
| Hog1P174c_Pbs2 + Ptc1 -> Hog1c_Pbs2 + Ptc1 | $\left[\text{Hog1P174c\_Pbs2}\right]*\left[\text{Ptc1}\right]*\text{DEPHOSPHORYLATION\_Hog1P174c\_to\_hog1c\_by\_ptc1}$ |
| Hog1P174c_Pbs2PP_phosphorylated + Ptc1 -> Hog1c_Pbs2PP_phosphorylated + Ptc1 | $\left[\text{Hog1P174c\_Pbs2PP\_phosphorylated}\right]*\left[\text{Ptc1}\right]*\text{DEPHOSPHORYLATION\_Hog1P174c\_to\_hog1c\_by\_ptc1}$ |
| Hog1P174c_Pbs2_phosphorylated + Ptc1 -> Hog1c_Pbs2_phosphorylated + Ptc1 | $\left[\text{Hog1P174c\_Pbs2\_phosphorylated}\right]*\left[\text{Ptc1}\right]*\text{DEPHOSPHORYLATION\_Hog1P174c\_to\_hog1c\_by\_ptc1}$ |
|  |  |
| Hog1PPn + Ptp2 -> Hog1P174n + Ptp2 | $\left[\text{Hog1PPn}\right]*\left[\text{Ptp2}\right]*\text{DEPHOSPHORYLATION\_Hog1PPn\_to\_hog1p174n\_by\_ptp2}*\text{Ptp2\_factor}$ |
| Hog1PPn + Ptc -> Hog1P176n + Ptc | $\left[\text{Hog1PPn}\right]*\left[\text{Ptc}\right]*\text{DEPHOSPHORYLATION\_Hog1PPn\_to\_hog1P174n\_by\_ptc}$ |
| Hog1P174n + Ptc -> Hog1n + Ptc | $\left[\text{Hog1P174n}\right]*\left[\text{Ptc}\right]*\text{DEPHOSPHORYLATION\_Hog1P174n\_to\_hog1n\_by\_ptc}$ |
| Hog1P176n + Ptp2 -> Hog1n + Ptp2 | $\left[\text{Hog1P174n}\right]*\left[\text{Ptp2}\right]*\text{DEPHOSPHORYLATION\_Hog1P176n\_to\_hog1n\_by\_ptp2}$ |

| Parameter | Parameter value^a^ | Unit | Additional information |
| --- | --- | --- | --- |
| DEPHOSPHORYLATION_Hog1PPn_to_hog1p174n_by_additional_phosphatase | 0.0019 | mM^-1^s^-1^ | Dephosphorylation by unspecific phosphatases |
| DEPHOSPHORYLATION_hog1ppc_to_Hog1P174c_by_additional_phosphatase | 2e-19 | mM^-1^s^-1^ | Dephosphorylation by unspecific phosphatases |
| DEPHOSPHORYLATION_Hog1P176c_to_hog1c_by_additional_phosphatase | 3.68e-8 | mM^-1^s^-1^ | Dephosphorylation by unspecific phosphatases |
| DEPHOSPHORYLATION_Hog1P176n_to_hog1n_by_additional_phosphatase | 0.0133 | mM^-1^s^-1^ | Dephosphorylation by unspecific phosphatases |
|  |  |  |  |
| DEPHOSPHORYLATION_hog1ppc_to_Hog1P174c_by_Ptp3 | 7.3621e-17 | mM^-1^s^-1^ | Dephosphorylation of Tyrosine in the cytosol |
| DEPHOSPHORYLATION_Hog1P176c_to_hog1c_by_ptp3 | 0.0019 | mM^-1^s^-1^ | Dephosphorylation of Tyrosine in the cytosol |
| DEPHOSPHORYLATION_Hog1PPn_to_hog1p174n_by_ptp2 | 2.18e-5 | mM^-1^s^-1^ | Dephosphorylation of Tyrosine in the nucleus |
| DEPHOSPHORYLATION_Hog1P176n_to_hog1n_by_ptp2 | 2.0735 | mM^-1^s^-1^ | Dephosphorylation of Tyrosine in the cytosol |
| DEPHOSPHORYLATION_Hog1P174c_to_hog1c_by_ptc1 | 2.3318 | s^-1^ | Dephosphorylation by Ptc1 when bound to Pbs2 |
| DEPHOSPHORYLATION_Hog1PPc_to_hog1P176_by_ptc1 | 5.46e-4 | s^-1^ | Dephosphorylation by Ptc1 when bound to Pbs2 |
| DEPHOSPHORYLATION_Hog1P174n_to_hog1n_by_ptc | 0.5077 | mM^-1^s^-1^ | Dephosphorylation by Ptc2/3 in Nucleus |
| DEPHOSPHORYLATION_Hog1PPn_to_hog1P176n_by_ptc | 0.0563 | mM^-1^s^-1^ | Dephosphorylation by Ptc2/3 in Nucleus |

| Species | Initial value (unit) | Additional information |
| --- | --- | --- |
| Hog1c_a_ | 0.3109 (mM) | Cytosolic Hog1 |
| Hog1n | 0.5278 (mM) | Nuclear Hog1 |
| Hog1PPc | 0.0105 (mM) | Double phosphorylated, cytosolic Hog1 |
| Hog1PPn | 0 (mM) | Double phosphorylated, nuclear Hog1 |
| Hog1P176c | 0.0303 (mM) | Cytosolic Hog1 phosphorylated at Tyr-176 |
| Hog1P176n | 0 (mM) | Nuclear Hog1 phosphorylated at Tyr-176 |
| Hog1P174c | 0.0026 (mM) | Cytosolic Hog1 phosphorylated at Thr-174 |
| Hog1P174n | 0 (mM) | Nuclear Hog1 phosphorylated at Thr-174 |
| Pbs2PP | 0 (mM) | activated, double phosphorylated Pbs2 |
| Hog1c_Pbs2PP | 0 (mM) | Hog1 bound to activated, double phosphorylated Pbs2 |
| Hog1P174c_Pbs2PP | 0 (mM) | Hog1 phosphorylated at Thr-174 bound to activated, double phosphorylated Pbs2 |
| Hog1P176c_Pbs2PP | 0 (mM) | Hog1 phosphorylated at Tyr-176 bound to activated, double phosphorylated Pbs2 |
| Hog1PPc_Pbs2PP | 0 (mM) | Double phosphorylated Hog1 bound to activated, double phosphorylated Pbs2 |
| Pbs2PP_phosphorylated | 0 (mM) | activated, double phosphorylated Pbs2 with additional feedback phosphorylation |
| Hog1c_Pbs2PP_phosphorylated | 0 (mM) | Hog1 bound to activated, double phosphorylated Pbs2 with additional feedback phosphorylation |
| Hog1P174c_Pbs2PP_phosphorylated | 0 (mM) | Hog1 phosphorylated at Thr-174 bound to activated, double phosphorylated Pbs2 with additional feedback phosphorylation |
| Hog1P176c_Pbs2PP_phosphorylated | 0 (mM) | Hog1 phosphorylated at Tyr-176 bound to activated, double phosphorylated Pbs2 with additional feedback phosphorylation |
| Hog1PPc_Pbs2PP_phosphorylated | 0 (mM) | Double phosphorylated Hog1 bound to activated, double phosphorylated Pbs2 with additional feedback phosphorylation |
| Pbs2_b_ | 0.1138 (mM) | MAPKK of Hog1 |
| Hog1P176c_Pbs2 | 0 (mM) | Hog1 phosphorylated at Tyr-176 bound to Pbs2 |
| Hog1P174c_Pbs2 | 0 (mM) | Hog1 phosphorylated at Thr-174 bound to Pbs2 |
| Hog1PPc_Pbs2 | 0 (mM) | Double phosphorylated Hog1 bound to Pbs2 |
| Pbs2_phosphorylated | 0.01 (mM) | Pbs2 with additional feedback phosphorylation |
| Pbs2PP_phosphorylated | 1.26e-6 (mM) | activated, double phosphorylated Pbs2 with additional feedback phosphorylation |
| Hog1P176c_Pbs2_phosphorylated | 0 (mM) | Hog1 phosphorylated at Tyr-176 bound to Pbs2 with additional feedback phosphorylation |
| Hog1P174c_Pbs2_phosphorylated | 0 (mM) | Hog1 phosphorylated at Thr-174 bound to Pbs2 with additional feedback phosphorylation |
| Hog1PPc_Pbs2_phosphorylated | 0 (mM) | Double phosphorylated Hog1 bound to Pbs2 with additional feedback phosphorylation |
| additional_phosphatase_cyto | 1 (mM) | Arbitrary approximation of all unspecific phosphatases in the cytosol |
| additional_phosphatase_nucleus | 1 (mM) | Arbitrary approximation of all unspecific phosphatases in the nucleus |
| Ptp3_c_ | 0.48 (mM) | Cytosolic Ptp3 |
| Ptc1_d_ | 0.0943 (mM) | Cytosolic Ptc1 |
| Ptp2_e_ | 0.089 (mM) | Nuclear Ptp2 |
| Ptc_f_ | 1.3576 (mM) | Nuclear proportion of Ptc2/3 when molecules are evenly distributed throughout cell |

_a_ Total amount of Hog1 in the cell: 6780 molecules^23^

_b_ Total amount of Pbs2 in the cell: 2160 molecules^23^

_c_ Total amount of Ptp3 in the cell: 768 molecules^23^

_d_ Total amount of Ptc1 in the cell: 1520 molecules^23^

_e_ Total amount of Ptp2 in the cell: 149 molecules^23^

_f_ Total amount of Ptc2/3 in the nucleus: 7% of 12600 molecules of Ptc2^23^ and 19700 molecules of Ptc3^23^

**Reactions used for the distributive mechanism of Hog1 phosphorylation**

| **Hog1 phosphorylation distributive mechanism** | |
| --- | --- |
| Hog1c + Pbs2PP -> Hog1c_Pbs2PP | $\left[\text{Hog1c}\right]*\left[\text{Pbs2PP}\right]*\text{HOG1\_pbs2pp\_formation}$ |
| Hog1c_Pbs2PP -> Hog1c + Pbs2PP | $\left[\text{Hog1c\_Pbs2PP}\right]*\text{HOG1\_pbs2PP\_break\_up}$ |
| Hog1c_Pbs2PP -> Hog1P174c + Pbs2PP | $\left[\text{Hog1c\_Pbs2PP}\right]*\text{HOG1\_pbs2PP\_phosphorylation\_174\_mono}$ |
| Hog1c_Pbs2PP -> Hog1P176c + Pbs2PP | $\left[\text{Hog1c\_Pbs2PP}\right]*\text{HOG1\_pbs2PP\_phosphorylation\_176\_mono}$ |
| Hog1P176c + Pbs2PP -> Hog1P176c_Pbs2PP | $\left[\text{Hog1P176c}\right]*\left[\text{Pbs2PP}\right]*\text{HOG1\_pbs2pp\_formation\_P}$ |
| Hog1P174c + Pbs2PP -> Hog1P174c_Pbs2PP | $\left[\text{Hog1P174c}\right]*\left[\text{Pbs2PP}\right]*\text{HOG1\_pbs2pp\_formation\_P}$ |
| Hog1P174c_Pbs2PP -> Hog1P174c + Pbs2PP | $\left[\text{Hog1P174c\_Pbs2PP}\right]*\text{HOG1\_pbs2PP\_break\_up\_P}$ |
| Hog1P176c_Pbs2PP -> Hog1P176c + Pbs2PP | $\left[\text{Hog1P176c\_Pbs2PP}\right]*\text{HOG1\_pbs2PP\_break\_up\_P}$ |
| Hog1P174c_Pbs2PP -> Hog1PPc_Pbs2PP | $\left[\text{Hog1P174c\_Pbs2PP}\right]*\text{HOG1\_pbs2PP\_phosphorylation\_176\_duo}$ |
| Hog1P176c_Pbs2PP -> Hog1PPc_Pbs2PP | $\left[\text{Hog1P176c\_Pbs2PP}\right]*\text{HOG1\_pbs2PP\_phosphorylation\_174\_duo}$ |
| Hog1PPc + Pbs2PP -> Hog1PPc_Pbs2PP | $\left[\text{Hog1PPc}\right]*\left[\text{Pbs2PP}\right]*\text{HOG1\_pbs2pp\_formation\_PP}$ |
| Hog1PPc_Pbs2PP -> Hog1PPc + Pbs2PP | $\left[\text{Hog1PPc\_Pbs2PP}\right]*\text{HOG1\_pbs2PP\_break\_up\_PP}$ |
| Hog1c + Pbs2PP_phosphorylated -> Hog1c_Pbs2PP_phosphorylated | $\left[\text{Hog1c}\right]*\left[\text{Pbs2PP\_phosphorylated}\right]*\text{HOG1\_pbs2pp\_formation}*\text{feedback\_pbs2\_formation}$ |
| Hog1c_Pbs2PP_phosphorylated -> Hog1c + Pbs2PP_phosphorylated | $\left[\text{Hog1c\_Pbs2PP\_phosphorylated}\right]*\text{HOG1\_pbs2PP\_break\_up}*\text{feedback\_pbs2\_break\_up}$ |
| Hog1c_Pbs2PP_phosphorylated -> Hog1P174c + Pbs2PP_phosphorylated | $\left[\text{Hog1c\_Pbs2PP\_phosphorylated}\right]*\text{HOG1\_pbs2PP\_phosphorylation\_174\_mono}*\text{feedback\_pbs2\_mono}$ |
| Hog1c_Pbs2PP_phosphorylated -> Hog1P176c + Pbs2PP_phosphorylated | $\left[\text{Hog1c\_Pbs2PP\_phosphorylated}\right]*\text{HOG1\_pbs2PP\_phosphorylation\_176\_mono}*\text{feedback\_pbs2\_mono}$ |
| Hog1P176c + Pbs2PP_phosphorylated -> Hog1P176c_Pbs2PP_phosphorylated | $\left[\text{Hog1P176c}\right]*\left[\text{Pbs2PP\_phosphorylated}\right]*\text{HOG1\_pbs2pp\_formation\_P}*\text{feedback\_pbs2\_formation\_p}$ |
| Hog1P174c + Pbs2PP_phosphorylated -> Hog1P174c_Pbs2PP_phosphorylated | $\left[\text{Hog1P174c}\right]*\left[\text{Pbs2PP\_phosphorylated}\right]*\text{HOG1\_pbs2pp\_formation\_P}*\text{feedback\_pbs2\_formation\_p}$ |
| Hog1P174c_Pbs2PP_phosphorylated -> Hog1P174c + Pbs2PP_phosphorylated | $\left[\text{Hog1P174c\_Pbs2PP\_phosphorylated}\right]*\text{HOG1\_pbs2PP\_break\_up\_P}*\text{feedback\_pbs2\_break\_p}$ |
| Hog1P176c_Pbs2PP_phosphorylated -> Hog1P176c + Pbs2PP_phosphorylated | $\left[\text{Hog1P176c\_Pbs2PP\_phosphorylated}\right]*\text{HOG1\_pbs2PP\_break\_up\_P}*\text{feedback\_pbs2\_break\_p}$ |
| Hog1P174c_Pbs2PP_phosphorylated -> Hog1PPc_Pbs2PP_phosphorylated | $\left[\text{Hog1P174c\_Pbs2PP\_phosphorylated}\right]*\text{HOG1\_pbs2PP\_phosphorylation\_176\_duo}*\text{feedback\_pbs2\_duo}$ |
| Hog1P176c_Pbs2PP_phosphorylated -> Hog1PPc_Pbs2PP_phosphorylated | $\left[\text{Hog1P176c\_Pbs2PP\_phosphorylated}\right]*\text{HOG1\_pbs2PP\_phosphorylation\_174\_duo}*\text{feedback\_pbs2\_duo}$ |
| Hog1PPc + Pbs2PP_phosphorylated -> Hog1PPc_Pbs2PP_phosphorylated | $\left[\text{Hog1PPc}\right]*\left[\text{Pbs2PP\_phosphorylated}\right]*\text{HOG1\_pbs2pp\_formation\_PP}*\text{feedback\_pbs2\_formation\_pp}$ |
| Hog1PPc_Pbs2PP_phosphorylated -> Hog1PPc + Pbs2PP_phosphorylated | $\left[\text{Hog1PPc\_Pbs2PP\_phosphorylated}\right]*\text{HOG1\_pbs2PP\_break\_up\_PP}*\text{feedback\_pbs2\_break\_up\_pp}$ |

**Reactions used for the processive mechanism of Hog1 phosphorylation (reactions highlighted in red have been removed)**

| **Hog1 phosphorylation processive mechanism** | |
| --- | --- |
| Hog1c + Pbs2PP -> Hog1c_Pbs2PP | $\left[\text{Hog1c}\right]*\left[\text{Pbs2PP}\right]*\text{HOG1\_pbs2pp\_formation}$ |
| Hog1c_Pbs2PP -> Hog1c + Pbs2PP | $\left[\text{Hog1c\_Pbs2PP}\right]*\text{HOG1\_pbs2PP\_break\_up}$ |
| Hog1c_Pbs2PP -> Hog1P174c_Pbs2PP | $\left[\text{Hog1c\_Pbs2PP}\right]*\text{HOG1\_pbs2PP\_phosphorylation\_174\_mono}$ |
| Hog1c_Pbs2PP -> Hog1P176c_Pbs2PP | $\left[\text{Hog1c\_Pbs2PP}\right]*\text{HOG1\_pbs2PP\_phosphorylation\_176\_mono}$ |
| Hog1P176c + Pbs2PP -> Hog1P176c_Pbs2PP | $\left[\text{Hog1P176c}\right]*\left[\text{Pbs2PP}\right]*\text{HOG1\_pbs2pp\_formation\_P}$ |
| Hog1P174c + Pbs2PP -> Hog1P174c_Pbs2PP | $\left[\text{Hog1P174c}\right]*\left[\text{Pbs2PP}\right]*\text{HOG1\_pbs2pp\_formation\_P}$ |
| Hog1P174c_Pbs2PP -> Hog1P174c + Pbs2PP | $\left[\text{Hog1P174c\_Pbs2PP}\right]*\text{HOG1\_pbs2PP\_break\_up\_P}$ |
| Hog1P176c_Pbs2PP -> Hog1P176c + Pbs2PP | $\left[\text{Hog1P176c\_Pbs2PP}\right]*\text{HOG1\_pbs2PP\_break\_up\_P}$ |
| Hog1P174c_Pbs2PP -> Hog1PPc_Pbs2PP | $\left[\text{Hog1P174c\_Pbs2PP}\right]*\text{HOG1\_pbs2PP\_phosphorylation\_176\_duo}$ |
| Hog1P176c_Pbs2PP -> Hog1PPc_Pbs2PP | $\left[\text{Hog1P176c\_Pbs2PP}\right]*\text{HOG1\_pbs2PP\_phosphorylation\_174\_duo}$ |
| Hog1PPc + Pbs2PP -> Hog1PPc_Pbs2PP | $\left[\text{Hog1PPc}\right]*\left[\text{Pbs2PP}\right]*\text{HOG1\_pbs2pp\_formation\_PP}$ |
| Hog1PPc_Pbs2PP -> Hog1PPc + Pbs2PP | $\left[\text{Hog1PPc\_Pbs2PP}\right]*\text{HOG1\_pbs2PP\_break\_up\_PP}$ |
| Hog1c + Pbs2PP_phosphorylated -> Hog1c_Pbs2PP_phosphorylated | $\left[\text{Hog1c}\right]*\left[\text{Pbs2PP\_phosphorylated}\right]*\text{HOG1\_pbs2pp\_formation}*\text{feedback\_pbs2\_formation}$ |
| Hog1c_Pbs2PP_phosphorylated -> Hog1c + Pbs2PP_phosphorylated | $\left[\text{Hog1c\_Pbs2PP\_phosphorylated}\right]*\text{HOG1\_pbs2PP\_break\_up}*\text{feedback\_pbs2\_break\_up}$ |
| Hog1c_Pbs2PP_phosphorylated -> Hog1P174c_Pbs2PP_phosphorylated | $\left[\text{Hog1c\_Pbs2PP\_phosphorylated}\right]*\text{HOG1\_pbs2PP\_phosphorylation\_174\_mono}*\text{feedback\_pbs2\_mono}$ |
| Hog1c_Pbs2PP_phosphorylated -> Hog1P176c_Pbs2PP_phosphorylated | $\left[\text{Hog1c\_Pbs2PP\_phosphorylated}\right]*\text{HOG1\_pbs2PP\_phosphorylation\_176\_mono}*\text{feedback\_pbs2\_mono}$ |
| Hog1P176c + Pbs2PP_phosphorylated -> Hog1P176c_Pbs2PP_phosphorylated | $\left[\text{Hog1P176c}\right]*\left[\text{Pbs2PP\_phosphorylated}\right]*\text{HOG1\_pbs2pp\_formation\_P}*\text{feedback\_pbs2\_formation\_p}$ |
| Hog1P174c + Pbs2PP_phosphorylated -> Hog1P174c_Pbs2PP_phosphorylated | $\left[\text{Hog1P174c}\right]*\left[\text{Pbs2PP\_phosphorylated}\right]*\text{HOG1\_pbs2pp\_formation\_P}*\text{feedback\_pbs2\_formation\_p}$ |
| Hog1P174c_Pbs2PP_phosphorylated -> Hog1P174c + Pbs2PP_phosphorylated | $\left[\text{Hog1P174c\_Pbs2PP\_phosphorylated}\right]*\text{HOG1\_pbs2PP\_break\_up\_P}*\text{feedback\_pbs2\_break\_p}$ |
| Hog1P176c_Pbs2PP_phosphorylated -> Hog1P176c + Pbs2PP_phosphorylated | $\left[\text{Hog1P176c\_Pbs2PP\_phosphorylated}\right]*\text{HOG1\_pbs2PP\_break\_up\_P}*\text{feedback\_pbs2\_break\_p}$ |
| Hog1P174c_Pbs2PP_phosphorylated -> Hog1PPc_Pbs2PP_phosphorylated | $\left[\text{Hog1P174c\_Pbs2PP\_phosphorylated}\right]*\text{HOG1\_pbs2PP\_phosphorylation\_176\_duo}*\text{feedback\_pbs2\_duo}$ |
| Hog1P176c_Pbs2PP_phosphorylated -> Hog1PPc_Pbs2PP_phosphorylated | $\left[\text{Hog1P176c\_Pbs2PP\_phosphorylated}\right]*\text{HOG1\_pbs2PP\_phosphorylation\_174\_duo}*\text{feedback\_pbs2\_duo}$ |
| Hog1PPc + Pbs2PP_phosphorylated -> Hog1PPc_Pbs2PP_phosphorylated | $\left[\text{Hog1PPc}\right]*\left[\text{Pbs2PP\_phosphorylated}\right]*\text{HOG1\_pbs2pp\_formation\_PP}*\text{feedback\_pbs2\_formation\_pp}$ |
| Hog1PPc_Pbs2PP_phosphorylated -> Hog1PPc + Pbs2PP_phosphorylated | $\left[\text{Hog1PPc\_Pbs2PP\_phosphorylated}\right]*\text{HOG1\_pbs2PP\_break\_up\_PP}*\text{feedback\_pbs2\_break\_up\_pp}$ |

**Supp Table S10: Hog1 activation**

Description of the reactions, reaction rates, parameter values and species of the model simulating the Hog1 activation including Hog1 phosphorylation by Pbs2, Hog1 dephosphorylation, Hog1 shuttling into the nucleus and the differences between distributive, processive and mixed activation of Hog1.

**References**

1. Bar-Even, A. *et al.* The moderately efficient enzyme: evolutionary and physicochemical trends shaping enzyme parameters. *Biochemistry* **50**, 4402–4410 (2011).

2. Klipp, E., Nordlander, B., Krüger, R., Gennemark, P. & Hohmann, S. Integrative model of the response of yeast to osmotic shock. *Nat. Biotechnol.* **23**, 975–982 (2005).

3. Zi, Z., Liebermeister, W. & Klipp, E. A quantitative study of the Hog1 MAPK response to fluctuating osmotic stress in Saccharomyces cerevisiae. *PloS One* **5**, e9522 (2010).

4. Petelenz-Kurdziel, E. *et al.* Quantitative analysis of glycerol accumulation, glycolysis and growth under hyper osmotic stress. *PLoS Comput. Biol.* **9**, e1003084 (2013).

5. de Nadal, E. & Posas, F. The HOG pathway and the regulation of osmoadaptive responses in yeast. *FEMS Yeast Res.* **22**, foac013 (2022).

6. Tamás, M. J. *et al.* Fps1p controls the accumulation and release of the compatible solute glycerol in yeast osmoregulation. *Mol. Microbiol.* **31**, 1087–1104 (1999).

7. Lee, J. *et al.* MAPK Hog1 closes the S. cerevisiae glycerol channel Fps1 by phosphorylating and displacing its positive regulators. *Genes Dev.* **27**, 2590–2601 (2013).

8. Muzzey, D., Gómez-Uribe, C. A., Mettetal, J. T. & van Oudenaarden, A. A systems-level analysis of perfect adaptation in yeast osmoregulation. *Cell* **138**, 160–171 (2009).

9. Westfall, P. J., Patterson, J. C., Chen, R. E. & Thorner, J. Stress resistance and signal fidelity independent of nuclear MAPK function. *Proc. Natl. Acad. Sci.* **105**, 12212–12217 (2008).

10. Huang, C.-Y. & Ferrell Jr, J. E. Ultrasensitivity in the mitogen-activated protein kinase cascade. *Proc. Natl. Acad. Sci.* **93**, 10078–10083 (1996).

11. Macia, J. *et al.* Dynamic signaling in the Hog1 MAPK pathway relies on high basal signal transduction. *Sci. Signal.* **2**, ra13–ra13 (2009).

12. English, J. G. *et al.* MAPK feedback encodes a switch and timer for tunable stress adaptation in yeast. *Sci. Signal.* **8**, ra5–ra5 (2015).

13. Granados, A. A. *et al.* Distributing tasks via multiple input pathways increases cellular survival in stress. *Elife* **6**, (2017).

14. Wurgler-Murphy, S. M., Maeda, T., Witten, E. A. & Saito, H. Regulation of the Saccharomyces cerevisiae HOG1 mitogen-activated protein kinase by the PTP2 and PTP3 protein tyrosine phosphatases. *Mol. Cell. Biol.* **17**, 1289–1297 (1997).

15. Mettetal, J. T., Muzzey, D., Gómez-Uribe, C. & van Oudenaarden, A. The frequency dependence of osmo-adaptation in Saccharomyces cerevisiae. *Science* **319**, 482–484 (2008).

16. Kanshin, E., Bergeron-Sandoval, L.-P., Isik, S. S., Thibault, P. & Michnick, S. W. A cell-signaling network temporally resolves specific versus promiscuous phosphorylation. *Cell Rep.* **10**, 1202–1214 (2015).

17. Sharifian, H. *et al.* Parallel feedback loops control the basal activity of the HOG MAPK signaling cascade. *Integr. Biol.* **7**, 412–422 (2015).

18. Vaga, S. *et al.* Phosphoproteomic analyses reveal novel cross‐modulation mechanisms between two signaling pathways in yeast. *Mol. Syst. Biol.* **10**, 767 (2014).

19. Jacoby, T. *et al.* Two protein-tyrosine phosphatases inactivate the osmotic stress response pathway in yeast by targeting the mitogen-activated protein kinase, Hog1. *J. Biol. Chem.* **272**, 17749–17755 (1997).

20. Mattison, C. P. & Ota, I. M. Two protein tyrosine phosphatases, Ptp2 and Ptp3, modulate the subcellular localization of the Hog1 MAP kinase in yeast. *Genes Dev.* **14**, 1229–1235 (2000).

21. Durandau, E., Aymoz, D. & Pelet, S. Dynamic single cell measurements of kinase activity by synthetic kinase activity relocation sensors. *BMC Biol.* **13**, 1–16 (2015).

22. Aymoz, D., Wosika, V., Durandau, E. & Pelet, S. Real-time quantification of protein expression at the single-cell level via dynamic protein synthesis translocation reporters. *Nat. Commun.* **7**, 1–12 (2016).

23. Ghaemmaghami, S. *et al.* Global analysis of protein expression in yeast. *Nature* **425**, 737–741 (2003).
