# Supplementary material for: Positive feedback induces switch between distributive and processive phosphorylation of Hog1
Source: Nat Commun. 2023 Apr 29;14:2477. doi: 10.1038/s41467-023-37430-y (PMC10148820; doi:10.1038/s41467-023-37430-y)
Supplement: Supplementary file 2 — Reporting Summary [file 41467_2023_37430_MOESM2_ESM.pdf]

nature portfolio

Corresponding author(s): Manfred Claassen

Last updated by author(s): Feb 10, 2023

Reporting Summary

Nature Portfolio wishes to improve the reproducibility of the work that we publish. This form provides structure for consistency and transparency in reporting. For further information on Nature Portfolio policies, see our Editorial Policies and the Editorial Policy Checklist.

Statistics

For all statistical analyses, confirm that the following items are present in the figure legend, table legend, main text, or Methods section.

n/a	Confirmed	
☐	☒	The exact sample size (*n*) for each experimental group/condition, given as a discrete number and unit of measurement
☐	☒	A statement on whether measurements were taken from distinct samples or whether the same sample was measured repeatedly
☐	☒	The statistical test(s) used AND whether they are one- or two-sided<br>*Only common tests should be described solely by name; describe more complex techniques in the Methods section.*
☒	☐	A description of all covariates tested
☐	☒	A description of any assumptions or corrections, such as tests of normality and adjustment for multiple comparisons
☐	☒	A full description of the statistical parameters including central tendency (e.g. means) or other basic estimates (e.g. regression coefficient) AND variation (e.g. standard deviation) or associated estimates of uncertainty (e.g. confidence intervals)
☐	☒	For null hypothesis testing, the test statistic (e.g. *F*, *t*, *r*) with confidence intervals, effect sizes, degrees of freedom and *P* value noted<br>*Give P values as exact values whenever suitable.*
☒	☐	For Bayesian analysis, information on the choice of priors and Markov chain Monte Carlo settings
☒	☐	For hierarchical and complex designs, identification of the appropriate level for tests and full reporting of outcomes
☒	☐	Estimates of effect sizes (e.g. Cohen's *d*, Pearson's *r*), indicating how they were calculated

Our web collection on statistics for biologists contains articles on many of the points above.

Software and code

Policy information about availability of computer code

Data collection	Model construction and ODE parameter optimization was done in MATLAB 8.5 using the Data 2 Dynamics Software package (arFramework3, def-version 3, c-version code_160823, Copyright 2016 D"D Development Team, All rights reserved.). Computation was carried out on the Euler cluster of ETH Zurich. Data handling and pipeline set up was done via custom bash and MATLAB scipts. Quantification of westen blots was done using ImageJ 1.54b.
Data analysis	Statistical data analysis and visualization was carried out in MATLAB 9.8.

For manuscripts utilizing custom algorithms or software that are central to the research but not yet described in published literature, software must be made available to editors and reviewers. We strongly encourage code deposition in a community repository (e.g. GitHub). See the Nature Portfolio guidelines for submitting code & software for further information.

Data

Policy information about availability of data

All manuscripts must include a data availability statement. This statement should provide the following information, where applicable:

- Accession codes, unique identifiers, or web links for publicly available datasets
- A description of any restrictions on data availability
- For clinical datasets or third party data, please ensure that the statement adheres to our policy

Source data are provided with this paper. The fluorescence microscopy data generated in this study have been deposited in the ETH Research Collection database under accession code 20.500.11850/597634 (DOI: 10.3929/ethz-b-000597634). The models and best fitting parameter values generated in this study have been

deposited in the BioModels database under accession code MODEL2206230001 (https://www.ebi.ac.uk/biomodels/MODEL2206230001).

Human research participants

Policy information about studies involving human research participants and Sex and Gender in Research.

Reporting on sex and gender	n/a
Population characteristics	n/a
Recruitment	n/a
Ethics oversight	n/a

Note that full information on the approval of the study protocol must also be provided in the manuscript.

Field-specific reporting

Please select the one below that is the best fit for your research. If you are not sure, read the appropriate sections before making your selection.

- [x] Life sciences
- [ ] Behavioural & social sciences
- [ ] Ecological, evolutionary & environmental sciences

For a reference copy of the document with all sections, see nature.com/documents/nr-reporting-summary-flat.pdf

Life sciences study design

All studies must disclose on these points even when the disclosure is negative.

Sample size	No sample size calculation was performed. For fluorescence microscopy the maximal number of single cells were imaged given the constraints of sampling frequency and the acquistition time of each image. This resulted in a few hundred to 1000 cells per experiment. After data exclusion (see below) the resulting cells still numbered in their hundreds and were very consistent in their dynamics with histograms showing normal distribution of only little noise. For western blot measurements the field-wide established 3 independent expeirments were performed. Due to higher noise in the pull down experiments, here an additional 4 independent experiments (total of 7) were performed.
Data exclusions	Data were excluded on the basis of nuclear marker intensity (out-of-focus cells), consistency of acquisition over the whole time period (success of cell segmentation), extreme cell size (failure of cell segmentation), and on the basis of extreme variance of nuclear or cytosolic fluorescence over the whole time period (failures of cell segmentation). To our knowledge this is well in line with established protocols of yeast fluorescence microscopy.
Replication	At least three biological replicates were carried out on at least 2 different microfluidic chips, with image acquisition on the same microscope. Findings were reproducible and statistically significant even upon sub-sampling. Similarly, for antibody based measurements at least three replicates were measured for which replication was successfull.
Randomization	This is not relevant to our study as our experimental group consisted of either mutated or WT cells subjected to the same procedures.
Blinding	Blinding was not relevant in this study. Care was taken that both groups in question (WT and mutant) were imaged on the same chip and that temporal sequence of fluorescence image acquistion was shuffled so as not to introduce bias. Differences of interest were determined to be significant using statistical procedures.

Reporting for specific materials, systems and methods

We require information from authors about some types of materials, experimental systems and methods used in many studies. Here, indicate whether each material, system or method listed is relevant to your study. If you are not sure if a list item applies to your research, read the appropriate section before selecting a response.

Materials & experimental systems

n/a	Involved in the study	
	x	Antibodies
x		Eukaryotic cell lines
x		Palaeontology and archaeology
x		Animals and other organisms
x		Clinical data
x		Dual use research of concern

Methods

n/a	Involved in the study	
x		ChIP-seq
x		Flow cytometry
x		MRI-based neuroimaging

Antibodies

Antibodies used	primary antibody for phospho-Hog1 (Cell Signaling, 9215S)<br>total Hog1 (Santa Cruz, SC-165978)<br>secondary antibodies for mouse (LI-COR 926-32212)<br>rabbit (LI-COR, 926-68073).<br>anti-Pbs2 antibody (Santa Cruz, sc-6813)
Validation	Antibodies have been procured commercially and thus have been validated by the company.<br>primary antibody for phospho-Hog1 (Cell Signaling, 9215S): Performance guarantee: https://www.cellsignal.com/about-us/cst-antibody-performance-guarantee<br>total Hog1 (Santa Cruz, SC-165978): Antibody used in 14 previous publications: https://www.scbt.com/p/hog1-antibody-d-3<br>anti-Pbs2 antibody (Santa Cruz, sc-6813): used in such publications as: Thomson et al, 2011 (https://doi.org/10.1073/pnas.1004042108)<br>secondary antibodies for mouse (LI-COR 926-32212): Statement of testing and validation: https://www.licor.com/bio/reagents/irdye-800cw-donkey-anti-mouse-igg-secondary-antibody<br>rabbit (LI-COR, 926-68073): Statment of testing and validation: https://www.licor.com/bio/reagents/irdye-680rd-donkey-anti-rabbit-igg-secondary-antibody
